# Modern breeding of root, tuber, and bulb crops to enhance food and nutritional security

**DOI:** 10.1007/s00122-026-05426-z

**Published:** 2026-11-04

**Authors:** Rodomiro Ortiz, Sangam L. Dwivedi, Audrey Pelikan, Irwin L. Goldman

**Affiliations:** 1 Department of Plant Breeding, Swedish University of Agricultural Sciences, Alnarp, Sweden; 2 Independent Researcher, Hyderabad, 500016 Telangana India; 3 Department of Plant and Agroecosystem Sciences, College of Agricultural and Life Sciences, University of Wisconsin-Madison, 1575 Linden Drive, Madison, WI 53706 USA

## Abstract

***Key message*:**

**Unlocking the molecular mechanisms and pathways of underground storage organ development and nutrient and non-nutrient traits may provide more avenues to tailor elite germplasm enriched with these characteristics in root, tuber, and bulb crops. Biofortified cassava, potato, and sweet potato provide food and nutritional security to millions of people in Africa, Asia, and Latin American regions.**

**Abstract:**

Root and tuber crops provide food and nutritional security to millions of people in Africa, Asia, and Latin America and the Caribbean, while garlic and onions, in addition to strong therapeutic potential, are widely used in food preparation to add flavour and taste. Root, tuber, and bulb (RTB) crops are a good source of carbohydrates, minerals, and bioactive compounds. However, these crops also possess a variety of allergens and toxic compounds, limiting their consumption. Here, we synthesized i) the contribution of RTB crops on food and nutrition security in the Global South, ii) the various types of allergens and toxic compounds detected in these crops, iii) catalogue germplasm resources across national and international genebanks, iv) document the range of genetic variation in edible organs morphology, nutrient and non-nutrient traits, v) discuss advances in high-throughput phenomics to monitor variability, vi) assess population structure and diversity in germplasm pool, vii) list genomic regions (QTL) and genes (both functionally characterized and candidate genes and QTL) associated with storage organs (roots or tubers) morphology, nutrient and non-nutrient traits, viii) uptake, transport, and storage of minerals in edible vegetable organs and their effects on consumers, and ix) advances in genetic improvement of nutritionally enhanced (nutrient and non-nutrient traits) and productive germplasm through crossbreeding and biotechnological interventions. Assessing and exploiting functional diversity, combined with the use of functionally characterized genes and associated markers for storage root growth/thickening, and nutrient and non-nutrient traits, can be deployed in breeding programs to improve these characteristics in RTB crops. Numerous candidate genes and genomic regions (or quantitative trait loci) reported herein must be functionally validated before being deployed in crop breeding programs. A greater emphasis should be placed on genetically and phenotypically characterizing RTB germplasm, and on combining empirical breeding with molecular tools to enhance precision, efficiency, and rapid genetic gain. Millions of rural households in parts of Africa, Asia, and Latin America are currently benefiting from the commercialization of provitamin A-enriched cassava, potato, and sweet potato. The development of orange-fleshed sweet potato cultivars with higher levels of provitamin A was awarded the ‘‘World Food Prize’’ in 2016. There are more opportunities to enhance nutrition status and food security through improvement of root, tuber, and bulb crops.

## Nutritional quality of root, bulb, and tuber (RTB) crops to enhance food and nutritional security

Globally, root, tuber and bub crops –(RTB) including carrot (*Daucus carota*), cassava (*Manihot esculenta*), radish (*Raphanus sativus*), sugar beet (*Beta vulgaris* subsp. *vulgaris*), sweet potato (*Ipomoea batatas*), taro (*Colocasia esculenta*), and turnip (*Brassica napus rapifera*) among root crops and potato (*Solanum tuberosum*) and yam (*Dioscorea* spp.) among tuber crops provide food and nutritional security to millions because of their high caloric efficiency and resilience in marginal environments. Garlic (*Allium sativum*) and onions (*Allium cepa*), among bulb crops with strong therapeutic potential (Bedir et al. 2025), are widely used in food preparations to add flavour and taste. These crops are the second most important source of carbohydrates after cereals (Table 1), and they often surpass grains in energy yield per unit of land; for example, cassava and yams can produce significantly more calories per hectare than rice or wheat (Montagnac et al. 2009). In addition to serving as "energy banks," RTCs provide essential dietary fibre and micronutrients that are frequently lacking in cereal-based diets. For example, taro is notable for its highly digestible starch and high potassium content, while many tubers supply substantial amounts of vitamin C, which can enhance the absorption of non-heme iron (Nigussie 2025; Temesgen 2015). Moreover, RTCs contain bioactive phytochemicals—such as phenolic compounds, betalains, and saponins—that offer functional health benefits, including antioxidant and anti-inflammatory effects, which may help reduce the risk of chronic diseases associated with food insecurity (Chandrasekara and Kumar 2016). Likewise, garlic and onions also provide non-nutrient compounds (phytochemicals) with protective effects that can support healthy blood sugar levels, improve heart health, and fight harmful microbes (Bedir et al. 2025).

**Table 1:** Nutritional comparison of major root and tuber crops (per 100 g)

Crop	Energy (kcal)	Vitamin A (IU)	Vitamin C (mg)	Iron (mg)	Potassium (mg)	Zinc (mg)
Cassava	160	13	20.6	0.27	271	0.34
Sweet potato (orange flesh)	86	14,187	2.4	0.61	337	0.30
Potato (white flesh)	77	7	19.7	0.78	421	0.29
Yam	118	138	17.1	0.54	816	0.24
Taro	112	Trace	3.6	0.55	591	0.23

FAO (1990), USDA (2025)

Enhancing nutritional security through RTCs is increasingly achieved via biofortification—a process that involves breeding crops to increase their content of essential vitamins and minerals—as a sustainable strategy to address "hidden hunger," or the widespread problem of micronutrient deficiencies that occur even when caloric intake is sufficient (Avnee et al. 2023). The widespread adoption of orange-fleshed sweet potatoes (OFSP) in sub-Saharan Africa has been associated with improved vitamin A intake among vulnerable groups, thus demonstrating the potential of breeding for high-carotene content (Low and Carvey 2020). Similarly, ongoing research into biofortified cassava and iron-rich potatoes aims to provide long-term, cost-effective solutions to anaemia and stunting without requiring drastic changes to traditional dietary habits (Dhaliwal et al. 2022; Dwivedi et al. 2023). These crops are particularly vital for smallholder farmers because they can be harvested progressively, serving as a natural food reserve during lean seasons or periods of climate-induced crop failure. Integrating nutrient-dense RTC cultivars into local agrifood systems may help improve both the caloric stability and the micronutrient profile of diets in the world’s most resource-challenged regions (Sanginga 2015).

In addition, the indigenous Andean farmers domesticated a variety of other RTB crops, localized in the region, and include arracacha (*Arracacia xanthorrhiza*), achira (*Canna indica*), yacon (*Smallanthus sonchifolius*), mauka (*Lepidium meyenii*), ahipa (*Pachyrhizus ahipa*), ulluco (*Ullucus tuberosus*), oca (*Oxalis tuburosa*), mashua (*Tropaeolium tuberosum*), and several species of potatoes. These crops are grown from southern Venezuela to northwestern Argentina, with the highest varietal diversity and uses in the region between central Peru and central Bolivia. They are rich sources of secondary metabolites and antioxidant properties, thereby providing immense health benefits. Native potatoes belong to 7 species that exhibit a variety of forms, colours, and textures after boiling (Roca et al. 2007). The indigenous RTB crops grown in the Andean region are good sources of functional foods (Leidi et al. 2018). All plant parts are used in a variety of forms, with rhizomes being the most consumed due to their high dietary fibres and starches, vitamins, minerals, and antioxidant capacity. The tubers are essentially gluten-free for the development of functional foods for those patients with gluten sensitivity (Arcos et al. 2025).

The scientific literature was reviewed from 2000 to date to identify peer-reviewed publications using keywords and phrases for an in-depth synthesis from the Web of Science database. The included keywords and phrases were germplasm, genebank, phenomics, genetic variation, population structure and diversity, association mapping diversity panels and specialized populations, intraspecific and interspecific variation, sequencing genebank diversity, and core collections. Genome-wide association studies (GWAS), DNA markers, quantitative trait loci (QTLs), and putative (or candidate) and functionally characterized genes were additional keywords. Likewise, uptake, transport and storage of nutrient and non-nutrient traits, pathways associated with storage organ development and nutrient accumulation, allergens and toxic compounds, and sensory perception and consumer acceptance, which were also used in the literature search. Equally important keywords were advances in genetic improvement of nutritional traits following empirical breeding, marker-assisted selection, predictive breeding, genome editing, biofortification, and metabolic engineering. The major focus was toward i) the contribution of RTB storage crops on food and nutrition security in the *Global South*, ii) documenting allergens limiting the consumption of root, bulb, and tuber crops in the human diet, iii) cataloguing and documenting root, bulb, and tuber crops germplasm resources across national and international genebanks, iv) genetic variation for edible organmorphology, nutrient and non-nutrient traits, v) advances in high-throughput phenomics to monitor variability for morphology, nutrient and non-nutrient traits, vi) assessing population structure and diversity in germplasm pools, vii) genomic regions (QTL) and genes associated with morphology, nutrient and non-nutrient traits, viii) nutrient and non-nutrient uptake, transport, and storage in edible vegetable organs and their effects on consumers, and finally on ix) advances in genetic improvement of nutritionally (both nutrient and non-nutrient traits) and productive germplasm to enhance food and nutritional security in the *Global South*.

## Allergens and toxic compounds limiting the consumption of RTB crops

Although RTB crops are undeniably important for enhancing food and nutritional security and are generally considered low in allergenicity, the presence of certain toxins and allergens can pose limitations to their consumption. In some RTB crops, these compounds function as plant defence mechanisms and are often associated with bitter taste. Cyanogenic glycosides are present in all cassava germplasm and plant parts. These compounds, stored as linamarin and lotaustralin, are synthesized in the leaves from the amino acid precursors valine and isoleucine, respectively. While not inherently harmful, disruption of cellular structures can trigger hydrolysis of linamarin and lotaustralin, and under low pH conditions, hydrogen cyanide is released. This can be poisonous to consumers, and when ingested in high volumes or combined with conditions like low protein intake and undernutrition, it can cause konzo, a paralysis of the legs. Strategies to reduce this risk include breeding efforts – variability has been observed, and early detection methods and gene editing approaches exist – and, most importantly for the Global South, proper preparation methods (Bokanga 1994; Gomez et al. 2023; Nambisan 2011). Significant reductions in cyanogen have been observed when tubers are processed through boiling, drying, soaking, blanching, crushing, baking, frying, or peeling, with the most effective methods being grating followed by fermentation or sun drying, which can lead to 95–99% removal of cyanogen (Nambisan 2011).

Similarly, glycoalkaloids in potato, primarily solanine and chaconine, are a natural toxin found throughout all organs of the plant, with the highest concentrations in sprouts, peel, green skin, and eyes. These compounds can irritate the gastrointestinal tract and, under high consumption, cause neurological symptoms. Glycoalkaloids accumulate when potatoes are exposed to light or mechanical injury, which is why in-season and post-harvest storage management are crucial to potato nutritional value. Although less concentrated in the potato flesh, strategies such as breeding for resistance to greening, bruising, and postharvest glycoalkaloid accumulation, reducing light exposure in the field, maintaining cool and dark storage conditions, and peeling or removing damaged areas before cooking can minimize risk. It is worth noting that glycoalkaloids have demonstrated antimicrobial and anticancer properties under carefully controlled dosages and may have future medicinal applications (Figueira et al. 2026; Torabian et al. 2023).

Allergens do not cause poisonous responses like the above toxins, but they can limit consumption for sensitive individuals, mainly through hypersensitivity to the IgE antibody. In potato, the storage protein patatin (Sola t 1) functions as a reserve for sprouting and may elicit an allergenic response, such as itchy mouth or hives (Breiteneder and Radauer 2004). Oral Allergy Syndrome (OAS) occurs when IgE antibodies generated against pollen proteins cross-react with structurally similar proteins in raw fruits and vegetables. For example, raw potatoes and carrots contain defence proteins, including PR-10 type proteins and profilins, which are structurally similar to pollen allergens, especially birch pollen (Bet v 1). Symptoms typically include irritation of the mouth and throat, as well as swelling of the lips or tongue. This allergy can often be mitigated by cooking, where heat denatures the problematic proteins, and is most relevant to regions where birch pollen is prevalent. Similarly, PR-4 type proteins (Bra r 2) identified in turnip serve defensive roles against pathogens but also provoke OAS due to cross-reactivity with latex (Breiteneder and Ebner 2000; Breiteneder and Radauer 2004; Tolkki et al. 2013). Finally, irritant compounds are present in several RTB crops such as oxalates in table beet, yam, and taro and sulphur compounds in onion and garlic that can cause contact dermatitis, particularly during meal preparation.

## Cataloguing and documenting RTB crops germplasm resources across genebanks

Germplasm resources for RTB crops are conserved in both national and international genebanks. National genebanks often preserve regionally adapted germplasm, while the CGIAR centres maintain larger collections of crops essential for food security, nutrition, and income, located in the Global South. Although exceptions exist, these crops are often clonally propagated and highly heterozygous, which means seed storage cannot preserve the specific gene combinations needed by farmers, and widely grown cultivars do not produce seed efficiently. As a result, these collections are often maintained in field genebanks, in vitro, or through more advanced methods like cryopreservation, which can be high in cost and time (Kalia 2025; Pathirana and Carimi 2022). Consequently, many of the modern breeding tools are not yet fully adapted to the genetic architecture and breeding systems of RTB crops, and systems may need to be redesigned for clonally propagated or polyploid crops.

Cultivated cassava (*Manihot esculenta*) was likely domesticated in Amazonia, South America, from its wild progenitors *Manihot esculenta* subsp*. flabellifolia* and *peruviana*, all of which serve as the primary gene pool for genetic exchange. Numerous other *Manihot* species (*M. tryiphylla, M. pilosa*, etc.) constitute secondary gene pools (Allem 2002; Gowda et al. 2025). The largest cassava collections are maintained by CGIAR, with about 6,500 accessions at CIAT and 3,700 accessions at IITA, alongside smaller regional collections (Table 2). Globally, over 10,000 unique cassava accessions are estimated to be conserved ex-situ across more than 70 genebanks, though roughly two-thirds of the species’ genetic diversity remains in situ, both in farmer’s fields and in natural stands. Relatively few improved cultivars exist to date due to the few breeding programs, and farmer’s reliance on many landrace cultivars, which makes germplasm preservation vast (Gowda et al. 2025). Core collections are well defined, including 630 accessions at CIAT, 428 at IITA, and others based on SNP markers (CGIAR Genebanks Platform n.d.; Santos et al. 2023).

**Table 2:** Root, tuber, and bulb crop genetic resources and respective key descriptors

Crop	Center of origin	Primary gene pool	Major germplasm collections	Key descriptors*		
				Above ground	Below ground	Traits
Garlic (*Allium sativum*)	Central Asia	*Allium sativum* and wild *Allium* spp.	USDA-NPGS, IPK, INTA	Leaf colour/waxiness, plant height, leaf length/width	Bulb weight, clove number, clove uniformity	Pungency, storability, bolting tendency
Onion (*Allium cepa*)	Central Asia	*Allium cepa* and its multiple subsp.	USDA-NPGS, VIR, ICAR-NBPGR	Leaf colour/waxiness, leaf length/width, pseudostem diameter/height	Bulb skin colour, bulb shape, bulb size	Pungency, storage life, dry matter content
Carrot (*Daucus carota* subsp. *sativus*)	Central Asia	*Daucus carota* subsp. *sativus* and *carota*	UKVGB, USDA-NPGS	Life cycle, leaf architecture, leaf colour	Market class root shape, root colour	Soluble solids, carotenoid content, cracking resistance
Cassava (*Manihot esculenta*)	Amazonia, South America	*Manihot esculenta* and its multiple subspecies	CIAT, IITA, EMBRAPA	Growth habit, leaf lobe number, petiole colour	Root shape, root number, root size	Cyanogenic potential, starch content, cooking quality
Radish (*Raphanus sativus*)	Mediterranean and Middle East	*Raphanus sativus* in cultivated, wild, or weedy forms	VIR, CGRIS	Leaf colour, leaf margin, plant height	Root shape, root size, root skin and flesh colour	Pungency, root texture, bolting tendency
Sweet Potato (*Ipomoea batatas*)	Central and South America	Cultivated and wild *Ipomoea batatas*	CIP, USDA-NPGS	Vine growth habit, leaf lobing, leaf colour	Root shape, root size, root skin and flesh colour	β-carotene, sweetness, dry matter content
Table Beet (*Beta vulgaris* subsp. *vulgaris*)	Mediterranean	*Beta vulgaris* complex, including wild relative *Beta vulgaris* subsp. *maritima*	USDA-NPGS, European genebanks	Annuality, leaf colour, petiole color	root shape, root color, root size	Soluble solids, betalain pigment, foliage strength
Taro (*Colocasia esculenta*)	Southeast Asia	*Colocasia esculenta* and its multiple subsp.	CePaCT	Leaf blade size, leaf shape, petiole colour	Corm size, corm shape, corm flesh colour	Oxalate content, cooking quality, taste
Turnip (*Brassica rapa* subsp. *rapa*)	Western Asia, Europe	*Brassica rapa*, including other cultivated and wild forms	IPK, USDA-NPGS	Leaf lobing, leaf color, rosette size	Root shape, root size, root skin and flesh colour	Core size, taste, fiber content
Potato (*Solanum tuberosum*)	Andes, South America	Cultivated potato, S*olanum tuberosum*, which are 4× and 4EBN	CIP, INRA-Rennes, VIR	Growth habit, leaf type, stem pigmentation	Tuber shape, tuber size, tuber skin and flesh colour	Storability, glycoalkaloids, reducing sugar content
Yam (*Dioscorea* spp.)	West Africa, Southeast Asia	*Dioscorea* spp., both wild and cultivated	IITA, CePaCT, PGRRI	Vine growth habit, leaf shape, winging	Tuber shape, tuber size, flesh colour	Oxidation tendency, bitterness, texture

*Examples of key descriptors, not a comprehensive list, and can vary depending on growing region and interest

The cultivated potato (*Solanum tuberosum*) was domesticated in the Andes mountains of South America. Its gene pool is complex, and compatibility is often classified by Endosperm Balance Number (EBN), with five primary, 68 secondary, and 23 tertiary wild species identified, with even more uncharacterized. Potatoes can grow across a wide range of altitudes, precipitation, and soils, generating numerous wild relatives, many of which are yet to be collected. While particularly important for the Global South, potato is globally significant, and large genebank collections exist worldwide: INRA-Rennes, France (10,500 accessions), VIR, Russian Federation (8,900), and CIP, Lima, Peru (7,500), all under medium- to long-term storage conditions. Globally, approximately 98,285 potato accessions are conserved ex situ in 174 genebanks, representing an estimated 24,500–29,500 unique accessions (Dalamu et al. 2025; Vollmer et al. 2022). The international genebank, CIP, has assembled 451 diverse accessions for a core collection and a subset mini core of 45 diverse landraces (International Potato Center 2019).

The primary gene pool of sweet potato includes *Ipomoea batatas* and its closest wild relatives, particularly *Ipomoea trifida*, which can readily intercross. More distantly related *Ipomoea* species form the secondary and tertiary gene pools with increasing reproductive barriers such as ploidy, self- and cross-incompatibility, and large chromosomal number, which are made more prominent by the northern (Caribbean and Central America) and southern (Peru-Ecuador region of South America) geographically restricted gene pool clusters (Pati et al. 2025; Pereira et al. 2023; Roullier et al. 2013). The largest sweet potato collection is maintained by CIP, which conserves over 5,600 in vitro accessions from 57 countries, primarily from Latin America, followed by the USDA-NPGS collection in Griffin, Georgia, USA. It is estimated that more than 35,000 sweet potato accessions are held in germplasm banks globally, although fewer than 10% of the crop’s wild relatives are represented (Jarret et al. 2019; Pati et al. 2025). CIP has established a core collection of 140 accessions and a mini core of 16, although these represent only the virus-free portion of the germplasm and may have limitations in genetic representation (International Potato Center n.d.).

The most important edible yams, *Dioscorea* spp., come from multiple species, including *D. rotundata, D. alta, D. cayenensis, D. esculenta, D. dumetorum, D. bulbifera, D. trifida, D. opposita, and D. japonica*. There are an estimated 600 species of *Dioscorea*, many of which are wild relatives and remain underrepresented in germplasm collections. Yams evolved in independent regions, resulting in much diversity (CGIAR Genebanks Platform n.d.; Nduche et al. 2024). West Africa is particularly important as the centre of origin for the Guinea yams (*D. rotundata* and *D. cayenensis*), which were likely domesticated from *D. praehensilis* and form the majority of IITA’s collection. The Centre for Pacific Crops and Trees (CePaCT) in Fiji also maintains a significant collection. Six of the cultivated species are represented in the core collection, which includes 843 accessions and accounts for roughly 20% of the full collection (Genesys n.d.; Girma et al. 2017). Additionally, the CGIAR hosts a research program on roots, tubers, and bananas, which focuses specifically on cassava, yams, potatoes, and sweet potatoes to better utilize available genetic resources for improved livelihoods (https://www.rtb.cgiar.org/).

Many other RTB crops are conserved regionally, rather than at global scales, reflecting their localized importance. Although many accessions may be in the collection for a specific crop, only some are available for distribution. For example, the USDA-NPGS *Daucus* spp. collection holds 1,565 accessions, yet only 1,306 are available for distribution, and 31 of the unavailable accessions have been inactivated. These limitations reflect several common challenges associated with RTB germplasm management, including limited or disease-infested seed or plant material, taxonomic uncertainty, differences in annual versus biennial life cycles, unsuitable regeneration environments, and specialized expertise required to maintain certain wild species. Verifying taxonomy and securing enough seeds for distribution may take multiple years of regeneration (K. Reitsma, personal communication, 2026). Characterization is also a limiting factor in utilization. For example, across 280 genebanks in 62 countries, only 46% of *Allium* bulb accessions have been characterized, with an average of 15 traits recorded per accession. In tuber crops, characterization levels are even lower, with 33% of potato and 28% of yam accessions characterized, although these include relatively higher numbers of traits per accession, averaging 37 and 47, respectively. For root crops, characterization ranges from 15 to 50%, with an average of 32%, and between 26 and 50 traits per accession, with an average of 37 (excluding table beet and radish, for which values are not defined) (FAO 2023; Table 2). Together, these limitations highlight a substantial gap between the conservation of RTB diversity and its effective utilization in breeding. Addressing this gap will require improved genomic and phenotypic characterization, development of core collections, resolution of taxonomic uncertainty and duplication, and pre-breeding to transfer useful diversity from wild relatives into accessible breeding materials. These approaches are well developed in numerous other crops. For example, genetic characterization of the USDA rice genebank contributed to the development of a mini-core collection designed to facilitate more efficient evaluation and utilization of rice diversity (Agrama et al. 2009), among other developments. Similar approaches should be expanded across RTB crops to access the diversity already conserved in gene banks.

## Phenomics to monitor variability in storage organ morphology, nutrient and non-nutrient traits

Among the largest challenges in phenomic assessment of root, tuber, and bulb crops is precise estimation of below-ground traits, which are notoriously difficult to measure. Thus, phenomic assessment of RTB crops generally falls behind assessments in above-ground crops. Because phenomics encompasses the full set of phenotypes that any given genotype may express in relation to genotype, developmental stage, and environment (Zavafer et al. 2023), phenomic characterization is particularly challenging for RTB crops. This is partly due to the dearth of data on productivity traits for these crops. Traditionally, it has been time and resource-intensive to extract these crops from the soil and prepare them for data collection. Pioneering work in cereal crops has led to techniques such as shovelomics, which is used to remove crops from the soil and produce digital images that can be used for developing predictive datasets (Arifuzzaman et al. 2019). Similar approaches for root, tuber, and bulb crops would facilitate future phenomic efforts. Technologies that can assess the underground portion of the crop in a non-invasive way would be most helpful.

*Phenotyping storage root size and shape, and bulb morphology*: The size and shape of storage roots are crucial in the development of storage root crops. However, such traits are highly influenced by genetic and environmental factors, affecting edible yield, nutrition, and marketability. Hence, understanding the mechanisms underlying storage root formation and development is crucial. A complex biological process, involving morphology, physiology, and genetics, is involved in storage root size and shape (Yang et al. 2023). Counting and measuring the size of storage roots below the soil is a significant challenge in early-generation breeding cycles. A convolutional neural net-based cassava storage root counting system uses real and synthetic images to count the number of storage roots in real cassava images, obtained from a low-cost aeroponic (Atanbori et al. 2019) growth system. The model first predicts age group (young or old roots) and then selects an appropriate model to predict the number of storage roots. Following this, 91% accuracy was achieved in predicting storage root age, while 86% and 71% agreement on counting old and young storage roots, respectively (Atanbori et al. 2019). A digital-image-based phenotyping platform captured over 80% of the variation and significant narrow-sense heritability for root size and shape in carrots (Brainard et al. 2021a). Ground-penetrating radar (GPR) is a novel non-destructive, high-throughput, 3-dimensional imaging method to predict root yield efficiently and accurately by combining above- and below-ground time series information. GPR automates images via image acquisition hardware to achieve quality images for multiple accessions or breeding populations, tailored for cassava, but with potential application in other RTB crops (Fernando et al. 2022). Unmanned Aerial Vehicles (UAVs), such as CIAT pheno-i (a non-descriptive and cost-effective assay), with multispectral sensors, rapidly monitor canopy metrics and vegetation indices (VIs) to effectively predict root yields in cassava (Selvaraj et al. 2020). Wilhelm et al. (2022) introduced a method that effectively analyses the distribution of root diameters automatically, regardless of root systems displaying substantial variability in root width and clustering, which detected wide variation in root diameters and clustering in cassava germplasm. The dynamics of root type formation can be monitored qualitatively and quantitatively. It is a fast and robust analytical platform to detect variations in complex root systems, thereby applicable in high-throughput phenotyping and future breeding of cassava and other root crops. A digital imaging tool has been standardized to automatically measure bulb morphology-geometrical properties in onion (Kim et al. 2025) or 3D imaging techniques in garlic (Son et al. 2026) germplasms.

*Non-invasive phenomics and molecular analyses monitor variability in root nutritional traits*: Traditional methods of nutritional assessment are time-consuming and difficult to scale to large numbers of plants. High-throughput technologies are necessary in any large-scale breeding program, such as the protein assessment provided by Laurie et al. (2020) in South Africa. The integration of metabolic and proteomic profiling within a comprehensive “panomics” framework—which combines multiple omics approaches—has the potential to further enhance our understanding of the chemical basis of root nutritional quality. While traditional phenotyping focuses on physical structure, molecular phenomics utilizes analytical tools such as liquid chromatography-mass spectrometry (LC–MS) and nuclear magnetic resonance (NMR) to characterize the global metabolite pool and protein networks that determine nutrient density (Razzaq et al. 2019; Singh et al. 2022a). These molecular profiles serve as functional readouts of cellular processes, enabling the identification of biomarker metabolites and candidate proteins that regulate the accumulation of vitamins, amino acids, and minerals (Kumar et al. 2017; Raza et al. 2024). By using metabolite-QTL (mQTL) analysis—which links variations in metabolite levels to specific genetic loci (Dumas and Gauguier 2012)—plant breeders can more accurately select for nutritional traits. This approach accelerates the development of biofortified cultivars that are resilient to fluctuating climate conditions and soil nutrient deficiencies. It is important to note that nutritional improvement does not guarantee nutritional impact. This argues for breeding approaches to include sensory evaluation and pay attention to consumer-facing traits. These efforts will help ensure that the resources spent on improving nutritional content will have the best possible chance of delivering nutritional impact.

The integration of metabolic and proteomic profiling within a comprehensive “panomics” framework—which combines multiple omics approaches—has the potential to further enhance our understanding of the chemical basis of root nutritional quality. While traditional phenotyping focuses on physical structure, molecular phenomics utilizes analytical tools such as liquid chromatography-mass spectrometry (LC–MS) and nuclear magnetic resonance (NMR) to characterize the global metabolite pool and protein networks that determine nutrient density (Razzaq et al. 2019; Singh et al. 2022a, b, c). These molecular profiles serve as functional readouts of cellular processes, enabling the identification of biomarker metabolites and candidate proteins that regulate the accumulation of vitamins, amino acids, and minerals (Kumar et al. 2017; Raza et al. 2024). By using metabolite-QTL (mQTL) analysis—which links variations in metabolite levels to specific genetic loci (Dumas and Gauguier 2012)—plant breeders can more accurately select for nutritional traits. This approach accelerates the development of biofortified cultivars that are resilient to fluctuating climate conditions and soil nutrient deficiencies.

Phenotyping remains the fundamental bottleneck for RTB crops, driven by the inherent difficulty of non-invasively assessing underground storage organs. This generates a pronounced genome-to-phenome gap, marked by a sharp disconnect between increasingly sophisticated genomic datasets and comparatively crude phenotyping methods. To make genomic prediction and genetic dissection truly effective for RTB crops, future efforts must prioritize the development of accurate, affordable, and scalable phenotyping solutions to bridge this divide.

*Phenotyping challenges for culinary traits:* Above-ground imaging tools have also been used successfully to predict traits like biomass and seed yield, and to some extent, below-ground traits like root yield. These tools may include unmanned aerial vehicles (UAVs), multispectral and hyperspectral cameras, autonomous platforms, and tractor-based imaging systems. In these systems, multispectral imagery combined with UAVs has been used successfully to understand growth patterns of crops such as cassava. It is possible to estimate canopy cover and canopy volume, which can then be used to estimate crop productivity, but characterization of root traits is more difficult. Selvaraj et al. (2019) developed an aeroponic system for real-time monitoring of storage root development in cassava, which was coupled with a digital imaging platform for quantifying root growth and development traits. Correlation analysis confirmed that image-based estimation was able to successfully replace manual root phenotyping for several key traits. Ground penetrating radar is a non-destructive, high-throughput method for predicting root traits. Prediction models have been developed using this technology to chart starch accumulation and root growth. X-ray CT and MRI approaches have also been used for assessing root crop traits (Urfan et al. 2022). Traditional methods of nutritional assessment are time-consuming and difficult to scale to large numbers of plants. High-throughput technologies are necessary in any large-scale breeding program, such as the protein assessment provided by Laurie et al. (2020) in South Africa.

As discussed earlier, root traits are typically more difficult than shoot traits for a phenomic assessment (Yang et al. 2020). Near-infrared spectroscopy (NIRS) has been used for high-throughput phenotyping of plant samples in a range of crop species. NIRS has been used for assessing starch, sugar, protein, and fibre in sweet potato (He et al. 2022). Reducing sugars are important quality factors for assessing the edible quality and nutritional status of sweet potato. Quantification and characterization of carotenoids in plant tissue samples is extremely time-consuming and expensive. de Carvalho et al. (2022) developed a high-throughput phenotyping system that made use of digital images of pulp from cassava roots. The images were then correlated with total carotenoid concentration measured using traditional methods and used to develop prediction models. They found a positive correlation between carotenoid concentration and certain RGB colour parameters. They also developed an artificial neural network, which had the best predictive ability for carotenoid concentration. These tools offer an economical and time-saving method for characterizing large numbers of cassava root samples for a nutritionally significant trait such as carotenoid concentration.

Cassava is a crucial global source of starch. Cassava improvement requires careful quantification of root parameters, such as size and shape. These are typically performed by hand, but Wilhelm et al. (2022) have reported on a new method for root quantification using digital imaging and video. Their method makes use of iterative ridge detection and has been tested using traditional methods of shape and size measurement. Sweet potatoes can be damaged during harvest in a phenomenon known as skinning, in which the epidermal layer of cells is scraped or torn. Bloom et al. (2025) introduced a computer vision pipeline to automate skinning assessment that could estimate the percentage of skinning as the ratio between the predicted skinning area and the total surface area. They found that subsampling was effective in estimating the skinning percentage of entire fields, and that the model itself was quite accurate in assessing skinning percentage on a commercial scale.

## Genetic variation for nutrient and non-nutrient traits in RTB crops genetic resources

Carbohydrates, fats, proteins, and minerals are essential nutrient traits needed for human metabolism and body functions (Ajomiwe et al. 2024; Ioniță-Mîndrican et al. 2022; Matthewman and Costa-Pinto 2023). Non-nutrient traits, such as carotenoids, flavonoids, and phenols, do not provide significant nutritional value or energy (caloric) but, in small quantities, provide significant health benefits or negatively affect human health (Crupi et al. 2023; Matsumura et al. 2023; Zheng et al. 2025). Research to date suggests sufficient variability for nutrients (Table 3) and non-nutrient (Table 4) traits among the analysed germplasm. Traditional (landraces and heirloom) cultivars and wild relatives of RTB crops are more nutritious than modern cultivars. For example, garlic landraces have shown significant superiority in bulb morphology and quality features over modern cultivars within and across regions (Petropoulos et al. 2018a, b). Turnip accessions show exceptional variability for root shape (globose, oblong, ovate, obovate, and fusiform), skin colour (white, bicolour white-violet, light violet, and dark violet), and 3tuber weight (Khadivi et al. 2022). Russet potatoes (large oblong shape, dark brown, netted skins, and white fluffy interior, high in starch and low in moisture), though inferior in mineral content, are ideal for baking, mashing, and frying (Pandey et al. 2023). Colored storage organs of RTB crops are more nutritious than those with white, light yellow, or dark yellow flesh colours. Wild yam species from the Koraput region of India are significantly superior in nutritional profile and better physico-functional parameters than cultivated ones (Pradhan et al. 2018, 2020).

**Table 3:** Genetic variation and commentary for mineral concentrations and major nutritional components in garlic, onion, cassava, taro, turnip, potato and yam genetic resources. Values are presented in alphabetical order within each category

Crop	Minerals	Major nutritional components	Other	Method	Reference
Bulb crops					
Garlic (*Allium sativum*)	Ca 0.6–1.1, K 8.1–17.0, Mg 0.6–1.0, N 18.5–40.3, Na 0.2–0.4, P 3.3–5.7, S 3.4–11.7 g kg^−1^ DW Fe 315–955, Zn 170–676 mg kg^−1^ DW	Fructose 0–3.5, glucose 0–1.6, sucrose 0–13.7 g kg^−1^ DW		Minerals, atomic absorption spectrometry; sugars, HPLC	Sönmez et al. 2025
	Garlic landraces, ‘Aglio di Peschici’ and ‘Aglio Rosso di Monteleone di Puglia’ very rich source of minerals			Minerals, Ion chromatography	Bonasia et al. 2020
	Ca 1.6–55.6, K 4.5–6.8, Na 0.1–0.4, Mg 0.20–0.6 g kg^−1^ FW Fe 280–570, Zn 5.5–15.2 mg kg^−1^ FW	Carbohydrate 230–360, fat 1.2–3.6, protein 46–74 g kg^−1^ FW		Protein (AOAC 2016); carbohydrate (AOAC 2005); fat (AOAC 2016); minerals, atomic absorption spectrophotometry; Na, K, flame photometry	Petropoulos et al. 2018b
Onion (*Allium cepa*)	Ca 0.3–0.5, K 0.4–0.7, S 2.0–8.5 g kg^−1^ DW; Fe 0.8–4.5, Zn 1.8- 5.8, mg kg^−1^ DW			Minerals, atomic absorption spectrometry; S, spectrophotometry	Chandel et al. 2024
		Reducing sugar 54.3–142.6, total sugar 39.0–144.5, TSS^c^ 55.7–101.6 g kg^−1^ FW		TSS, refractometry; TS, reducing sugar, colorimetric	Amir et al. 2023
Root crops					
Cassava (*Manihot esculenta*)		Dietary fibre 12.0–26.0, protein 4.2–8.6, starch 653–728 g kg^−1^ DW	Dry matter 15.2–21.0%	Starch: GB 5009.9–2016; reduced sugar: Response surface method (Zhi-Xia et al. 2016); protein: GB 5009.5–2016; dietary fibre: GB5009.88–2014	Chen et al. 2024
Radish (*Raphanus sativus*)		Substantial diversity in carbohydrate, sugar, and reducing sugar, with some accessions superior across environments		FC (AOAC, 2020); Protein (Lowry et al. 1951); RS (Teixeira and Santos 2022); TC (Tamboli et al. 2020); TS (Ebell 1969); TSS (digital refractometer,°Brix); Vitamin C (Mushtaq et al. 2022);	Tare et al. 2026
		Wide variation detected in monosaccharide and total sugar among VIR genebank radish collection		Sugar (State standard 8756)	Kurina et al. 2021
Sweet potato (*Ipomea batatas*)	Ca 0.6–6.3, K 5.6–24.8, Mg 0.4–2.2, Na 0.5–5.0, P 1.3–4.4 g/kg DW; Cu 3.03–10.4, Fe 100–600, Mn 3.7–37.3, Se 0–0.2, Zn 5.7–17.6 mg/kg DW	Dietary fibre 79.9–260, protein 21.5–71.1, starch 226–697, TSS 103–400 g kg^−1^ DW		Protein: Kjeltec8200instrument (Foss, Hillerød, Denmark) × 6.25; starch: MEGAZYME kit (AOAC 996.11) (Megazyme, Bray, Ireland); total soluble sugar (TSS): Galvao et al. 2021); dietary fibre: Enzymatic gravimetric method (AOAC991.43); minerals: Sun et al. 2011) with modification;	Zhao et al. 2024
		Protein 6.8–115.1 g kg^−1^ DW		Kjeldahl (AOAC 1980) and NIR spectra (Magwaza et al. 2016)	Laurie et al. 2020
Taro (*Colocasia esculenta*)	Eastern Himalayan taro accessions showed substantial variability for minerals			P (Hesse 1971), K (Chapman, Pratt, 1961), Cu, Fe, Mn, Zn (Zasoski and Burau 1977), Ca and Mg (Cheng and Bray 1951)	Talang et al. 2025
		Protein 0.3–8.0, soluble sugars 0.2–81.0 g kg^−1^ DW		Protein (Bradford 1976); soluble sugar (Sombié et al. 2011)	Ouédraogo et al. 2022
Turnip (*Brassica rapa* subsp. *rapa*)		TSS 70.0–118 g kg^−1^ DW		Not available	Khadivi et al. 2022
				Desulfo GLS: HPLC with a photodiode array detector; intact GLS: accurate mass liquid chromatography–mass spectrometry (De Vos et al. 2007)	Lee et al. 2013a
Tuber crops					
Potato (*Solanum tuberosum*)		Protein 55.0–178, total essential amino acids 15.8–45.1, total non-essential amino acids 31.8–124.8 g kg^−1^ DW		Kjeldahl (Barbano et al. 1990) × 6.25	Rasheed et al. 2025
	K 23.8, P 0.3, Mg 1.2 g kg^−1^ DW; Cu 8.3, Mn 7.2, Zn 27.3, mg kg^−1^ DW; Mineral concentrations highest in cream fleshed potatoes			Perkin Elmer inductively coupled spectrometry (Benton-Jones 1989)	Karan 2023
	Ca 0.2–0.8, K 20.4–32.5, Mg 0.8–1.4, P 3.0–4.8, S 1.1–1.9 g kg^−1^ DW			Inductively Coupled Plasma ‐ Atomic Emission Spectrometry (Havlin and Soltanpour 1980)	Pandey et al. 2023
	Ca 0.1–0.9, K 12.0–30.9, Mg 0.7–1.5, P 2.1–5.0, S 1.1–3.8 g kg^−1^ DW; Cu 2.1–13.3, Fe 17.1–71.0, Mn 7.0–25.2, Mo 0.1–1.2, Zn 9.6–34.8 mg kg^−1^ DW		Dry matter 13.7–27.8%	iCAP 7000 Series ICP-OES, Thermo ScientificTM	Singh et al. 2022c
Yam (*Dioscorea* spp.)	Substantial variability for minerals among Chinese Yam landraces	Substantial variability for carbohydrate, protein, and total amino acids among Chinese Yam landraces		Carbohydrate (GB 28,050 − 2011); protein (GB 5009.5–2016); amino acids (GB 5009.124–2016); minerals: atomic absorption spectrometry (Ca: GB 5009.92–2016; Na and K: GB 5009.91–2017; Mg: GB 5009.241–2017; Mn: GB 5009.242–2017; Fe: GB 5009.90–2016; Zn: GB 5009.14–2017); Inductively coupled plasma mass spectrometry (Cu: GB 5009.13–2017; Se: GB 5009.93–2017) Molybdenum blue spectrophotometry (P: GB 5009.87–2016)	Lin et al. 2025
				Protein (Bradstreet 1965); carbohydrate (FAO 1998); starch (Englyst and Cummings 1988; McCleary et al. 1994, 1997); lipid (Folch et al. 1957); carotenoids (Bona-Lovasz et al. 2013)	Ratsimbazafy et al. 2023
	K 8.3–13.6, Na 0.3–0.9 g kg^−1^ DW	Protein 54.0–103 g kg^−1^ DW		Protein (AOAC 2012); minerals (AOAC 2012); Vitamin C (Omaye et al. 1979) and E (Baker et al. 1980)	Pradhan et al. 2020
	Ca 0.2–0.7, P 1.8–2.5 g kg^−1^ DW; Fe 111.5–286.1, Zn 21.1–62.1 mg kg^−1^ DW		Co 10.6–19.8, Cr 21.0–45.3, Ni 3.0–8.9, Pb 1.1–9.3 mg kg^−1^ DW heavy metals were lower than the tolerable level	Minerals (AOAC 2012; P, James et al., 1995); phytate (Wheeler and Ferrel 1971)	Pradhan et al. 2018

FC, Crude fibre; DW, dry weight; FW, fresh weight; RS, Reducing sugar; TC, Total carbohydrate; TS, Total sugar; TSS, Total soluble solids

**Table 4:** Genetic variation and commentary on secondary compounds (non-nutrient) in garlic, onion, cassava, taro, turnip, potato and yam genetic resources. Units are standardized where applicable, and units not standardized are considered study-specific

Crop	Secondary Compounds and Bioactives	Method	Reference
Bulb crops			
Garlic (*Allium sativum*)	Eight purple garlic cultivars from China exhibited 36 volatile compounds, representing phenols, aldehydes, alcohols, sulphur-containing compounds, hydrocarbons, ethers, ketones, and furans, with diallyl disulfide being the most abundant, followed by 3H-1, 2-dithiole, which together accounted for 25% of the total sulphide content	HS-SPME/GC–MS analysis of all compounds (Jiménez-Amezcua et al. 2025; Wei et al. 2022)	Sun et al. 2025a
	Antioxidant activity 2.0–7.8 mM TE g^−1^ FW, phenolics 33.0–76.2 mg GAE 100 g^−1^ FW	Spectrophotometric analysis of phenolics (Singleton and Rossi 1965) with some modification; DPPH assay for analysis of antioxidant activity (Blois 1958)	Alicandri et al. 2025
	Allicin 2.7–7.3 mg 100 g^−1^ FW, flavonoids 19.0–129.7 mg QE 100 g^−1^ FW, phenolics 28.3–75.0 mg GAE 100 g^−1^ FW, pyruvic acid 9.7–60.9 μMol ml^−1^ FW	TSS (Hand refractometer); Phenol (Singleton et al. 1999); Spectrophotometric analysis of flavonoids (Zhishen et al. 1999); allicin (Singleton et al., 1999); and pyruvic acid (Anthon and Barrett 2003)	Pasupula et al. 2024
	Garlic landraces ‘Aglio di Peschici’ and ‘Aglio Rosso di Monteleone di Puglia’ showed high levels of cations and phenols, and low levels of volatile-(S)-compounds compared to modern cultivars	HPLC analysis of organic acid (Calvo-Gómez et al. 2004; Trani et al. 2016) and phenolics (Pasqualone et al. 2017)	Bonasia et al. 2020
	Phenolics 30.2–60.2 mg GAE 100 g^–1^ FW, pyruvate 3.9–5.2 mmol PV 100 g^−1^ FW	Spectrophotometric analysis of pyruvate (Schwimmer and Weston 1961) and phenolics (Singleton and Rossi 1965)	Barboza et al. 2020
Onion (*Allium cepa*)	Antioxidant activity 0.4–1.4 µmol g^−1^ FW, flavonoids 1.1–2.3 mg g^−1^ FW, phenol 0.7–3.5 mg g^−1^ FW, pyruvic acid 0.2–9.4 µmol g^−1^ FW	Spectrophotometric analysis of pyruvic acid (Schwimmer and Weston 1961), phenolics, and flavonoids (Gupta et al. 2025); FRAP assay for measurement of antioxidant activity (Benzie and Strain 1996)	Gupta et al. 2025
	Flavonoids 0.2–2.6 QE g^−1^, pyruvic acid 0.2–17.2 μM, phenolics 0.04–2.0 GAE g^−1^	Spectrophotometric analysis of pyruvic acid (Anthon and Barret 2003); vitamin C, (Cunnif 2000); phenolics (Zhang et al. 2016a, b), and flavonoids (Kaur and Kapoor 2002)	Tabussam et al. 2022
Root crops			
Carrot (*Daucus carota* subsp. *sativus*)	Anthocyanin 0.5–1799.7 μg 100 g^−1^ DW, phenolics from 69–308.2 μg 100^–1^ g DW; negligible flavonoids	Spectrophotometric analysis of anthocyanin/phenolics (Gras et al. 2016; Mohamedshah et al. 2020; Mazza et al. 1999)	Seth et al. 2025
	Anthocyanin 1.3–119.8 mg 100 g^−1^ DW	Spectrophotometric analysis of nthocyanin (Wu et al. 2018; Zhang et al. 2020a, b); quinic acid (Liu et al. 2022; Yao et al. 2011); and vitamin C (Chen et al. 2023; Li 2000)	Chen et al. 2024
	Colour variation among carrot germplasm is related to diversity in polyphenol and carotenoid content	UV–Vis spectrophotometry and HPLC for analysis of phenolics (Singleton et al. 1999); flavonoids (Chang et al. 2002); proanthocyanins (Sun et al. 1998); and anthocyanins (Awika et al. 2005)	Balakrishna et al. 2024
	Black carrot landrace possessed much greater total anthocyanin and antioxidant capacity than orange carrot landrace	Spectrophotometric and HPLC–DAD analysis of phenolics (Gerardi et al. 2018); anthocyanins (Chandra et al. 2001); and carotenoids (Durante et al. 2016; Koch et al., 2005)	Blando et al. 2021
Cassava (*Manihot esculenta*)	Large variability for hydrogen cyanide content among cassava germplasm accessions	Multiple methods for hydrogen cyanide including 0 to 800 ppm color scale (Egan et al. 1999); modified picrate method (Haque et al., 2002) followed by 1–9 scale	Kanaabi et al. 2024b
	β-carotene varied from 26.1 to 76.7% of the 21 carotenoids observed among landraces; colour variability ranged from pale-to-intense yellow	HPLC and spectrophotometric analysis of carotenoids (Kimura et al. 2005)	Carvalho et al. 2016
Radish (*Raphanus sativus*)	Substantial phytochemical diversity for carotenoids, antioxidant capacity, and total phenol content	UV–Vis Spectrophotometric and spectrophotometric analysis of carotenoids (Braniša et al. 2014); phenolics (Lin et al. 2011); antioxidant capacity (Jelodarian et al. 2012; Keser et al. 2012); and anthocyanin (Gallik 2012)	Tare et al. 2026
	Multi-fold differences among radish germplasm accessions for total phenolics, anthocyanin and antioxidant activities	Spectrophotometric analysis of phenolics (Singleton et al. 1999); anthocyanins (Wrolstad et al. 2005); and antioxidant activity (Benzie and Strain 1996)	Singh et al. 2017
	Glucosinolates 2951–13,528 nmol g^−1^	HPLC analysis of glucosinolates (Bennett et al. 2004; Brown et al. 2003)	Yi et al. 2016
Sweet potato (*Ipomea batatas*)	Anthocyanins: 0–200.3 mg 100 g^−1^ DW, caffeoylquinic acids 0.5–14.2 g kg^−1^ DW, and β-carotene 0–133 mg kg^−1^ DW	HPLC analysis of anthocyanins (Truong et al. 2010); β-carotene (Hosotani and Kitagawa 2003); and caffeoylquinic acid (Zhao et al. 2024)	Zhao et al. 2024
	Purple sweet potatoes high in anthocyanins 55.7–143.4 mg g^−1^ DW, α-carotene 4.8 ± 1.2 mg 100 g ^–1^ DW	Reverse-phase LC and spectrophotometric analysis of anthocyanins (Giusti and Wrolstad 2001); and carotenoids (Barba et al. 2013; Schierle et al. 2004)	Nabot et al. 2024
Taro (*Colocasia esculenta*)	Antioxidant activity 39.2–98.7%, flavonoids 0.3–1.5 mg QE 100 g^−1^ DW, phenolics 4.4–115.2 mg GAE 100 g^−1^ DW	Spectrophotometric analysis of phenols (Singleton et al. 1999); flavonoids (Arvouet-Grand et al. 1994); and antioxidant activity (Sombié et al. 2011)	Ouédraogo et al. 2022
Turnip (*Brassica rapa* subsp. *rapa*)	Landraces* ‘*Cima di rapa dalla testa’ and ‘Cima di rapa antica’ exhibited glucosinolates 741 µmol kg^−1^ FW, phenols 34.7 g 100 g^−1^ FW	Spectrophotometric and chromatographic analysis of Vitamin C (Koh et al. 2012); phenolics (Bonasia et al. 2013); and glucosinolates (Conversa et al. 2016)	Conversa et al. 2020
	Glucosinolates 0.3–0.5 mg g^−1^ DW	LCMS and spectrophotometric analysis of glucosinolates (Wang et al. 2012)	Paul et al. 2019
	13 glucosinolates in leaves and roots, 2-hydroxy-3-butenyl GLS and 2-phenylethyl GLS being in greater quantity in tubers; 24 and 16 hydrolysis products in tubers and leaves, respectively	HPLC–DAD and GC–MS analysis of Desulfo glucosinolates (Wiesner et al. 2013; Witzel et al. 2015)	Klopsch et al. 2017
	11 glucosinolates in roots	HPLC and LC–MS analysis of glucosinolates (De Vos et al. 2007; Kliebenstein et al. 2001)	Lee et al. 2013a
Tuber crops			
Potato (*Solanum tuberosum*)	Coloured accessions exhibited higher levels of total phenolics, flavonoids, and antioxidant activities than white accessions	Spectrophotometric analysis of phenolics (Ainsworth and Gillespie 2007); flavonoids (Xu et al. 2016); and antioxidant activity (Brand-Williams et al. 1995)	Rasheed et al. 2024
	Purple-blackish tetraploid tubers had the highest phenolics level 2062.4 ± 547.4 mg GAE 100 g FW, while diploid pink-red tubers had the highest antioxidant level (DDPH: 14,967.1 ± 4687.8 μmol TE; FRAP: 2208.6 ± 797.4 mg AAE)	HPLC and spectrophotometric analysis of phenolics (Singleton and Rossi 1965); antioxidant activity (Brand-Williams et al. 1995; Benzie and Strain 1996); and vitamin C (Kelebek et al. 2009)	Berdugo-Cely et al. 2023
Yam	*D. praehensilis* (semi-domesticated) and purple *D. alata* species (domesticated) exhibited highest antioxidant activity	Chromatographic and spectrophotometric analysis of β-carotene content (Barba et al. 2006); tannins (Burns 1971; Price et al. 1978); flavonoids (Harborne and Williams 1973; Jagadish et al. 2009); polyphenols (Waterman and Mole 1994); and antioxidant activity (Molyneux 2004)	Mondo et al. 2025
	Diosgenin 1.1–13.3 g 100 g^−1^ DW	HPLC analysis of diosgenin (Heping et al. 2008; Huang et al. 2003)	Sun et al. 2024
	Wild accession from Henan region in China had the highest concentrations of diosgenin, vitamins, and polysaccharides, with some cultivars suitable for food and medicines	UPLC analysis of diosgenin; Zeng et al. 2021; Tan et al. 2020)	Zeng et al. 2021
	Flavonoids 60–160 mg CE 100 g^−1^ DW, monomeric anthocyanins 10–90 mg CE 100 g^−1^ DW, phenolics 100–385 mg GAE 100 g^−1^ DW	Spectrophotometric analysis of flavonoids (Yang et al. 2004); phenol (Liu and Lin 2009); and anthocyanins (Boyles and Wrolstad 1993)	Srivichai and Hongsprabhas 2020

HS-SPME/GC-MS: gas chromatography mass spectrometry with solidphase microextraction; TE: Trolox equivalent; HPLC: high performance liquid chromatography; LC-MS: liquidchromatography – mass spectrometry; HPLC-DAD: high performance liquid chromatography with diode arraydetection; GAE: Gallic acid equivalent; UV-Vis: ultra violet visible spectroscopy; FW: Fresh weight; DW: Dryweight; QE: Quercetin equivalent; PV: Pyruvate value; GLS: Glucosinolate; DDPH: 2,2-diphenyl-1-picrylhydrazyl; FRAP: Ferric reducing antioxidant power; AAE: Ascorbic acid equivalent; CE: Catechinequivalent

Red garlic landraces, “Aglio Rosso di Proceno” and “Aglio Rosso di Castelliri” from Lazio, Italy, are prized for their unique organoleptic qualities and cultural importance (Alicandri et al. 2025). ‘Aglio di Peschici’ and ‘Aglio Rosso di Monteleone di Puglia’ from Foggia, Italy, are known for their dense minerals and phenols (Bonasia et al. 2020). Purple garlic accessions from China exhibited abundant volatile organic compounds, with diallyl disulfide and 3H-1, 2-dithiole, being the most abundant (Sun et al. 2025b). ‘Acquaviva Red Onion landrace (ARO)’ has been in cultivation for centuries by farmers from Bari province, southern Italy. This large, red-coloured onion bulbs, harvested from the field, are consumed locally fresh. It is a prized landrace, well known for its sweetness, protected by the European Union under ‘protected geographical indication (PGI) and protected designation of origin (POD)’ to limit adulterations and improve the income of smallholders. Its well-defined genepool is distinct from the ‘Tropea’ and ‘Montoro’ landraces, with which it is often adulterated (Ricciardi et al. 2020). Colour variation in root storage organs also impacted polyphenols and carotenoids, for example, black carrot germplasm was rich in phenolics, flavonoids, proanthocyanins, and tannins, red carrot in anthocyanins, and orange carrot in carotenoids (Balakrishna et al. 2024). A leading hybrid carrot cultivar, ‘Purple Sun’, contains 28 bioactive compounds, with hydroxycinnamic acid derivatives and anthocyanins predominant, and is a promising candidate for pharmaceutical and/or nutraceutical products (Maresca et al. 2024). Puglia farmers of Italy selected nutritionally enhanced variants from turnip landraces,* ‘Cima di rapa dalla testa*’ and ‘*Cima di rapa antica*’ (Conversa et al. 2020). A few yam landraces from China are specifically grown for their unique organoleptic properties and classified as ‘geographical Indications for Agricultural Products’ (Lin et al. 2025). Numerous yam species show high medicinal significance due to their high antioxidant capacity or high diosgenin, vitamins, and polysaccharides (Cheng et al. 2021; Mondo et al. 2025; Ratsimbazafy et al. 2023; Sun et al. 2024; Zeng et al. 2021).

## Assessing population structure and diversity in RTB crops germplasm pool

Understanding diversity and genetic relationships among and within crop germplasm is a prerequisite in applied breeding. Genetically diverse accessions contribute significantly to trait genetics and to selection in crop improvement programs. Genetic diversity refers to variation among alleles of a given gene, which may be examined at the nucleotide level in the DNA sequence. Both classical and DNA tools are deployed to assess diversity at morphological and molecular levels, which can be expressed in dendrograms, percentage polymorphic loci and genetic distance. Molecular marker-based genetic estimates are more reliable and remain unaffected by the environment and genotype-by-environment (GEI) interactions. Genetically diverse accessions are deployed as a resource to dissect trait genetics and later develop improved germplasm with agronomically beneficial traits. The strategic integration of new genetic diversity is the key to success in plant breeding, especially when dealing with wide crosses, while preserving co-adapted gene complexes, highly specialised for specific consumer preferences or market needs (Swaroop et al. 2021). Genetically diverse accessions are also useful in formulating association mapping panels or specialized populations to derive recombinant inbred lines (RILs) (Samal and Martin 2017) or double haploid lines (DHLs, Kasha and Maluszynski 2003) to efficiently achieve homozygosity without the long process of multiple rounds of self-pollination) for dissecting trait genetics or deriving improved germplasm.

Morphological descriptors and/or DNA-based markers are routinely used to assess population structure and diversity in crop germplasm. Phenotypic characterisation is equally important in defining the plant populations, as it forms the basis for selecting accessions with beneficial agronomic traits. Numerous germplasms with superior morphological features, rich in nutritional quality and bioactive compounds and differing in RTB storage organs shape and colour have been identified (Bessa de Carvalho et al. 2022; Chen et al. 2022; Galvao et al. 2021; Ochar et al. 2026; Petropoulos et al. 2018a, b; Ricciardi et al. 2020; Singh et al. 2022b; Sönmez et al. 2025; Tabussam et al. 2022; Wang et al. 2024a). However, diversity based on morphological descriptor is less reliable due to strong location effects and significant GEI. Hence, marker-based (preferably using SNPs/indels/SVs) genetic distance and clustering patterns are more reliable to identify genetically diverse accessions for use in crop improvement programs. Following this, genetically diverse accessions with agronomically beneficial traits are reported in RTB crops (Table 5). A few reports also revealed either consistent (Wu et al. 2025b) or inconsistent clustering patterns between morphological- and molecular-distance-based matrices due to non-overlapping information between the two data types (Darkwa et al. 2020). However, the combined analysis of data maximizes a comprehensive estimate of diversity in the evaluated materials.

**Table 5:** Population structure and diversity by polymorphic DNA markers in major root, tuber, and bulb crops

No. germplasm	DNA markers	Summary of population structure and diversity	Reference
Garlic (*Allium sativum*)			
35	13 simple sequence repeats (SSRs)	Four distinct genetic clusters, with the two Lazio landraces forming separate groups, indicating their genetic distinctiveness	Alicandri et al. 2025
606	1,100,258 single nucleotide polymorphisms (SNPs)	Five subpopulations	Jia et al. 2023
Onion (*Allium cepa*)			
35 (white onions)	23 SSRs and intro length polymorphisms (ILPs)	Two distinct clusters; high total soluble solid content (HTSS) of white onion is highly preferred	Singh et al. 2021
Carrot (*Daucus carota* subsp. *sativus*)			
98	48 of 271 Insertions/Deletions (InDels)	High polymorphism (84.21%) across eastern and western gene pools, with intense selection and domestication, noted in western genepools	Mudihal et al. 2025
52	176 MITEs (*DcSto*s-ILP genotyping system)	2-subpopulations, differentiated eastern and western accessions; 3 subpopulations among Indian germplasm, distinct from eastern accessions	Hadagali et al. 2024
144 (five root color types)	80	Three subpopulations, representing broad-based populations	Chaitra et al. 2021
Radish (*Raphanus sativus*)			
83	796,294 SNPs	3 sub-populations	Huh et al. 2023
217	24 SVs mapped on 9 chr	3 sub-populations based on geographical origin	Li et al. 2023
510	2,624 SNPs	Four main groups, representing 4 geographical regions	Kobayashi et al. 2020
Sugar beet (*Beta vulgaris*)			
94	4,609 SNPs and 6,950 Diversity Array Technology (DArT) markers	Three and two distinct clusters for SNP and DArT data, respectively	Bahjat et al. 2025
238 (diverse ecotypes, incl table beet)	10,237 SNPs (full set), 9,847 SNPs (table beet)	Distinct clustering pattern among 226 accessions, while 165 table beet accessions showed no clustering pattern	Pelikan and Goldman 2024
Sweet potato (*Ipomea batatas*)			
50	10 SSRs	Two major clusters, with orange flesh color genotypes had higher ß-carotene than white, cream, or yellow flesh color	Merga et al. 2025
56	12 SSRs	Grouped on-farm accessions into two clusters, and genebank accessions into 3 distinct clusters	Guimarães et al. 2025
23	Morpho-physio-nutritional-yield traits	Four distinct clusters, with cluster II representing high-yielding and cluster I moderately carotene-enriched cultivars	Modak et al. 2025
604	102,870 SNPs	6 unique clusters across seven broad geographic regions	Slonecki et al. 2023
66	39,424	5 subpopulations	Nie et al. 2023
5979	20 SSRs	Four ancestral populations, with many of the accessions having lower levels of gene flow from the other population	Anglin et al., 2021
239	214 SSRs	Two subpopulations	Zhang et al. 2016a, b
Taro (*Colocasia esculenta*)			
72	27 pairs of PARMS-SNP markers	6 sub-populations, with similar clustering patterns between phenotype- and marker-based grouping	Wu et al. 2025b
490	3047 DArT-SNPs	Higher genetic diversity among two genepools; geneflow intermixing, 4 clusters, one comprising of Dasheen and three comprising Eddoe genepool; phenotype-based clustering resulted in 3 clusters	Oladimeji et al. 2024
271	10,391 SNPs (DArTseq-based SNP)	Five admixture subgroups, with Vanuatu taro accessions exhibited more genetic diversity than Nigerian taro accessions	Fufa et al. 2022
234	132,869 SNPs (SLAF-seq)	Eight sub-populations, with higher variation among groups than within groups	Wang et al. 2020b
Turnip (*Brassica rapa* subsp. *rapa*)			
56	280 SNPs	Two subpopulations, with admixture, corresponded with geographical origin, Europe or Asia	Zhang et al. 2014
Potato (*Solanum tuberosum*)			
104	1,101,368 SNPs and 141,656 INDELS	Two subpopulations, with colored potatoes showed higher levels of phenolics, flavonoids, and antioxidant activities than the white potatoes	Rasheed et al. 2024
110 Korean and 393 global clones	SolCAP 8303 SNPs Infinium array	Korean clones represented by 3 subpopulations, while global potato clones represented by country/region-specific clustering patterns	Jo et al. 2022
214	10,106 SNPs (22 K V3 potato array)	Three subpopulations among tetraploid clones, based on market classes; heterozygosity varied by SNP, with an overall average of 0.59	Pandey et al. 2021
Yam (*Dioscorea* spp.)			
173	1.6 m SNPs	3 sub-populations	Agre et al. 2025
94	DArT-SNPs	2 and 3 clusters based on phenotypic and genotyped-based clustering, respectively, in aerial yam species (*Dioscorea bulbifera*) accessions	Ekaette et al. 2024
173	136,429 SNPs	Inconsistent clustering pattern between morphological and molecular-based distance matrices due to the non-overlapping information between the two data types	Darkwa et al. 2020
44	6457 SNPs	Four subpopulations in *D. dumetorum*, with a western/north-western, a western, and south-western populations, suggesting altitude and farmers-consumers preference drive differential adaptation and separation of these populations; *D. dumetorum* accessions from south-western region with highest genetic variability	Siadjeu et al. 2018

MITEs, Miniature inverted-repeat transposable elements; PARMS-SNP: Penta-primer amplification refractory mutation system single-nucleotide polymorphisms

## Functionally characterized genes associated with storage root growth and thickening, nutrient and non-nutrient traits

*Storage root thickening in radish:* Tap root thickening is an important commercial trait in radish. A total of 65 Aux/IAA genes were identified in the radish genome, most of which exhibited a differential expression pattern in different root tissues. Six root-specific genes were highly expressed in the root cortex, cambium, xylem, and root tip in radish. The root cortex is the outer layer between the epidermis and vascular tissues. Root cambium is a layer of actively dividing cells, which originates from undifferentiated cells and produces secondary xylem and secondary phloem as the root grows. Xylem is a vascular tissue in plants that primarily transports water and minerals from the roots to the rest of the plant organs. The silencing of *RsIAA33* inhibited reproductive growth to promote taproot thickening and development in radish roots (Xie et al. 2021).

*Sugar beet taproot growth:* The fleshy taproot is an important harvesting organ, and the focus of traditional breeding for increased sugar content and yield. The primary growth stages of taproot development include pre-storage, transition, secondary growth, and sucrose accumulation, each characterized by the distinct metabolic and phytohormonal signatures (Jammer et al. 2020). The growth-promoting steroid hormone, brassinosteroids, regulates several physiological functions in plants, and BRASSINAZOLE-RESISTANT TFs are key elements of brassinolide signal transduction (Chakraborty et al. 2025). Transgenic sugar beet overexpressing *BvBZR1* exhibited a higher taproot diameter and significantly enhanced the accumulation of sucrose and soluble sugars as compared to wild type (WT). *BvBZR1* regulates the expression of cell wall and sucrose metabolism genes, improves corresponding enzyme activity, and promotes the development of each cambium ring and parenchyma cell, thereby promoting the growth and development of sugar beet taproots (Li et al. 2025a).

*Nutrient traits:* Sweet potato starch is in high demand as a food and industrial product for various end uses. A natural allelic variation in *IbNAC22* significantly influences starch biosynthesis and properties in sweet potato. *IbNAC22* is highly expressed in storage roots and starch-rich sweet potato accessions. Transgenic sweet potato overexpressing *IbNAC22* significantly improved starch and amylose contents, granule size, and gelatinization temperature, decreased starch crystallinity, whereas *IbNAC22* knockdown had the opposite effects. *IbNAC22* activates the expression of a gene associated with amylose biosynthesis, *IbGBSSI*, but suppresses the expression of *IbSBEI*, associated with amylopectin biosynthesis (Fan et al. 2025). Sucrose transporters are essential proteins that allocate assimilates from source tissues, such as leaves, to sink organs, such as stems, roots, and seeds, and play a crucial role in carbon allocation and biomass accumulation in most crops, including yams (Julius et al. 2017). Eight *SUTs* are involved in sucrose binding and transport in yams. Expression profiling revealed that most are involved in sucrose binding and transport, indicating their roles in sucrose unloading and cell expansion. Functional validation of *DrSUT1* in *Arabidopsis* confirmed its involvement in sucrose transport, partitioning, and tuber development in yams (Li et al. 2025b).

*Non-nutrient traits:* Garlic allicin is known for its powerful antimicrobial, antioxidant, and cardiovascular benefits (Thakur et al. 2024). Transgenic garlic callus overexpressing *AsWRKY9* significantly increased alliin content by activating the allicin biosynthesis pathway (Wu et al. 2025a). Root vegetables rich in anthocyanins and/or carotenoids are popular among consumers for their attractive colour and high nutritional value. Carrot and radish were extensively investigated to unlock the genetic and molecular basis of anthocyanin accumulation and biosynthesis (Table 6). The functionally characterized genes associated with anthocyanin biosynthesis and accumulation include *DcMYB6*, *DcMYB7*, *DcMYB113, DcGST1*, *DcMYB113_NB,* and *bHLH DcEGL1_NB*, *AmRosea1,* and *AmDelila* in carrot and *LINC15957*, *RsTTG1*, and *RsPAP2* in radish. *DcLCYB1* and *DcLCYE* knockouts enhance lycopene accumulation, while their overexpression reduces it. Lycopene is a potent antioxidant found in red carrots. The overexpressing sweet potato transgene (*IbbHLH25* or *IbOr-Ins*) exhibited increased intensity of anthocyanin and carotenoids, and vice versa when a transgene knockout or its function is silenced (Wei et al. 2025; Park et al. 2015). However, functionally characterized genes or DNA markers should be further validated for polymorphisms in target breeding populations, including for epistasis and environment interactions, to assess stability and transferability of a gene or marker in a given breeding population. Complex traits, such as seed yield or climate resilience, are often controlled by networks of interacting genes with small effects. Epistasis and genotype-by-environment interactions may nullify the effect of genes or DNA markers. Hence, the target gene or marker might fail to confer the expected phenotype if complementary modifier genes are missing in the recipient population (Hawley and Gilliland 2006). Additionally, if a beneficial allele is already fixed in improved germplasm, a marker for it may not improve the population. Likewise, crossing over in successive generations can break linkage between the marker (flanking markers rather than causative mutation) and the functional allele.

**Table 6:** Functionally characterized genes associated with non-nutrient traits in garlic, carrot, radish, and sweet potato

Gene	Functional characterization	Evidence	Reference
Garlic (*Allium sativum*)			
*AsWRKY9*	Overexpressing transgenic garlic callus significantly increased alliin content by activating alliin biosynthesis	Transgene	Wu et al. 2025a
	Onion (*Allium cepa*)		
*AcFT1*, *AcFT4*	Antagonistic genes associated with bulb formation: *AcFT1* promotes bulb formation, while *AcFT4* prevents *AcFT1* upregulation and inhibits bulbing	Transgene	Lee et al. 2013b
Carrot (*Daucus carota* subsp. *sativus*)			
*DcCYP97C1*	Rate-limiting enzyme for lutein biosynthesis and important for taproot color formation	Gene editing/expression pattern	Deng et al. 2025
*DcLCYB1* and *DcLCYE*	Overexpression reduces lycopene levels, while knockout leads to elevated lycopene accumulation and red phenotype	Overexpression/gene knockout	Li et al. 2025d
*DcGST1*	Knocked out *DcGST1* in the purple carrot cultivar ‘Deep Purple’ resulted in carrots with orange taproots; however, noted cultivar-specific anthocyanin expression	Knockout (gene editing)	Duan et al. 2024
*DcMYB113_NB* and *bHLH DcEGL1_NB*	Simultaneous expression, under the control of a constitutive promoter, turned an orange carrot into an anthocyanin-rich carrot	Transgene	Sharma et al., 2021
*AmRosea1* and *AmDelila* (snapdragon)	Transgenic orange carrot expressing *AmRosea1* and *AmDelila* turned purple due to significant accumulation of anthocyanins in leaves, stems and taproots;	Transgene	Sharma et al. 2020
*DcMYB113*	Transgenic ‘Kurodagosun’carrot carrying *DcMYB113* driven by the CaMV 35S promoter had a purple root and petiole, while transgenic ‘Kurodagosun’ expressing *DcMYB113* driven by its own promoter had purple root and nonpurple petiole, suggesting root-specific expression	Transgene	Xu et al. 2020
*DcMYB7*	Overexpression in orange carrot increased anthocyanin, while knockout resulted in carrot with yellow roots	Expression pattern/knockout	Xu et al. 2019
*DcMYB6*	Overexpression in *Arabidopsis* enhanced anthocyanin accumulation in vegetative and reproductive tissues	Transgene	Xu et al. 2017
Cassava (*Manihot esculenta*)			
	An allelic polymorphism in one of the two *phytoene synthase* (*PSY*) genes enhances flux of carbon through carotenogenesis, leading to increased ß-carotene accumulation in storage roots	Transgene	Welsch et al. 2010
Radish (*Raphanus sativus*)			
*LINC15957*	Overexpressing transgenic radish showed significantly enhanced anthocyanin accumulation in radish leaves, associated with significantly enhanced expression of structural genes, while anthocyanin accumulation and structural gene expression levels significantly reduced in silenced *LINC15957* flesh	Overexpression/ gene silencing	Tan et al. 2023
*RsTTG1*	Heterologous expression in the *Arabidopsis* mutant (*ttg1-22*) background restored the accumulation of anthocyanin and proanthocyanidin synthesis, while the transient expression of *RsTTG1* in tobacco leaves exhibited an increase in anthocyanin accumulation	Heterologous expression	Lim et al. 2022
*RsPAP2*	Overexpression resulted in anthocyanin accumulation in *Arabidopsis* and radish, accompanied by the upregulation of several anthocyanin-related structural genes	Transgene	Fan et al. 2020
Sweet potato (*Ipomoea batatas*)			
*IbbHLH25*	Overexpression increased β-carotene and β-cryptoxanthin carotenoids increased by 21.2-fold to 55.7-fold and 4.6-fold-9.5-fold, respectively; gene silencing had opposite effects	Overexpression/ silencing	Wei et al. 2025
*IbOr-Ins*	Transgenic plants overexpressing *IbOr-Ins* under the control of CaMV 35S promoter in an anthocyanin-rich, purple-fleshed sweet potato cultivar exhibited increased carotenoid levels (up to 7-fold) in their storage roots compared to WT plants	Transgene	Park et al. 2015
Potato (*Solanum tuberosum*)			
*PTST2b* and *MRC*	Target genes for altering starch granule size/shape in tubers, with no adverse effect on starch content and tuber yield	Transgene; overexpression/ silencing	Hochmuth et al. 2025
Yam (*Dioscorea rotundata*)			
*DrSUT1*	Involved in sucrose transport in sink tissues	Transgene	Li et al. 2025b

## Putative (or candidate) genes and genomic regions (or quantitative trait loci [QTL]) associated with bulb/root morphology and thickening, nutrient and non-nutrient traits

Numerous QTLs and putative genes associated with RTB storage organ morphology, shape and thickness (Table 7), nutrient (Table 8), and non-nutrient (Table 9) traits suggest the complex genetic control of these edible organs. The examples include multiple QTLs associated with bulb size, shape and weight in onion (Sekin et al. 2025); root shape and length-to-width root ratio in carrot (Vega et al. 2024); root morphology and thickening in radish (Wang et al. 2020a, b) and sweet potato (Bowers et al. 2024; Cai et al. 2022); early storage root bulking (i.e., the process where the storage roots begin to grow and mature earlier than the typical growth period) in cassava (Abah et al. 2024); and tuber induction and morphology in potato (Hara-Skrzypiec et al. 2018; Li et al. 2025e) and yam (Qiao et al. 2025).

**Table 7:** Candidate (putative) genes associated with bulb size, shape and weight in onion; storage root size and shape in carrot, radish, sweet potato, and turnip; early storage root bulking in cassava and sweet potato; skin roughness and coarseness in sugar beet; tuber development in potato and yam; and oxidative browning in garlic, sweet potato and yam

Crop	Summary of genomic regions and candidate genes	Evidence	Reference
Bulb crops			
Garlic (*Allium sativum*)	234 SNPs associated with the storability, located in or near 401 genes; 3 hub and 21 putative genes associated with storability	GWAS/transcriptome	Zhu et al. 2024
Onion (*Allium cepa*)	QTL for bulb shape, shape and weight detected; SNPs mapped on chr 8 significantly associated with bulb weight	GWAS/QTL analysis across genetic backgrounds/environments	Sekin et al. 2025
	Two QTL associated with bulbing, predominantly additive effects; One QTL associated with bulb-shape index	QTL analysis	Havey 2024
Root crops			
Carrot (*Daucus carota* subsp. *sativus*)	Root shape: 2 major QTL mapped on chr 2 and 6; length-to-width root ratio: 4 QTL; *OFP-TRM* and *IQD* as candidate genes within QTL region	QTL analysis	Vega et al. 2024
	Multiple QTL for four market classes—length, aspect ratio, maximum width, and root fill	GWAS	Brainard et al. 2021b
Cassava (*Manihot esculenta*)	45 SNPs and 199 putative genes significantly associated with early storage root bulking; 4 pleiotropic SNPs with significant effect on starch and dry matter contents, dry root yield, and bulking and formation index	GWAS	Abah et al. 2024
Radish (*Raphanus sativus*)	miR393a-*bHLH77*, miR167c-*ARF8*, and miR5658-*APL* regulate taproot thickening	Expression pattern	Xie et al. 2020
	*RsOFP2.3* regulates organ shape; *RsOFP2.3* suppresses root elongation	Expression pattern	Wang et al. 2020a, b
Sugar beet (*Beta vulgaris* subsp. *vulgaris*)	*DFAX2* and *P5CS* regulate skin roughness; *FRO5*, *GL24*, and *PPR91* regulate root and sugar yields, and the pleiotropic gene *POLX* regulates flesh coarseness and sugar yield	GWAS	Wang et al. 2023
Sweet potato (*Ipomoea batatas*)	28 SNPs, mapped on 21 genetic loci, associated with root traits: dry matter, 4 loci; flesh color, 5 loci; flesh hue angle, 3 loci; skin color and skin hue angle, 9 loci; several candidate genes located within 100 kb from the SNPs affecting storage roots	GWAS	Bowers et al. 2024
	Trihelix TF is associated with tuberous root enlargement	Expression pattern	Cai et al. 2022
	Several candidate genes involved in storage root expansion: *SBEI*, *ARF6*, *NF-YB3* and *NF-YB10* significantly up-regulated	Expression pattern	Song et al. 2022
	IbNAC083 TF regulates the initiation of tuberization	Expression pattern	He et al. 2021
	34 and 7 unique SNPs significantly associated with continuous storage root formation and bulking (CSRFAB) and discontinuous storage root formation and bulking (DCSRFAB); 12 and 7 candidate genes annotated for CSRFAB and DCSRFAB traits, respectively	GWAS	Bararyenya et al. 2020
Turnip (*Brassica rapa* subsp. *rapa*)	2 major QTL, *FR1.1* and *FR7*.1, with the latter narrowed down to a 220 kb region containing 47 putative genes; *Bra003652* (homolog of *AT1G78240*), a candidate gene for swollen root	QTL analysis	Wu et al. 2021
Tuber crops			
Potato (*Solanum tuberosum)*	Unlocked regulatory network controlling tuberous signal transmission from leaves and stems to tubers	Expression pattern	Hu et al. 2025
	28 candidate genes, enriched in carbohydrate metabolism pathways, were consistently involved in tuber induction and formation	Expression pattern	Li et al. 2025e
	21 putative genes associated with anti-browning properties in biparental crosses;	QTL analysis	Wang et al. 2024e
	4–7 QTL associated with tuber shape, tuber shape regularity, eye depth, and tuber weight; a major QTL (PVE 76.8%) on Chr 3 and a minor QTL on Chr 2 for tuber flesh color	QTL analysis	Hara-Skrzypiec et al. 2018
Yam (*Dioscorea* spp.)	14 SNPs and 32 putative genes associated with storage root dry matter and enzymatic browning	GWAS	Ouattara et al. 2026
	*DrRALF1*, *DrRALF2*, *DrRALF3*, *DrRALF4*, *DrRALF5*, and *DrRALF6* associated with tuber expansion	Expression pattern	Qiao et al. 2025
	34 QTL, mapped on 8 chr, corresponded to five of each to tuber size, shape regularity, tubercular roots, skin texture, tuber flesh oxidation, six for oxidation ratio and three for flesh color	QTL analysis	Ehounou et al. 2022
	14 and 4 SNPs significantly associated with tuber flesh (white) color and oxidative browning, respectively, and 24 putative genes; favorable alleles for desired traits, flesh (white) color and no oxidative browning	GWAS	Dossa et al. 2022
	A large number of DEGs, including *CDPK*, *CaM*, *CDL*, *SAUR*, *DELLA*, *SuSy*, and *expansin*, as well as TF genes (*MADS*, *bHLH*, and *GRAS*) involved in tuber expansion; additionally, miRNA160, miRNA396, miRNA535 and miRNA5021 regulate cell division and differentiation during tuber expansion stage	Expression pattern	Zhou et al. 2020
	SNPs significantly associated with tuber dry matter content on chr 6 and 19 and oxidative browning on chr 5	GWAS	Gatarira et al. 2020

**Table 8:** Genomic regions and putative (candidate) genes associated with storage biomass, pungency, and nutrient traits in onion, cassava, sugar beet, sweet potato, taro, potato and yam

Crop	Summary of genomic regions and candidate genes	Evidence	Reference
Onion (*Allium cepa*)	QTL mapped on chr 3 and 5 associated with pungent flavor and bulb soluble solids	QTL analysis	McCallum et al. 2007
	4 QTL associated with sucrose (*qSC4.1*), glucose (*qGC5.1*), fructose (*qFC5.1*), and total sugar content (*qTSC5.1*), with 6.07–11.47% PVE	QTL analysis	Lee et al. 2021
Cassava (*Manihot esculenta*)	9 SNPs, mapped on 7 Chr., significantly associated with peel loss, gari yield, color parameters for gari and eba, bulk density, swelling index, and eba textural properties	GWAS	Aghogho et al. 2024
	80 SNPs and candidate genes co-located with peak SNPs mapped on Chr 1, 4, 5, 6, 10, 13, 17 and 18, significantly associated with root mealiness, fibre, adhesiveness, taste, aroma, color and firmness	GWAS	Uchendu et al. 2021
Sugar beet (*Beta vulgaris* subsp. *vulgaris*)	An unknown sucrose transporter on chr 6 showed reduced nucleotide diversity in sugar beet accessions compared to other sugar beet types with low sucrose content	Whole genome sequencing	Dhiman et al. 2025
	*UDP-glucose 4 epimerase*, *hydroxyprolineO-arabinosyltransferase*, and *probablepectinesterase* regulate and balance the synthesis of root storage biomass and sugar, with the first two significantly showing higher expression in high biomass accessions, while the latter expression was significantly higher in high sugar accessions	GWAS	Wang et al. 2024a
	A QTL mapped on chr 2, 332 kb upstream of a candidate gene (*Bevul.2G176300*), involved in sucrose biosynthesis and TDS; another QTL on chr 7 differentiated table beet accessions for sweetness	GWAS	Pelikan and Goldman 2024
Sweet potato (*Ipomoea batatas*)	3 SNPs (Iba_chr01a_17621178, Iba_chr10a_773882, Iba_chr12a_38616338) and 4 putative genes (*IbANT1*, *IbCYP86B1*, *IbSCR3*, *IbFER*) significantly upregulated in high-fibre genotypes	GWAS	Kim et al. 2025
	23 SNPs and 37 candidate genes encompassing these significant SNPs associated with sugar composition traits (fructose, glucose, and total sugars), total starch, amylose content, and total carotenoids	GWAS	Nie et al. 2023
Taro (*Colocasia esculenta*)	11,203 mRNAs, 245 CircRNAs, and 299 miRNAs detected at six corm developmental stages; 139 DEGs associated with 24 starch and sucrose metabolism enzymes; *GBSS*, *AGPase*, *UGPase*, *SP*, *SSS*, *βFRUCT* and *SuSy* encoding key enzymes were highly expressed at 4 months of corm development	Expression pattern	Dong et al. 2021
Potato (*Solanum tuberosum*)	74 loci, mapped across all 12 chr, associated with peak viscosity, setback, hardness, and gumminess, with some loci having pleiotropic effects on phosphorus content, pasting and textural characteristics	GWAS	Ahmad et al. 2025
	53 QTL related to fructose, glucose, and sucrose contents; *StTST2*, closely associated with glucose content, directly interacted with *SUT2*	GWAS	Gong et al. 2025
	A total of 119 genes (81 involved in starch metabolism and 38 in SGA biosynthesis and abundant allelic variants were detected in the 6 autotetraploid potato genomes	Starch metabolism and steroidal glycoalkaloid (SGA) biosynthesis pathways	Li et al. 2024a
	Six candidate genes for starch content and SNP-CAPS markers, SNP-CAPS markers (chr2-CAPS6 and chr2-CAPS21), developed and validated in F_2_ and 24 tetraploid potato cultivars	QTL analysis	Li et al. 2021
	Several gene clusters (0.5–4 Mbp width) involved in tuber yield and starch content; antagonistic SNP alleles associated with tuber yield and starch content; 89 SNP alleles had synergistic effects on tuber yield, starch content and starch yield	GWAS	Schönhals et al. 2017
Yam (*Dioscorea* spp.)	*SUT* family genes, highly expressed in sink tissues, are associated with sucrose partitioning and tuber development	GWAS involving yam *SUT* genes	Li et al. 2025b
	SWEET-mediated sucrose transport *DrSWEET6* and *DrSWEET12* are predominantly expressed in tubers and involved in sucrose unloading, cell expansion, and biomass accumulation	GWAS involving *SWEET* genes	Li et al. 2025c
	Six QTL each for dry matter, sugar, and protein, and 7 QTL for starch content, with many independent of genetic background	QTL analysis/diversity panel	Arnau et al. 2024
	Unlocked 3 metabolic pathways associated with quality of boiled yam: pentose and glucuronate interconversions, starch and sucrose metabolism, and flavonoid biosynthesis; 22 genes linked to texture, starch content, and color	Population genomics/candidate gene association analysis	Mota et al. 2024

GWAS: Genome-wide association studies; TDS: Total dissolved solids

**Table 9:** Genomic regions and putative (candidate) genes associated with non-nutrient (flavonoids, carotenoids, and diosgenin) traits in onion, carrot, cassava, radish, sugar beet, sweet potato, turnip, potato, and yam

Crop	Summary of genomic regions and candidate genes	Evidence	Reference
Onion (*Allium cepa*)	One major QTL, *qAS7.1* (*DFR* gene), for anthocyanin synthesis (PVE 87%) and two significant QTL, *qAC4.1* and *qAC4.2*, for anthocyanin content, 19% and 26% PVE	QTL analysis	Choi et al. 2020
Carrot (*Daucus carota* subsp. *sativus*)	3 genomic regions associated with carotenoid biosynthesis, and a novel locus with a candidate gene encoding phytoene synthase	GWAS	Rolling et al. 2025
	DcRPGE1 ^W^–DcAPRR2 interaction prevents DcAPRR2 activation of its target carotenoid biosynthetic genes (*DcPSY1*, *DcPSY2*, *DcLCYE*) reduces carotenoid biosynthesis	Expression pattern/loss of function mutation	Wang et al. 2024d
	The recessive alleles of 3 genes, at the *REC*, *Or* and *Y2* QTL, regulate high α- and β-carotene in the orange phenotype	GWAS	Coe et al. 2023
	*DcMYB7*, *DcMYB113*, and a MADS-box (DCAR_010757) as candidate genes conditioning root outer phloem anthocyanin pigmentation (ROPAP), while *DcMYB7* and MADS-box regulate inner phloem anthocyanin pigmentation (IPAP)	QTL analysis/expression pattern	Bannoud et al. 2021
	*DcSCPL1*, regulates anthocyanin acylation, but is co-expressed with DcMYB7, activating anthocyanin biosynthesis	Expression pattern	Curaba et al. 2020
	A QTL on chr 7 overlapped with the previously identified β-carotene QTL, *Y2*	QTL analysis/expression pattern	Ellison et al. 2017
	*CA4H1*, *CA4H2*, *4CL1*, *4CL2*, *CHI1*, and *F3′H1* associated with anthocyanin production in purple carrots	Expression pattern	Xu et al. 2014
Cassava (*Manihot esculenta*)	11 of 17 pleiotropic loci inversely affected total carotenoid and dry matter, with significantly polygenic covariance between total carotenoid and dry matter remaining unexplained	GWAS	Villwock et al. 2025
	A mutation (*NCED_3.1*), known to influence β-carotene content, in *PSY* transcripts of deep yellow but not of pale-yellow accessions	Expression pattern	Olayide et al. 2023
	18,218 significant marker-metabolite associations and 12 candidate genes associated with variation in metabolites	mGWAS	Ding et al. 2023
	A coordinated regulation of 16 metabolites and 11 co-expression genes, and two TFs genes (*MeMYB5*, *MeMYB42*) associated with anthocyanin biosynthesis	Expression pattern	Fu et al. 2022
Radish (*Raphanus sativus*)	*RST1*, a major locus (mapped on chr 7) involved in skin color, mediated by interacting genes *RsbHLH* and *RsWD*, and latent genes *RsDFR* and *RsANS* of RST1 in the anthocyanin synthesis pathway	QTL analysis	Liu et al. 2025
	*RsMYB1* and *RsMYB2* regulate anthocyanin accumulation; functional divergence of paralogous *RsMYBs*, with *RsMYB1* regulating red flesh in ‘Xinlimei’, while *RsMYB2* and *RsMYB3* regulate red skin in *R. sativus* var. *hortensis* and *R. sativus* var. *sativus*, respectively; 3 primary haplotypes associated with anthocyanin biosynthesis	Expression pattern	Wang et al. 2024a
	*RsDFR.9c* and *RsUGT78D2.2c* regulate anthocyanins; tissue-specific expression of the four copies of *RsPAP1* is crucial in anthocyanin synthesis and accumulation	GWAS/Expression pattern	Liu et al. 2024
	Structural (*RsCHS*, *RsCHI*, *RsDFR*, *RsUGT75C1*) and TFs (*RsTT8*, *RsWRKY44L*, *RsMYB114*, *RsMYB308L*) genes regulate anthocyanin synthesis in black radish root skin	Expression pattern	Zhang et al. 2023
	*RsMYB1* and *RsTT8* expression, determine anthocyanin content	Expression pattern	Lim et al. 2023
	Five structural (*RsTT4*, *RsDFR*, *RsCCOAMT*, *RsF3H*, *RsBG8L*) and 3 TFs (*RsTT8-1*, *RsTT8-2*, *RsPAR1*) correlated with flavonoids in the skin of the taproot; 4 structural (*RsBG8L*, *RsDFR*, *RsCCOAMT*, *RsLDOX*) and 9 TFs (*RsTT8.1*, *RsTT8.2*, *RsMYB24L*, *RsbHLH57*, *RsPAR2L*, *RsbHLH113L*, *RsOGR3L*, *RsMYB24*, *RsMYB34*L) genes correlated with metabolites in the flesh of the taproot	Expression pattern	Zhang et al. 2021
Sweet potato (*Ipomoea batatas*)	*IbMYB75*, *IbMYB3*, *IbMYB6* and *IbMYB4* cooperatively regulators of root skin color	Expression pattern	Zhang et al. 2025a
	282 and 216 genes were up-regulated, while 2 and 11 genes were down-regulated at the highest and lowest development stages of anthocyanin contents, respectively; DEGs involved in flavonoid biosynthesis pathway include *CCoAOMT*, *PpLDOX*, *DFR*, *CytochromeP450*, *CHI*, and *CHS*	Expression pattern	Xiong et al. 2022
	27 DEGs involved in carotenoid metabolism and 15 TFs significantly associated with carotenoid content;	Expression pattern	Jia et al. 2022
	358 genes implicated in multiple pathways for the biosynthesis of anthocyanins, of which 26 DEGs involved in Aux/IAA-ARF signaling	Expression pattern	He et al. 2020
	*Orange* and *phytoene synthase* genes regulate β-Carotene; an inverse relationship between β-carotene and starch content due to physical linkage of physical linkage of *phytoene synthase* with *sucrose synthase*	QTL analysis	Gemenet et al. 2020
	Two major loci for root carotenoids, mapped on chr 1 at positions 24.1 and 30.5 Mbp; a single locus for dry matter content co-located with the 24.1 Mbp peak for carotenoids; candidate gene *phytoene synthase* (carotenoids), and *UDP-glucose pyrophosphorylase* and *sucrose synthase* (starch) biosynthesis within the vicinity of locus at 24.1 Mbp	GWAS	Rabbi et al. 2017
Turnip (*Brassica rapa* subsp. *rapa*)	Purple turnip strongly down-regulates one flavonol synthase gene, while strikingly up-regulates dihydroflavonol 4-reductase (DFR), anthocyanidin synthase and UDP-glucose: flavonoid-3-O-glucosyltransferase genes as compared to green turnip; MYB, bHLH and WRKY family genes drive transcriptional changes between the two turnips	Expression pattern	Zhuang et al. 2019
Potato (*Solanum tuberosum*)	*StMYB200* and *StMYB210*, within the *Pf* locus are involved in anthocyanin biosynthesis in tuber flesh; 1.7-kb insertion within *StMYB210* promoter causes a change in flesh color	GWAS/genome editing	Du et al. 2025
	*StHY5* and *StF3’H* regulate anthocyanin (cyanidin) accumulation under light*; StHY5* activate *StAN2, StCHI*, *STF3H*, while *StAN2* further activate StF3’H *and StDFR*	Expression pattern/transgene	Zhang et al. 2025c
	*StWRKY70* promotes anthocyanin accumulation in tuber flesh pigmentation by interacting with *StAN1*	Expression pattern	Zhang et al. 2024
	*St5GT* in the anthocyanidin biosynthesis pathway was markedly upregulated in the purple skin and flesh	Expression pattern/transgene	He et al. 2024
	GWAS detected 5 QTL associated with steroidal glycoalkaloid accumulation, of which *Sga1.1, Sgr8.1*, and *Sga11.1* were validated in genetic population; *Sga5.1* and *Sga7.1* mapped in genetic population but not in GWAS; *Sga1.1*, *Sga7.1*, *Sgr7.1*, and *Sgr8.1* co-localized with *GAME9, GAME 6/GAME 11*, *SGT1*, and *SGT2* genes, respectively	GWAS/QTL analysis	Vos et al. 2022
	A total of 497 SNPs were associated with folate content, of which 42 SNPs mapped on chr 6 and 4; additional significant SNPs were located within or in proximity to folate metabolism-related genes	QTL analysis	Bali et al. 2018
Yam (*Dioscorea* spp.)	Expression levels of MYB and WRKY TFs genes were strongly associated with anthocyanin biosynthesis under light conditions	Expression pattern	Zhang et al. 2025b
	108 DEGs associated with the flavonoid synthesis pathway, with 57 up regulated in purple tubers, including *CHS*, *CHI*, *DFR*, *FLS*, *F3H*, *F3′5′H*, *LAR*, *ANS*, *ANR*; 424 metabolites, including 104 flavonoids and 8 tannins, accumulated differentially in purple and white tubers; 57 genes regulated 20 metabolites within the flavonoid synthesis pathway	Expression pattern	Wang et al. 2024c
	A SNP mapped on Chr 10 explained 64% PVE associated with diosgenin content; SNP located in the promoter region of *CYP94D144*, a member of P450 gene family involved in biosynthesis of diosgenin from cholesterol	GWAS	Sun et al. 2024
	*Dj*CYP90, the orthologous gene of CYP90G4 in *P. polyphylla*, CYP90B50 in *T. foenum-graecum*, CYP90G6 in *Dioscorea zingiberensis*, and CYP90G in *Dioscorea villosa*, encodes a diosgenin biosynthetic rate-limiting enzyme, with expression levels significantly upregulated in *D. japonica* bulbils tubers, a species with diosgenin content	Expression pattern	Onoda et al. 2023

Four QTLs regulate sugar composition in onion (Lee et al. 2021). The characteristic onion flavour is due to *A. fistulosum* γ-glutamyl transpeptidase 1 (AfGGT1). It catalyses the production of S-1-propenyl-L-cysteine from γ-glutamyl-S-1-propenylcysteine, a crucial intermediate in the biosynthesis of isoallin (a sulphur-containing compound), to release unique onion flavour (Zhu et al. 2025). QTLson chromosomes (Chr) 3 and 5 are associated with pungency and bulb soluble solids content, with a pleiotropic effect on onion bulb carbohydrate composition. *ATPS* and *SiR* loci are significantly associated with bulb pungency but not with bulb solids content (McCallum et al. 2007).

The annotated 7 genes in cassava, including *itf04g30630* (galactose metabolism); *itf03g13270* and *itf15g09320* (starch and sucrose metabolism); *itf14g10250* (carbohydrate metabolism); *itf12g19270* (carbohydrate and amino acid metabolism); *itf03g21950* and *itf15g04880* (amino sugar and nucleotide sugar metabolism) are involved in sugar and starch metabolism (Nie et al. 2023). Cassava SNPs mapped on chr 7 are significantly associated with various gari traits (Aghogho et al. 2024), while several SNPs and candidate genes, including *Manes.13G026900* and *Manes*.*13G026900*, determine root mealiness in cassava (Uchendu et al. 2021). Sweetness is a main component of the table beet flavour profile and a key determinant of its market value for fresh consumption. A major QTL on chr 2 mapped near the gene determines the degree of sweetness in table beet accessions, while another QTL on chr 7 differentiates table beet accessions for sweetness (Pelikan and Goldman 2024). *UDP-glucose 4-epimerase* genetically balances sugar accumulation and cell wall synthesis in sugar beet (Wang et al. 2024a). A few genes and SNPs are significantly associated with high fibre content in sweet potato (Kim et al. 2025). Multiple QTL with major effects control dry matter, sugar, starch, and protein contents in yams (Arnau et al. 2024). mRNAs, CircRNAs, and miRNAs are non-coding RNAs that play significant roles in gene expression and regulation (Buccitelli and Selbach 2020; Hakim et al. 2025). RNA genes regulate nutritional quality (starch and sucrose) of corm, a root storage organ in taro (Dong et al. 2021).

Multiple SNP loci are associated with several nutritional, pasting, and textural qualities, with some loci having pleiotropic effects in potato. Many of these loci are located near genes involved in starch biosynthesis and metabolism, thereby contributing to their critical roles in starch functionality (Ahmad et al. 2025). Genes associated with altering the starch granule size and shape can be used to create novel starches with altered physicochemical and/or functional properties (Hochmuth et al. 2025). Numerous genes and their allelic variants control steroidal glycoalkaloids (SGAs) in potato (Li et al. 2024a; Vos et al. 2022), while *SUT* family genes and SWEET-mediated sucrose transport (*DrSWEET6* and *DrSWEET12*) regulate sucrose partitioning and accumulation, cell expansion, and biomass accumulation in tuber development (Li et al. 2025b, 2025c). The genetic basis of boiled yam tuber quality remained elusive until recently, when three metabolic pathways and 22 genes linked to texture, starch content, and colour were elucidated (Mota et al. 2024).

Multiple genes and QTL control anthocyanin and carotenoid accumulation, for example, 3 major-effect QTL regulate cyanidin- and peonidin-based anthocyanins in onion (Choi et al. 2020) and carotenoid biosynthesis in carrot (Coe et al. 2023; Deng et al. 2025; Rolling et al. 2025). Carrot accumulates anthocyanins in outer and inner phloem tissues, but with distinct gene expression patterns (Bannoud et al. 2021). Numerous candidate genes (*RST1*, *RsMYB1*, *RsMYB2*, *RsMYB3*, *RsDFR.9c*, *RsUGT78D2.2c RsTT8*), an several structural and TF genes, regulate anthocyanin biosynthesis and accumulation in radish (Lim et al. 2023; Liu et al. 2025, 2024a; Wang et al. 2024b; Zhang et al. 2023, 2021).

Widespread pleiotropic loci, rather than genetic linkage, drive the inverse relationship between total carotenoid and dry matter content in cassava (Villwock et al. 2025), while a mutation known to influence β-carotene content in PSY transcripts significantly enhances β-carotene content (Olayide et al. 2023). Other reports indicate significant marker-metabolite relationships and candidate genes associated with β-carotene biosynthesis and accumulation in cassava (Ding et al. 2023; Fu et al. 2022). *phytoene synthase* is a putative gene for carotenoids, and the *UDP-glucose pyrophosphorylase* and *sucrose synthase* regulate starch biosynthesis in cassava (Rabbi et al. 2017). In the sweet potato genome, several genes regulate carotenoid and flavonoid biosynthesis and accumulation (Gemenet et al. 2020; Rabbi et al. 2017; Xiong et al. 2022; Zhang et al. 2025a). A study involving genomic and metabolomic resources of purple and green turnip accessions detected differential anthocyanin accumulation and candidate structural genes and key TFs mediating anthocyanin biosynthesis in turnip (Zhuang et al. 2019).

Recent research unlocked multiple genes associated with anthocyanin biosynthesis and accumulation in potato, for example, *StMYB200* and *StMYB210* within the *Pf* locus in tuber flesh (Du et al. 2025), *StWRKY70* interacts with *StAN1* in tuber flesh (Zhang et al. 2024), *St5GT* regulate anthocyanin both in skin and flesh (He et al. 2024), and *StF3’H* regulate cyanidin-type anthocyanin under light conditions (Zhang et al. 2025c). Forty-two SNPs mapped on chr 6 and 4 were significantly associated with folate or vitamin B_9_ (Bali et al. 2018). *Dj*CYP90 is a diosgenin biosynthetic rate-limiting enzyme, which is significantly upregulated in *D. japonica* bulbils tubers (Onoda et al. 2023). A major SNP mapped on chr 10, located in the promoter region of *CYP94D144,* is involved in the biosynthesis of diosgenin *Dioscorea zingiberensis*, a yam species with substantial variation in diosgenin content among its accessions (Sun et al. 2024).

In summary, numerous putative genes and genomic regions (QTLs) have been identified across these crops, which should be functionally validated across genetic backgrounds and environments in target breeding germplasm or populations before use in crop breeding. Several approaches exist to characterize gene functions, including transgene, genome editing, RNAi gene silencing, gene knockout, and virus-induced gene silencing. A central finding of this review is that despite the identification of a vast number of QTL, single nucleotide polymorphisms (SNPs), and candidate genes for key traits in RTB crops, a significant gap remains in translating these discoveries into practical breeding tools, as very few have been proven causal, stable, or useful across diverse genetic backgrounds and environments. Bridging this gap requires addressing fundamental scientific uncertainties, such as determining the true reproducibility of QTL across breeding populations, confirming genuine causality, and evaluating effect stability across varying environmental conditions and ploidy levels. Ultimately, future research must establish whether DNA markers identified in diversity panels can reliably predict performance in applied RTB breeding programs.

## Metabolite profiling to identify genes associated with non-nutrient (secondary metabolites) traits

Metabolic profiling of nutritionally significant traits in RTB crops has been conducted in potato, sweet potato, onion, yam, carrot, and cassava, but taro and others have received much less attention. Zhang et al. (2018) found 27 unigenes involved in anthocyanin biosynthesis, along with 16 differently expressed genes (DEGs) playing a role in flavonoid biosynthesis in onion. Dark red bulbs were formed largely through the action of flavonoid 3′,5′-hydroxylase (F3′5′H) and dihydroflavonol 4-reductase (DFR) genes. Castro-Cegri et al. (2025) grew onions in a hydroponic system with varying levels of Hoagland’s solutions and used ultra-high-pressure liquid chromatography along with quadrupole-time-of-flight mass spectrometry to assess metabolic profiles under nutrient stress. They found certain dilutions of Hoagland’s solution were associated with upregulation of antioxidant capacity and enzyme inhibitory activity, primarily due to increases in phenolic and flavonoid compounds. These, in turn, may be associated with improvements in medicinal traits such as neuroprotective and antidiabetic properties.

Yang et al. (2025a) conducted analyses of potato tuber tissues to investigate the metabolome associated with anthocyanin production. They found 1,924 genes with potential involvement in anthocyanin biosynthesis, and 47 of these genes were specific to tuber expression and correlated with anthocyanins. This smaller subset of genes was associated primarily with ATP-binding cassette transporters and hormone pathways. The transcription factor *StWRKY44* appeared to play a prominent role in anthocyanin biosynthesis by activating promoters of 7 structural genes for anthocyanins. Production of reactive oxygen species (ROS) is regulated by antioxidant enzyme defences such as glutathione reductase, catalase, and peroxidase (Siviya et al. 2025). These antioxidant defences are also upregulated in sweet potato and potato in response to some external stresses (Meng et al. 2020), but downregulated under cold stress (Cui et al. 2020). Wang et al. (2025) conducted an RNA-sequencing analysis, which revealed 19,377 and 13,741 differentially expressed genes in browning-resistant and browning-susceptible potato tubers, respectively. Up-regulated genes were enriched for phenol and lipid biosynthesis. Browning-susceptible tubers were better able to produce phenolic compounds than browning-resistant tubers, but the latter had superior free radical scavenging ability. The flavonoid biosynthetic gene *StDFR (*dihydroflavonol reductase) had higher expression in red and purple fleshed potato tubers, while *StYUCCA* (indole − 3-pyruvate monooxygenase) had higher expression in yellow tubers (Wu et al. 2024a). Li et al. (2024b) found a coordinated upregulation of genes within the anthocyanin biosynthesis pathway in red and purple-fleshed potatoes. MYB transcription factors were implicated as regulators of this process. Similar transcript levels were found for carotogenic genes such as lycopene cyclase and carotene desaturase in both potato and the wild, yellow-fleshed *Solanum phureja* (Morris et al. 2004).

Sun et al. (2025b) studied 30 genes associated with anthocyanin biosynthesis in yams and determined 4CL (4-coumarate CoA ligase) and DFR (dihydroflavonol 4-reductase) were likely regulators of anthocyanin pigmentation variation between skin and flesh. Cao et al. (2023) found 743 differentially expressed genes for starch and sucrose metabolism in yams, hormone signal transduction pathways, and the phenolpropanoid pathway through comparative transcriptomics. Using RNA-seq, Wu et al. (2024b) found similar engagement of the phenylpropanoid pathway genes during a series of developmental stages in sweet potato. They found that the S3-S4 stage was the key stage of root development of sweet potato with respect to the accumulation of these metabolites. Co-expression network analysis revealed that differential transcriptomic responses were associated with differences in fructose and mannose metabolism, pentose phosphate, selenium compound metabolism, glycolysis/gluconeogenesis, and carbon metabolism. Wan et al. (2024) found differentially accumulated metabolites among differently coloured sweet potatoes. They identified 2,808 metabolites with differential accumulation among white (BS), orange (CS) and purple (ZS) cultivars. Interestingly, sweet potatoes were found to contain L-theanine, typically found in tea and associated with relaxation. Many potentially valuable compounds, such as lentinic acid and gambogic acid, were identified during screens to profile metabolites. Wan et al. (2025) profiled more than 3,000 metabolites across a period of postharvest storage in sweet potato. Their results demonstrate fluxes in metabolic pathways, including phenylpropanoid, flavonoid, and glycolytic pathways, including the upregulation of certain volatiles during extended storage.

Olayide et al. (2023) performed comparative transcriptomic and metabolic analyses of roots and leaves of eleven African cassava landraces, focusing on genes associated with carotenoid biosynthesis. They identified a mutation in *PSY* transcripts of deep yellow, but not of pale yellow, genotypes. NCED_3.1 was expressed at a lower level in all yellow genotypes, suggesting that it could be a potential target for increasing carotenoid accumulation in cassava. They also identified metabolites that were correlated with Beta-carotene content. He et al. (2025) studied metabolites in purple, yellow, red, and orange carrots using untargeted metabolomics in conjunction with bioinformatics. Purple carrots were rich in flavonoids, while yellow carrots were enriched for anthracenes and curcumin. Orange carrots were mainly associated with gamma-crocetin. Several metabolites associated with *in* vivo antioxidant activity were identified, and their influence was determined to be through the nitrogen metabolic pathway. The Orange (OR) gene has also been linked to carotenoid accumulation in several crops, including sweet potato and carrot (Ellison et al. 2018; Kim et al. 2013). This gene interacts with phytoene synthase to influence chromoplast formation and, ultimately, carotenoid content. It is important to note that the target organs in these examples: cassava, sweet potato, and carrot, possess different anatomical features and root structures, which could be responsible for differences in metaboloic pathways in these different crops.

Cassava plants exhibit both storage and fibrous roots. The differences in these roots were recently clarified by Carluccio et al. (2022), who found that storage roots are a functionally different root type from fibrous roots. The fate of a storage root occurs early in development. Storage root swelling is the consequence of starch synthesis in specialized parenchymatous root tissue, specific to a subset of emerging roots that follow a different rhizogenic process than fibrous roots. Transcriptome analysis showed activation of several functions essential for root swelling and identified root‐specific genes able to effectively discriminate between storage and fibrous roots. These genes may be important to modifying and improving starch biosynthesis in cassava.

## Uptake, transport and storage of nutrient and non-nutrient traits in edible vegetable organs and their effects on consumers

Many RTB crops function as fleshy storage organs that accumulate both essential nutrients and non-nutrients absorbed from soil and transported throughout the plant via the xylem and phloem for various physiological functions. These nutrients influence human nutrition when consumed, either by promoting health or posing dietary risks depending on concentration, illustrating both beneficial and harmful accumulations.

Nitrates, for example, have both nutritive and anti-nutritive properties in table beet, radish, and turnip. Nitrogen, preferentially taken up as nitrates via nitrate transporters, is transported through the xylem to the leaves and assimilated into organic forms that are used as building blocks (proteins, DNA, etc.) (Kant 2018). When nitrate uptake exceeds assimilation rate, nitrate can be stored for later use, particularly in the reproductive stage of biennial crops. Growing conditions strongly influence nitrate accumulation. Cantliffe (1973) found that increased nitrogen fertilizer increased nitrate accumulation in table beet tissue, and lower light intensity (which could correspond to growing season) further enhanced nitrate accumulation by limiting assimilation. Fertilizer source also matters, with organic fertilizer reducing nitrate levels in radish, which is often used in developing countries from manure or compost (Wang et al. 2016). Genetic variation is also present, as Grzebulus and Baranski (2001) found significant differences in nitrate accumulation amongst 40 table beet cultivars, alluding to breeding potential. From a dietary perspective, radish and turnip are classified as high accumulators, while table beet is considered a medium to high accumulator (Colla et al. 2018). Although high nitrate intake has historically been associated with health risks such as methemoglobinemia in infants from baby food products, more recent research has demonstrated potential health benefits, including improved cardiovascular performance through the nitrate-nitrite-nitric oxide pathway in adults (Karwowska and Kononiuk 2020; Sanchez-Echaniz et al. 2001).

Heavy metal contamination (As, Cd, Co, Cr, Cu, Ni, Pb) can occur in most RTB crops, with soil contamination levels as the primary driving factor. Regions in the Global South—including parts of the Middle East, South Asia, and southern China—have been deemed as “metal-enriched corridors” (Hou et al. 2025), where conditions that accelerate weathering, combined with mining and industrial activity, increase the bioavailability of heavy metals in soils (Hou et al. 2025; Sharma and Nagpal 2019). Cadmium, for example, can enter plants through other divalent cation transporters when high concentrations are present in soil. Cd can accumulate in crops like sweet potato and carrot in the edible root organs (Tońska et al. 2020; Huang et al. 2015). Similarly, lead in carrots (Tońska et al. 2020), arsenic in potatoes (Kundu et al. 2012), and copper in taro (Cobbinah et al. 2025) can also accumulate, among other examples. These heavy metals can cause kidney failure, cancer, neurological disorders, infertility, and developmental delays in children. Although individual samples may not exceed regulatory limits, consumption can be especially harmful when eaten as staple crops, which is common in the Global South. At a minimum, it is important to wash and peel crops before consumption in areas of contaminated soil.

Some beneficial nutrients taken up also have unintended effects. Calcium is taken up from the soil solution and transported upward through the xylem, mainly driven by transpiration rate and the flow of water. Ca is an important part of structural integrity, signalling, and storage quality. Alternatively, calcium can also form antinutritional compounds with oxalic acid in the plant called oxalates. While primarily a mouth and throat irritant, oxalates can reduce the bioavailability of calcium to the body and, in severe cases, contribute to kidney stone formation. These compounds are common and abundant throughout taro and table beet plants. Freidig and Goldman (2011) reported values ranging from 95 to 141 mg oxalate/100 g table beet tissue, and Catherwood et al. (2007) reported values ranging from 73.2 to 171.4 mg oxalate/100 g taro root tissue. Blanching or boiling has been shown to significantly reduce oxalates through leaching, whereas baking has been shown to concentrate them.

Sulphur is actively taken up via sulphate transporters, transported through the xylem, and redistributed in the phloem as sulphur-containing compounds. Selenium, as selenate, can pass through these same transporters due to its similarity to the sulphate ion, particularly accumulating in onions and garlic, where it is incorporated into sulphurous compounds as a substitute (Gupta and Gupta 2017). Selenium is not essential for plants, but is for humans, with plants serving as the main dietary source. Adults require 45–55 µg Se/day (Institute of Medicine 2000). Reported concentrations in onion and garlic are 5.4 and 10.5 µg Se/kg fresh weight, respectively, under low-selenium soil conditions, but can increase substantially with greater soil selenium availability (De Temmerman et al. 2014). RTB crops are also a healthy source of other nutrients such as potassium, magnesium, and zinc.

## Advances in genetic improvement of nutritional traits in root and tuber crops

*Crossbreeding and selection:* Recent advances in the genetic improvement of bulb, root, and tuber crops have shifted from traditional selection methods toward integrated breeding systems—approaches that combine conventional crossbreeding with molecular tools to enhance efficiency and precision. Conventional hybridization remains the foundation for generating genetic diversity in crops such as cassava, potato, sweet potato, taro, and yams, which are valued for their high carbohydrate content and resilience to marginal environments (Abincha et al. 2024; Divya et al. 2024; Rodríguez et al. 2025). However, traditional recurrent selection schemes are often hindered by long breeding cycles and biological constraints, such as erratic flowering and complex polyploidy (Mbanjo et al. 2024; Xiao et al. 2022). Addressing these challenges is particularly important for improving nutritional traits—such as increased provitamin A carotenoids, zinc, and iron—that are critical for food security and public health. To overcome these hurdles, breeders are increasingly deploying marker-aided selection (MAS)—the use of DNA markers to select plants with desirable traits—and genomic prediction models, which use genome-wide data to predict breeding values, to accelerate genetic gains for nutritional traits such as increased provitamin A carotenoids, zinc, and iron in cassava (Abincha et al. 2024; Behera and Singh 2019).

A novel SNP *SssI*-*C *_*45711901*_* T* and *InvGE*-*C *_*2475454*_* T* associated with potato frying colour and tuber sugar content in diploid *Phureja* germplasm, respectively (Duarte-Delgado et al. 2017), while a large-scale discovery of a biased accumulation of deleterious single-nucleotide polymorphisms (dSNPs) around deleterious structural variants (dSVs) in the coupling phase may allow molecular breeders to computationally design an ideal potato haplotype with minimal dSNPs and dSVs for use in potato breeding (Cheng et al. 2025a, b). High genomic prediction accuracy for most traits may assist turnip rape breeders to accelerate genetic gains (Alamu et al., 2024). Perfect co-segregation of KASP markers may allow cassava breeders to combine cassava mosaic disease resistance, dry matter content and total carotenoids content into progenies (Esuma et al. 2022). The widespread pleiotropy, rather than the genetic linkage, drives the inverse relationship between total carotenoids and dry matter in cassava (Villwock et al. 2025), while two QTL act pleiotropically to reduce starch content and increase β-carotene in germplasm (or segregants) carrying the orange-fleshed parental haplotype at the LG3 locus (Gemenet et al. 2020). Hence, unlocking the molecular basis of the inverse association between competing traits may inform breeding strategies to enhance food and nutritional security in sweet potato.

Modern biofortification initiatives, such as the CGIAR HarvestPlus (Bouis et al. 2024), have successfully utilized both crossbreeding and advanced genomic tools to enhance the nutrient density of staple roots (Medina-Lozano and Díaz 2022). In cassava, the application of Kompetitive Allele-Specific PCR (KASP)—a genotyping technology for detecting specific genetic variants—and DArTseq markers—a high-throughput sequencing-based genotyping method—allows for precise quality control and the identification of genomic regions linked to high dry matter and carotenoid content (Mbanjo et al. 2024). Furthermore, high-throughput phenotyping (HTP) technologies, particularly non-destructive methods such as near-infrared spectroscopy (NIRS), enable breeders to rapidly and efficiently screen large breeding populations for important chemical constituents like starch, protein, and minerals, overcoming previous limitations related to cost and time (Alamu et al. 2020; Lebot et al. 2009). These integrated approaches not only improve the speed of selection but also contribute to the development of cultivars with improved taste and processing quality, which may help support food security in tropical and sub-tropical regions (Naconsie and Zhang 2016; Xiao et al. 2022).

*Exploiting Intraspecific and interspecific variation:* Ebert (2020) noted that global vegetable crops are well represented in global gene banks, but much less so for traditional vegetables. This is particularly true in Africa and Asia, where some traditional vegetable genetic resources may be replaced by modern cultivars. Ebert suggests collections from home gardens and community seedbanks for those interested in sourcing germplasm that may be useful for crop breeding efforts.

Among the best examples in the improvement of nutritional traits in root and tuber crops is the development of orange-fleshed sweet potato (OFSP) cultivars during the twenty-first century. Vitamin A deficiency is endemic in sub-Saharan Africa and parts of Asia, causing night blindness in children. This situation is particularly acute in countries like Mozambique (Andrade et al. 2017). Vitamin fortification of processed foods may also be useful in this situation, but it may not reach those in poor, rural areas. Andrade et al. developed a polycross nursery, utilizing intraspecific variation, at two locations for the development of new clones adapted to Mozambique. They used the ABS technique, which asserts that temporal variation in testing environments is equivalent to spatial variation in testing environments in the earliest stages of a breeding program. They selected for carotene content over a period of years to develop OFSP cultivars that are now used globally. The work associated with the development of OFSP was selected for the World Food Prize in 2016.

Carrot is among the most important contributors to Vitamin A nutrition of the root crops, as β-carotene concentrations are known to be very high in modern orange carrots. Substantial efforts to improve β-carotene content in carrots began in the 1950s. Banga et al. (1955) pointed out that effective selection for carotene content in carrots should consider both size and maturity. Simon et al. (1989) developed the high carotene mass carrot population from a cross of [(‘Kokubu’ x B2158)] x [(B3079 x ‘Imperial Long Scarlet’] that was made in 1974, utilizing intraspecific variation. This population exhibited 460–499 ppm carotene, with β-carotene accounting for 45–65% of the total carotene content. Continued selection over many decades has pushed β-carotene levels up over 10 mg / 100 g of fresh weight in modern cultivars, a considerable increase over older, heirloom types. Coe et al. (2023) highlighted three recessive genes: *REC*, *Or*, and *Y2*, which were associated with high α- and β-carotene orange carrots. They found that these three genes influenced carotenoid accumulation by regulating carotenoid and chromoplast biosynthesis. Rolling et al. (2025) found a QTL associated with phytoene synthase, which is a crucial step in the biosynthesis of carotenoids. They acknowledged that while *Ys, Y, Or,* and *REC* are mostly fixed in orange-rooted carrots within *Daucus carota*, substantial variation for carotenoid content suggests interaction of these genes with each other and environmental factors. The CRISPR/Cas9 system was successfully used to target a crucial step in carotenoid biosynthesis, the *Phytoene Desaturase* (PDS) gene in yams, and superior promoters were identified (Syombua et al. 2021). This type of intraspecific variation is likely to play a greater role as gene editing techniques become more prevalent.

Bredeson et al. (2016) developed a high-quality genome assembly for cassava and sequenced a collection of 58 *Manihot* accessions, including cultivated and wild accessions, along with wild relatives of the crop as well as Indian rubber. They found evidence of significant interspecific admixture in the cassava genome. Attempts to enhance amino acid, triacylglycerol, and Vitamin E levels through the overexpression of several genes, including *Wrinkled1, PrLeg, Ama1,* have been attempted in potato*.* Changing the ratio of amylose to amylopectin can influence the properties of starch. Wang et al. (2019) attempted to modify starch quality in sweet potato using a gene editing approach with the starch biosynthetic genes IbGBSSI and IbSBEII. Total starch content relative to the wild-type was not significantly altered in IbGBSSI- and IbSBEII-knockout transgenic lines. However, it reduced amylose in IbGBSSI-knockout lines, while in IbSBEII-knockout lines, it increased amylose. RNAi has been used to target the granule-bound starch synthase 1 gene in cassava order to reduce amylose levels, which results in starch with improved industrial and functional properties. Bull et al. (2018) edited starch synthase genes in cassava and achieved modifications in starch quality that were also beneficial for industrial processing. Interestingly, they also made use of a FLOWERING LOCUS T gene to speed up flowering and advance breeding cycles more quickly. Andersson et al. (2018) developed low amylose potato genotypes by targeting similar starch biosynthetic genes; in this case GBSS1.

Agaba et al. (2016) found substantial genetic variation for yield and quality traits in yam bean, indicating that breeding may be a feasible approach to improving nutritional quality. Complementary to this genetic approach, Modi (2025) grew and compared small, medium and large taro propagules from a local landrace. Propagule size was associated with both productivity and nutritional value, with a positive correlation between propagule size and starch, protein, and macronutrient content. However, micronutrient content was decreased in larger corms. These findings highlight that nutritional quality is not only determined by genetic improvements, but also its interaction with the growing environment.

### Reducing bitterness in carrots and cyanide in cassava, and enhancing the biomolecule in garlic (alliin) and yams (diosgenin)

*Bitterness:* Bitterness causes an undesirable taste in carrots, but protects the crop from soil-borne fungal pathogens and may protect humans from chronic noncommunicable diseases (Ahmad et al. 2019; Alfurayhi et al. 2023; He and Dunemann 2025). The breeding of resilient and nutritious RTB crops often faces biological trade-offs where prioritizing one desirable trait inadvertently compromises another, such as the inverse relationship between high biomass yield and dense micronutrient accumulation or the metabolic competition between starch synthesis and carotenoid deposition. Because conventional single-trait selection often exacerbates these physiological antagonisms—yielding crops that thrive in the field yet fall short in nutrition, palatability, or defence chemistry—modern breeding must pivot toward integrated, systems-level frameworks. By harnessing advanced approaches like multi-trait genomic selection (Moeinizade et al. 2020), economic and nutritional selection indices (Cerón Rojas and Crossa 2018), and Pareto optimization models (Akdemir et al. 2019), breeders can map and untangle complex pleiotropic effects. Rather than relying on arbitrary targets, this multifaceted approach maps the optimal trade-offs between competing goals, enabling researchers to generate a spectrum of elite germplasm and develop cultivars that maximize nutritional value without falling below acceptable yield thresholds, ultimately transforming a rigid biological compromise into a strategic advantage.

Falcarinol (FaOH) and falcarindiol (FaDOH) are the main falcarinol-type polyacetylenes (PAs) associated with bitterness in carrots. Limited investigations have revealed sufficient genetic variation in bitterness in carrot germplasm (Christensen and Kreutzmann 2007; Kreutzmann et al. 2008). Major QTL and putative genes (*FAD2* and *CER1*) mapped on chromosomes 4 and 9 are associated with FaOH and FaDOH contents (Dunemann et al. 2022). Moderate to high heritability and low genotype-environment interaction for bitterness and soil-borne pathogen (*Alternaria dauci*) in breeding populations suggest that it is feasible to increase resistance by selecting for low-bitter cultivars, targeting genomic regions with inverse relationships between competing traits (Le Clerc et al. 2019).

*Alliin:* it is the most abundant non-protein S-containing compound in garlic leaves and cloves. Crushed or chopped garlic leaves release the enzyme alliinase, which converts alliin into allicin and other thiosulfinates, and is associated with a characteristic aroma. Alliin provides numerous health benefits, including antioxidant and anti-inflammatory properties (Borlinghaus et al. 2014; Seki and Hosono et al., 2025). Garlic germplasm shows sufficient variability for alliin and allicin concentrations, and a strong relationship between yield, leaf mineral content, and, potentially, downstream allicin concentrations, which can assist in the selection of specific high-alliin garlic lines (Nguyen et al., 2024). A functionally characterized gene, *AsWRKY9,* may be deployed to breed garlic germplasm with high alliin content (Wu et al. 2025a).

*Cyanide:* Hydrogen cyanide (HCN) in fresh cassava roots is an antinutrient compound due to the presence of cyanogenic glycosides, which limit the use of cassava as food or feed, as it may cause poisoning and other health issues. Hence, adequate processing (which adds cost) or low-HCN cultivar (most economic) is necessary to reduce HCN levels in cassava (Moses et al. 2025). Adoption of near-infrared spectrometry (NIRS) coupled with machine learning algorithms is a fast and reliable approach, with minimal sample preparation time, to discriminate low from high HCN accessions (Kanaabi et al. 2024a). Cassava clones show sufficient variability for HCN content. The presence of significant clone-environment interaction, HCN significantly correlated with mean daily high temperature and lower humidity values, and low heritability across environments may pose a significant breeding challenge for developing low HCN cultivars (Kanaabi et al. 2024b). Significant progress has been made toward unlocking genes and SNP (S16_640082 and S16_735381 SNP) and KASP markers (snpME00404, snpME00405, snpME00406) which could be put in use, after functional validation in the targeted breeding populations across genetic backgrounds and environments, to develop stress-tolerant, productive, and nutritionally enhanced cassavas with low HCN content for boiled consumption (Chaicharoen et al. 2023; Kanaabi et al. 2024c)**.**

*Diosgenin:* Diosgenin is an important source material for the preparation of steroidal drugs in the pharmaceutical industry (Semwal et al. 2022). *Dioscorea zingiberensis* is an excellent source of diosgenin. It accumulates 2–16% diosgenin in its rhizomes. GWAS detected a significant SNP-trait association on chr 10 (64% PVE), located in the promoter region of *CYP94D144* involved in the biosynthesis of diosgenin, and validated involving high- and low-diosgenin-producing hybrids from a bioparental population (Sun et al. 2024). *DZHDZ32* is a potential regulator of diosgenin biosynthesis in *D. zingiberensis*. Transgenic *D. zingiberensis* plants overexpressing *DZHDZ32* significantly increased diosgenin levels by 41.68% and 68.07% in leaves compared to WT (Yang et al. 2025b), which might eventually be deployed in marker-assisted selection to improve diosgenin levels in yam cultivars. Diosgenin is synthesized in leaves, converted into diosgenin saponins, and transported to rhizomes for storage (Li et al. 2022).

*Pathways shaping storage development and nutrient accumulation:* The genetic improvement of RTB crops has increasingly focused on the complex regulatory networks that govern sink strength—the ability of tubers and storage roots to import and store photosynthates. Advances in functional genomics have identified that the transition from root to storage organ is a developmental switch controlled by systemic signalling molecules, hormonal crosstalk, and specific sugar transporters. The discovery of the *StSP6A* gene, a member of the *FLOWERING LOCUS T* (*FT*) family, in potato has been pivotal (Plantenga et al. 2019); it acts as a tuberigen signal that moves from leaf to stolon to initiate tuberization in response to daylength. While heat stress directly suppresses the StSP6A signalling pathway in potato, the overexpression of *StHsfA2* brings thermotolerance through *StSP6A* (Zhang et al. 2025d)*,* thus promoting tuberization in this crop when grown at high temperature.

Shi et al. (2024) provided important insights into the molecular biology coordinating tuberization, leaf senescence, and sugar transport, which underlie source–sink relations and determine tuber yield in potato. This research has been recognized as a landmark study due to the multifaceted regulatory mechanisms and pathways involved (Tai 2024). CRISPR/Cas9 gene editing may allow modulating the expression of *StSP6A* and its inhibitors, such as *StCEN* (Zhang et al. 2020a, b), to reduce the dependence of tuberization on strict environmental cues, which may contribute to more stable nutritional accumulation under heat stress.

Beyond initiation, the rate of tuber and storage root expansion directly influences the concentration of starches, proteins, and micronutrients. This process is largely governed by the metabolism of sucrose and its unloading into storage cells. Key targets for genetic enhancement include *Sucrose Synthase* (*SuSy*) and *Vacuolar Invertases* (*VInv*). In root crops such as sweet potato and cassava, regulating the *IbAGPps* (*ADP-glucose pyrophosphorylase*) gene has achieved significant success in increasing starch yield and modifying the source–sink relationship to prioritize nutrient sequestration (Xiao et al. 2022; Ma et al. 2025). Additionally, the balance between phytohormones—particularly auxins, which promote cell division, and abscisic acid, which regulates maturation—can be fine-tuned through genomic selection to ensure that rapid expansion does not dilute the concentration of essential minerals such as iron and zinc.

Recent breakthroughs also highlight the importance of the vascular system in managing nutrient flux. The development of the cambium, which drives secondary thickening in yams and taro, is regulated by the *WOX* and *CLE* gene families. Advancements in spatial transcriptomics are now enabling plant breeders to map the precise cell layers where expansion occurs, thus facilitating the precision breeding of RTB crops that reach maturity faster while maintaining high nutritional density (Divya et al. 2024). These genetic interventions may facilitate the development of biofactories that provide high-quality calories and micronutrients in a shorter cultivation window.

*Prediction accuracy of genomic selection for carotenoids:* Genomic selection (GS), which uses genomic estimated breeding values (GEBVs), is revolutionizing the breeding of biofortified RTB crops by shifting the emphasis from time-consuming field evaluations to predictive molecular approaches. Traditionally, breeding for nutritional traits such as provitamin A carotenoids or high mineral content required waiting for plants to reach full maturity—a process that can take years, especially in perennial or clonally propagated crops. With GS, plant breeders may use genome-wide marker data to estimate the breeding value of seedlings, enabling rapid selection and recycling of parents without waiting for mature storage roots or tubers.

The main advantage of GS is that it greatly shortens the breeding cycle. For example, in cassava, traditional selection methods can require up to six years to complete one cycle of evaluation and recombination. By implementing GS, breeders can predict the performance of clones at the nursery stage, potentially shortening the cycle to 12 to 24 months (Abincha et al. 2024; Okoma et al. 2026). This acceleration, known as rapid cycling, enables breeders to achieve genetic improvements more quickly, which is essential for addressing urgent nutritional security challenges.

The application of GS differs among species due to variations in ploidy and genetic complexity. Cassava and sweet potato, two root crops at the forefront of biofortification, illustrate these differences. In diploid, outcrossing cassava, GS may enable simultaneous selection for increased total carotenoids and dry matter content. In contrast, sweet potato is a hexaploid (having six sets of chromosomes), highly heterozygous, and self-incompatible (unable to fertilize itself), making crossbreeding challenging. In this polyploid crop, GS helps manage the complex inheritance of traits such as β-carotene content, accelerating the development of orange-fleshed cultivars (Mwanga et al. 2021; Xiao et al. 2022). In potatoes, both diploid and tetraploid types can benefit from GS to improve yield and protein quality, with prediction models accurate enough to replace early-generation field trials. For tetraploid yams, which suffer from an exceptionally long growth cycle and erratic flowering, GS may allow breeders to predict tuber quality early, thereby helping to reduce the multi-year wait for mature tubers (Yu and Cheng 2024). In biennial crops like carrots, GS is particularly useful for selecting nutritional traits such as anthocyanin and carotenoid content during the first year of vegetative growth, thus eliminating the need for vernalization and a second year before selection (Hernandez and Vogel 2026). Although GS is still in its early stages for turnip, efforts are underway to establish training populations. In taro, the availability of a newly assembled reference genome (Sun et al. 2026) provides a foundation to accelerate its genetic enhancement, particularly for key quality characteristics such as starch digestibility and mineral density (Nigussie 2025).

Despite its success, GS faces challenges such as genotype × environment (G×E) interactions and the tendency for high-carotenoid clones in root crops like cassava to have lower dry matter content, which can reduce their appeal to farmers. To address these issues, advanced models now incorporate data from multiple environments and utilize high-throughput phenotyping, refining GEBVs to ensure that accelerated breeding does not compromise stability or important farmer-preferred traits (Moreira et al. 2020; Okoma et al. 2026).

Genomic selection depends on the correlation between GEBVs and observed phenotypes, a measure known as prediction accuracy (ρ_GP_). For nutritional traits such as carotenoid content, prediction accuracy is strongly influenced by the genetic architecture of the trait, the diversity within the training population, and the extent of environmental variation (σ^2^_E_). In many RTB crops, carotenoid accumulation is controlled by a few major-effect genes—such as *Phytoene Synthase 2* (*PSY2*) in cassava and the *Orange* (*Or*) gene in sweet potato—which often results in higher prediction accuracy compared to more complex traits like yield. Reported ρ_GP_ values are high (> 0.5) for total carotenoid content and β-carotene in cassava (Esuma et al. 2021; Ikeogu et al. 2019), but lower to moderate for total carotenoids and α-carotene in carrots (Rolling et al. 2025). These differences in accuracy often reflect whether predictions are made within the same population using cross-validation (which tends to yield higher accuracy) or across different populations (which typically results in lower accuracy). For instance, in cassava, focusing on markers from chromosome 1, where the *PSY2* gene is located, can improve prediction accuracy (Esuma et al. 2021).

The state of the art reveals a stark disparity in the application of advanced genomic-led breeding methods across RTB crops, where major staples like cassava and potato routinely leverage genomic selection to accelerate breeding cycles and enhance genetic gain, whereas taro, yam, and garlic remain in early or primarily prospective stages of genomic integration. This uneven landscape is largely driven by historical research investment, complex polyploid genomes, and high heterozygosity that hinder high-throughput genotyping and phenotyping. Consequently, while frontrunner crops are successfully transitioning from marker-aided selection to predictive genomic models, under-researched species continue to rely heavily on phenotypic selection, underscoring an urgent need to build foundational genomic resources and reference panels tailored to the neglected RTB crops.

*Sequencing genebank diversity involving cultigens, wild, and feral variants:* Friedmann et al. (2018) point out some of the challenges of maintaining RTB crops in gene banks. Crops like potato, sweet potato, and yam are polyploid and often highly heterozygous. Some of the RTB crops, like potato, sweet potato, and cassava, exhibit inbreeding depression, making propagation complicated. Potatoes also exhibit some self-incompatibility, which further complicates multiplication. Yams are a multi-species crop with a complex breeding background, but the citation of scientific references as if yam were a single uniform breeding system, rather than different *Dioscorea* species, complicates downstream interpretations. Vegetative propagation of many of these crops has certain advantages but also possesses disadvantages, as ensuring adequate material for breeding and field trials is more complicated. Generating propagules for distribution can be much more difficult than for diploid crops that are typically propagated by seed. One possible conclusion from these challenges is that more advanced technology must be developed to fully take advantage of DNA sequence information from some RTB crops. Another takeaway from this situation is that specific maintenance, curation, and selection strategies may need to be developed for clonally-propagated polyploid RTB crops compared to diploid, seed-propagated crops.

Hardigan et al. (2017) reported on the sequencing of a collection of wild species, landraces, and cultivars in the USDA potato gene bank located in Sturgeon Bay, Wisconsin. This study showed the depth of importance of wild species introgressions to cultivated potato. Sequence diversity of diploid and tetraploid Solanum tuberosum accessions was far greater than any crop resequencing study up to that point, likely because of key introgressions from wild species following polyploidization. The investigators identified 2,622 genes that were part of important selection responses, including signatures of selection for carbohydrate metabolism, glycoalkaloid production, and many other traits, including genes associated with pollen development and gametogenesis. They were able to determine that certain wild species contributed truncated alleles that contributed to crop maturity in the cultivated germplasm. Ultimately, the modern long-day-adapted potato crop benefited tremendously from wild species introgressions, and it was precisely the sequencing of gene bank accessions that led to these insights.

Carvajal-Yepes et al. (2024) reported on the sequencing of the International Center for Tropical Agriculture’s (CIAT) cassava collection, which holds 5,963 accessions, and their attempt to identify and eliminate redundancy in the collection. Redundancy of accessions is a common problem in crop gene banks. These investigators used empirical distance thresholds and two different molecular marker types on over 5,000 accessions to identify about 2,500 distinct genotypes, some of which were represented by more than 80 redundant accessions.

Removing duplicate and redundant accessions will be an important goal for all RTB crop collections in global gene banks. Balancing this challenge is the fact that many gene bank accessions contain heterogeneity that can be manifest as multiple alleles at certain loci. Accessing any single accession may not represent the full diversity present in the accession for situations such as the presence of rare alleles. Some practitioners have advocated for pooled genotyping (Volk et al. 2021) of gene bank accessions, which can function as a kind of pan-genome. However, a limitation of this approach is that specific alleles may be harder to identify. Regardless, it has become clear that RTB crop germplasm accessions in genebanks are in need of both molecular and phenotypic characterization to make this material useful for the global breeding and research communities. The fact that much of the RTB germplasm in global genebanks remains less accessible because of a lack of characterization provides a mandate for future efforts to improve these crops.

Vega Alfaro et al. (2025) described how information most valuable to the plant breeder or germplasm user community may be the genetic variation in relation to the relatives of the crop, based on evolutionary relationships among accessions. Aubry (2023) and Mascher et al. (2019) suggested that gene banks may shift their primary activities from maintaining and distributing germplasm to providing digital genetic information on accessions, especially as more genetic sequencing takes place. Despite this, there are numerous barriers to the wide-scale adoption of such sequence databases, including access disparities for digital technologies, complex and restrictive intellectual property issues, and the simple fact that some crop species have received more attention from sequencing efforts. RTB crops may not be prominently featured in sequencing projects compared to higher-profile annual grain crops. This last fact may mean that providing information on relatives based on the evolutionary relationships among accessions may come later for these crops than for major economic crops.

Equally promising are the attempts to use genome-wide prediction to assess the value of gene bank accessions. These attempts require substantial DNA sequence information. El Hanafi et al. (2023) evaluated more than 10,000 accessions from two seed banks, the German Federal Ex Situ Genebank for Agricultural and Horticultural Crops (IPK), and the International Center for Agriculture Research in the Dry Areas (ICARDA) gene bank, along with several important traits. They found that for certain traits, genome-wide prediction might be useful as an alternative to phenomics-based projects to characterize seed bank accessions. Yu et al. (2016) and Fumia et al. (2023) found similar results in sorghum and mungbean, respectively.

*Gene editing enhances nutrition and safety in RTB crops:* Advances in gene editing, particularly the Clustered Regularly Interspaced Short Palindromic Repeats (CRISPR)/Cas9 system, have initiated a paradigm shift in the biofortification and nutritional enhancement of bulb, RTB storage organ crops. Unlike crossbreeding, which is often slow and limited by the high heterozygosity and complex ploidy of crops like potato, sweet potato and yams, CRISPR-mediated editing allows for the precise modification of specific metabolic pathways without the permanent integration of foreign DNA (Zhou et al. 2024). This transgene-free approach is particularly advantageous for RTB crops, as it can accelerate the development of cultivars with enhanced levels of essential micronutrients while potentially bypassing the stringent regulatory hurdles associated with transgenic crops.

A primary frontier in this field is the optimization of starch quality and the elimination of anti-nutritional factors—compounds that reduce the nutritional value of foods by interfering with the absorption or utilization of nutrients. For example, in potato, researchers have successfully targeted the *starch-branching enzyme* (*SBE2*) genes to develop high-amylose lines with significantly elevated resistant starch content, which offers major health benefits for managing diabetes and obesity (Batta et al. 2025). Potato resistant starch significantly improved glucose tolerance and insulin resistance, alleviated abnormal lipid metabolism and oxidative stress, reduced inflammation and pathological damage to liver and pancreas, and increased fecal short-chain fatty acid content and altered the gut microbiota in mouse model (Liu et al. 2024), while a pilot cross-over randomized controlled trial in human subjects (men and women at risk for type 2 diabetes) revealed that potatoes-enriched with resistant starch may have a favourable effect on carbohydrate metabolism, necessitating additional investigation in a larger sample on type 2 diabetes and weight management (Sanders et al. 2020). CRISPR/Cas9 has been used to knock out the *CYP79D1* and *CYP79D2* genes, which significantly reduces HCN content in the edited line, hence improving food safety in regions where cassava is a primary staple (Gomez et al. 2023).

Ongoing research focuses on improving the post-harvest shelf life and climate resilience of RTB crops to ensure their nutritional benefits are preserved from farm to table. In potatoes, gene editing is being used to decrease levels of steroidal glycoalkaloids—naturally occurring compounds that can be toxic at high concentrations (Nakayasu et al. 2018) and to reduce enzymatic browning (González et al. 2020). In cassava, efforts are underway to slow down postharvest physiological deterioration, which can rapidly reduce the quality and edibility of harvested roots (Ma et al. 2025).

To enhance iron (Fe) and zinc (Zn) bioavailability in cassava using CRISPR/Cas9, researchers employ a three-pronged metabolic strategy: increasing mineral uptake from the soil, enhancing the long-distance transport of minerals into the storage roots, and reducing anti-nutritional factors that inhibit human absorption. The main bottleneck in increasing cassava mineral density is the limited transport of metal ions from the leaves to the storage roots, which serve as the primary ‘sink’ tissues. By editing the promoter regions of genes involved in mineral transport, researchers can increase the sink strength of the roots, thereby drawing more Fe and Zn into the edible tissue (Narayanan et al. 2019). To prevent cellular toxicity from free iron, CRISPR can be used to precisely regulate the expression of Ferritin (*Fer1*), a protein that acts as a natural biological storage tank for iron. Editing regulatory elements to ensure root-specific accumulation of ferritin is a key strategy for biofortification (Narayanan et al. 2021). Another significant barrier to mineral bioavailability is the presence of phytic acid, a compound that acts as a potent chelator by binding to Fe and Zn in the digestive tract and forming insoluble complexes that humans cannot absorb (Dwivedi and Ortiz 2021). CRISPR/Cas9 can also be used to disrupt the *Inositol-3-Phosphate Synthase* (*IPS1*) and *Multidrug Resistance-associated Protein 5* (*MRP5*) genes; *IPS1* is the rate-limiting enzyme in phytic acid biosynthesis. By developing low-phytic acid cassava germplasm, the molar ratio of phytate to minerals can be reduced, thereby significantly increasing the fraction of absorbable iron and zinc (Gomez et al. 2023; Ma et al. 2025).

Recent advancements in multiplex editing (Cheng et al. 2025a, b)—a technique that enables the simultaneous modification of multiple genes—now allow for the concurrent enhancement of provitamin A carotenoids and minerals such as Fe and Zn by targeting regulatory genes involved in nutrient transport and sequestration, such as the *Orange* (*Or*) gene in sweet potato and cassava. Increasing provitamin A carotenoids through CRISPR knock-ins or gene editing may also improve iron bioavailability, as carotenoids can play a protective role in the gut or modulate the expression of mineral transporters. Multiplex CRISPR systems can target both the *Carotenoid Cleavage Dioxygenase 4* (*CCD4*) gene, to prevent pigment breakdown, and the *Nicotianamine Synthase* (*NAS*) gene, to facilitate mineral chelation and transport, simultaneously.

As CRISPR tools become more advanced, like base editing and prime editing, the ability to make single-nucleotide changes will enable precise modifications of vitamin biosynthetic enzymes in opportunity crops such as yams, taro, and turnip, which have received less attention from molecular research (Divya et al. 2024). These advancements, along with improved tissue culture-free delivery methods, have the potential to enhance the nutritional value of RTB crops for global food security. A CRISPR/Cas9-mediated genome editing protocol has been successfully established in onion, providing researchers opportunities to alter medically significant biomolecules in onions (Manikar et al. 2023). Currently, a genetic transformation platform or gene-editing technology system does not exist for garlic, as it is sexually sterile.

Antinutritional compounds, such as the cyanogenic glycosides linamarin and lotuaustralin in cassava, are key breeding targets. Gene editing of CYP79D1 and CYP79D2 with CRISPR/Cas9 allowed for the development of acyanogenic cassava in multiple investigations (Divya et al. 2024; Gomez et al. 2023; Juma et al. 2022). Reducing glycoalkaloid content in potato is a major breeding target. Gene editing solutions appear promising for glycoalkaloid reduction (Nakayasu et al. 2018), targeting the *St16DOX* gene has been effective. However, additional investigation will be required to assess the efficacy of *St16DOX* using a whole-plant system. Breeding targets in potato include keeping glycoalkaloids such as solanine below 20 mg 100 g^−1^ fresh weight, reducing acrylamide formation in processed potato products, reducing glycaemic index through the accumulation of resistant starch, and increasing mineral and vitamin content. Selection has been effective for both red and purple-fleshed potatoes, which contain anthocyanins and other antioxidants, while yellow and orange-fleshed potatoes contain carotenoids. Targeted mutagenesis using CRISPR/Cas9 can downregulate genes like polyphenol oxidase, resulting in improved processing quality in sweet potato. Low polyphenol content is preferred for the manufacturing of cassava chips.

Although gene editing protocols are increasingly being integrated into practical potato breeding programs—and, to a lesser extent, utilized in cassava for traits such as host plant resistance and reduced browning—their application across other RTB crops remains in its absolute infancy. This disparity stems from significant technical hurdles, including the lack of efficient, genotype-independent in vitro regeneration systems, difficulties in delivering CRISPR-Cas components to complex polyploid tissues, and a general scarcity of fully sequenced, high-quality reference genomes for neglected species like yam, taro, and garlic. Hence, while targeted genome modification is beginning to transform functional genomics and trait development in frontrunner crops, unlocking the full potential of gene editing for the broader spectrum of RTB crops will require foundational optimization of transformation pipelines and tissue culture protocols tailored to their unique vegetative growth habits.

*Biofortification*: It is estimated that 23 million farming households around the world now have access to biofortified seeds and are growing these crops to sustain their families and communities (HarvestPlus 2024). Biofortified crops are those that have been bred for enhanced nutritional properties but are familiar crops for the regions where they are grown. In this way, the biofortified version of the crop represents an improved variety of a familiar farm product that has customarily been consumed locally. Nutrients such as iron, zinc, or Vitamin A have been successfully enhanced in these cultivars bred through traditional methods, and in some cases using speed breeding techniques (Low and Carey 2020). The biofortified cultivars have been selected not only for enhanced nutrition, but for yield and other important agronomic factors, as well as for consumer acceptance. In many cases, farmers are part of the trialling and selection of improved biofortified crops. Farmers grow biofortified crops on their farms, but they may also sell excess produce to third parties, who may process or resell these foods. While several crops, such as cereal grains and legumes, have been biofortified, the primary RTB crops to be biofortified are sweet potato, cassava, potato, and yam.

Biofortification is particularly important in combatting hidden hunger. Hidden hunger is a phenomenon directly connected to poverty, in which billions of people around the world –primarily in Africa, Asia, and Latin America– are unable to afford a diet of nutritious food. Hidden hunger can result in severe health consequences for these populations, including weakened immune systems, stunted growth and development in infants and children, blindness, anaemia, and poor outcomes in pregnancy. Adoption of biofortified crops has been excellent in many regions of Africa, Asia, and Latin America. Because women are primarily responsible for RTB production in regions like South Saharan Africa, women and children are traditionally most at risk of micronutrient deficiencies. Organizations working on biofortified crops, therefore, prioritized women’s access to these technologies, and special efforts have been made to understand gender-responsive programs to reach nutrition goals.

A large portion of the work devoted to biofortification is credited to organizations like HarvestPlus, a global not-for-profit program, which works across the CGIAR system as part of the International Food Policy Research Institute. The International Potato Center, based in Peru, has also played a pivotal role in biofortifying crops. These organizations partner with government-funded plant breeding programs to develop improved germplasm. They partner with companies that focus on the multiplication of seed, vines, or cuttings to more efficiently distribute the cultivars, as well as processors who increase the value chain with consumable products. The primary RTB crops targeted for biofortification thus far have been sweet potato, cassava, yam, and potato. Perhaps the most successful biofortified crop, sweet potato, was bred to have higher levels of provitamin A carotenoids that translate into improved Vitamin A status for consumers. A “biofortified” sweet potato is a conventionally bred cultivar bred to significantly increase its micronutrient content to combat nutritional deficiencies in target populations (Gruneberg et al. 2015). To officially qualify as biofortified, a sweet potato must independently achieve specific minimum nutritional thresholds designed to supply a major portion of daily requirements (such as meeting at least 50% of the target nutritional needs for women and children). It must achieve a minimum target level providing at least 50% of the recommended daily allowance or estimated requirements, which are often expressed in breeding targets as an increment of roughly 70 ppm of provitamin A carotenoids. Moreover, a “biofortified” sweet potato should show a mineral density threshold around 60 ppm for iron, or 40 ppm for zinc. Yellow-fleshed cassava cultivars with improved provitamin A carotenoids have been released, including some that are transgenic (Eyinla et al. 2019; Rodríguez et al. 2025). Cassava germplasm includes sources with high levels of provitamin A carotenoids, such as the cultivar ‘Amarelinha do Amapa’, with β-carotene levels up to 50-fold higher than other cultivars. The main challenge for biofortification in cassava breeding is the negative correlation between high provitamin A carotenoids and starch content. Since cassava is such an important source of starch in the diet, the trade-off between high carotenoid levels and low starch is a potentially problematic area.

Potatoes with increased iron and zinc content, as well as higher levels of provitamin A carotenoids, have been developed. Biofortified yam cultivars with higher mineral and vitamin levels have also been developed. One of the challenges of the biofortification paradigm is that some traditionally preferred cultivars are lower in nutrient status. The yellow yam, *Dioscorea cayennensis*, has higher carotenoid content than the white yam, *Dioscorea rotundata*, which is preferred by farmers and consumers. Some yam cultivars from the species *alata* and *trifida* contain anthocyanins, which may translate into potential health benefits.

Substantial evidence points toward the success of biofortification programs. de Brauw et al. (2018) conducted randomized field experiments in Mozambique and Uganda on orange-flesh sweet potato (OFSP), among the most successful biofortified crops. Increased adoption of OFSP by farmers of 61% to 68% exposed to the project, with an accompanying doubling of Vitamin A intake by children consuming those cultivars, were measured. Interestingly, increased vitamin A intakes were largely explained by adoption rather than by nutrition knowledge gained. Strong adoption of Vitamin A-enriched cassava cultivars was measured in four Nigerian states, with some 21% of cassava-focused land area devoted to these cultivars.

Iron absorption was measured from an iron-biofortified yellow-fleshed potato cultivar and compared to a non-biofortified yellow-fleshed potato cultivar. This unique intervention study made use of labelled 58Fe sulphate (biofortified) or 57Fe sulphate (non-fortified) for the different potato meals, and iron absorption was estimated from the iron isotopic composition in erythrocytes. Total iron absorption from the iron-biofortified potato was almost 46% higher than from the non-biofortified potato, suggesting the promise of iron biofortification in iron-deficient women using potato that had been biofortified using conventional breeding.

Liria-Dominguez et al. (2023) studied the potential of biofortified potatoes to reduce zinc deficiency due to their lower levels of phytate. Using stable isotopes to label the two different potato types, they followed 37 women who consumed 500 g of either biofortified or regular potatoes twice daily. Zinc absorption was measured in urine. They found that biofortified potatoes provide more absorbable zinc than regular potatoes.

Accelerated breeding schemes have been developed for biofortified crops. Low and Carey (2020) described how breeders made 9 copies of each seedling in a screenhouse and then used these at 3 different sites for the first observational trial. In this scheme, there are multiple sites in the first year, and at least one of those sites is a stressful environment. This approach was used successfully by Maria Andrade and her team in the development of superior OFSP cultivars. The 2016 World Food Prize was jointly awarded to her and Robert Mwanga, Jan Low, and Howarth Bouis for their pioneering leadership in biofortification to combat global hunger and malnutrition. Andrade and Mwanga were recognized for successfully breeding vitamin A-enriched, orange-fleshed sweet potato cultivars, while Low structured the essential nutrition research and adoption programs that enabled millions of households across Africa to grow and consume them. Concurrently, Bouis pioneered a multi-institutional global strategy at CGIAR-led HarvestPlus to integrate biofortification across multiple staple crops, permanently transforming nutritional security worldwide.

Iron and zinc deficiencies are common in West African populations. Narayanan et al. (2019) overexpressed a vacuolar iron transporter in cassava to improve iron levels from 3- to 7-fold in transgenic cassava roots. Plants modified with an iron transporter and ferretin gene accumulated iron 7–18 times higher and zinc 3–10 times higher than non-transgenic controls. Further evaluations revealed high levels of bioavailability for both iron and zinc from these transgenic sources.

Attempts have been made to use genetic engineering in biofortification programs. Overexpression of IbMYB1, an important regulator of anthocyanin biosynthesis in the storage roots, increased the antioxidant capacity of OFSP (Park et al. 2015). Co-expression of the *crtB* gene and a gene for 1-deoxy-D-xylulose 5-phosphate synthase (DXS) from *Arabidopsis* resulted in carotenoid accumulation of 40–60 µg/g DW in cassava storage roots compared with a non-transformed control. Expression of a bacterial *crtB* gene for phytoene synthase (PSY) alone resulted in a carotenoid concentration of ~25 µg/g DW in the roots (Sayre et al. 2011).

*Metabolic engineering of pigment biosynthesis in root and tuber crops for improved colour and nutrition:* Metabolic engineering, which involves the targeted modification of metabolic pathways to alter the chemical composition of organisms, has emerged as a cornerstone in the diversification of RTB crop germplasm. This approach shifts the focus from basic caloric security to the development of functional foods rich in bioactive pigments. The vibrant colours in crops such as potato, sweet potato, cassava, and carrot are primarily derived from two major metabolic pathways: the phenylpropanoid pathway, which produces anthocyanins (responsible for purples and blues), and the isoprenoid pathway, which generates carotenoids (responsible for yellows and oranges). By precisely manipulating the metabolic flux through these pathways, plant breeders are now able to colour previously white-fleshed germplasm, resulting in increased levels of antioxidants and provitamin A in the crops, for example, potato tubers with high β-carotene and lutein (Ducreux et al. 2005).

In the realm of anthocyanin engineering, the focus centres on the regulation of late biosynthetic genes through the MYB-bHLH-WD40 (MBW) complex. In potato and sweet potato, the overexpression of specific R2R3-MYB transcription factors has been shown to simultaneously activate multiple genes, such as *Dihydroflavonol 4-reductase (DFR)* and *Anthocyanidin synthase (ANS)*, resulting in deep purple flesh with high antioxidant capacity (Singh et al. 2022c). Conversely, carotenoid engineering often targets “metabolic sinks” rather than just biosynthetic enzymes. A potential breakthrough in cassava and potato breeding involves the manipulation of the *Orange* (*Or*) gene, which triggers the formation of chromoplasts, specialized organelles that sequester and stabilize carotenoids. This "sink-strength" enhancement prevents the degradation of pigments, leading to the development of deep-orange germplasm with significantly higher carotene retention (Abincha et al. 2024; Ma et al. 2025). Deng et al. (2023) bred colourful carrot germplasm by using metabolic engineering of betalain pigments. Such a germplasm offers a colourful and functional vegetable with enhanced levels of bioactive pigments.

Recent advances in multi-gene “stacking” and CRISPR-mediated base editing are enabling the fine-tuning of metabolic pathways, thereby facilitating the development of novel colour profiles such as pink-fleshed potatoes or high-lycopene carrots. Moreover, metabolic profiling may be employed to ensure that redirecting carbon toward pigment production does not adversely affect other essential traits, including starch quality or yield (Kumar et al. 2017; Divya et al. 2024). As the engineered germplasm moves toward field evaluation, it may provide essential micronutrients in a visually appealing and culturally acceptable form.

### Assessing sensory perception and consumers’ acceptance of nutritionally enhanced RTB crop germplasm

Sensory attributes – taste, texture, odour, and appearance – are the main drivers of consumer acceptance in many RTB crops in the Global South. As such, these traits should be considered breeding targets alongside yield and nutritional composition, rather than being overlooked in breeding objectives. Sensory quality evaluations of nutritionally enhanced RTB germplasm remain relatively limited, especially when compared to seed crops like corn, beans, and small grains. For example, information on the many textural traits is limited, particularly because these traits are difficult to quantify and have not been extensively studied; in fact, data are only available for up to 34% of the texture trait measurements across yams, sweet potato, cassava, and potato. (Harris et al. 2015). Processing and cooking quality are also important, as RTB crops are used in regionally specific cultural contexts. Consumer acceptance is therefore complex, and the most successful integration often happens when the nutritional enhancement is invisible, as it aligns with traditional sensory expectations, although challenges still arise with technology acceptance. Thus, increasing the nutrient concentration alone does not necessarily translate to nutritional impact if the cultivar is not being purchased and prepared. As a result, successful breeding efforts must extend beyond yield to incorporate traits that align with consumer expectations and familiarity.

Pro-vitamin A sweet potato is the most studied of the biofortified RTB crops for sensory attributes and is generally well accepted (Huey et al. 2023). Rosero et al. (2022) found that people were likely to purchase the variety that they rated highest, with orange flesh sweet potato (OFSP) cultivars Ambarina and Aurora receiving top scores in all sensory characteristics, such as low fibrosity, thus can be said to have very high acceptability that could be transformed into purchasing decisions. OFSP was also highly accepted by mothers in Tanzania and India, where it was incorporated into cultural dishes such as chutney, rasam, thick shake, barfi, payasam, which were consumed readily. A mother’s acceptance often leads to children’s liking, since they are more likely to purchase and prepare (Tomlins et al. 2007; Gannon et al. 2019). Additionally, OFSP was the most preferred across price levels, indicating consumer preference over white and yellow cultivars regardless of cost (Etumnu 2016).

Similar results have been reported in cassava, in which biofortified cultivars, yellow in colour and lower dry matter, are affecting texture. Both children and caretakers detected significant differences and preferred the provitamin A cassava for its attractive colour, softer texture, and sweet taste. Notably, cassava was not regularly consumed in this community, which may have increased openness to new cultivars (Talsma et al. 2013).

**Fig. 1:**
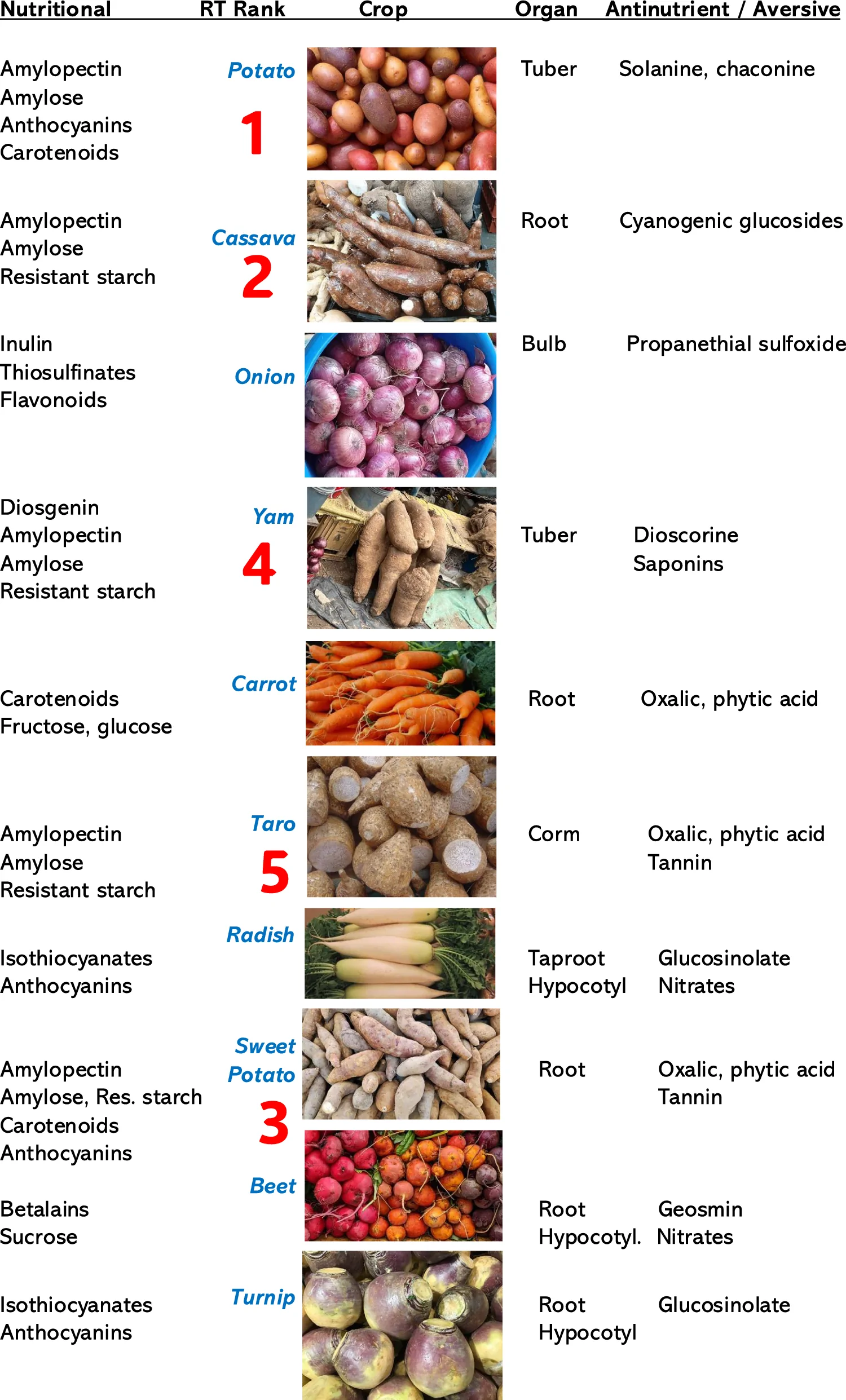
Primary recognized nutritional component(s), global rank for production, primary organ(s) consumed, and antinutrient / aversive compound(s) in ten of the world’s major root, tuber, and bulb (RTB) crops. Antinutrients may reduce the absorption of other nutrients in the diet or in the vegetable itself; aversive compounds may deter people from consumption of the vegetable. Images from Wikimedia Commons via Creative Commons license: potato (public domain), cassava (Emoke Denes), Onion (Natashadonoh), yam (wilhelmmarvel), carrot, (domdomegg), corm (public domain), radish (Chris 73), sweet potato (Melsj), beet (Eden, Janine and Jim), turnip (Mike Mozart)

Contrastingly, food neophobia is the tendency to avoid novel or unfamiliar foods, a shared characteristic of humans. A survey conducted in China reported that invisible nutritional changes may trigger nutrition-related food neophobia, while visible nutrition traits may also elicit sensory-food neophobia (Razzaq et al. 2021). However, fear can often be reduced with information sharing. Providing consumers with nutritional and health information is a crucial factor in acceptance (Etumnu 2016), and increased awareness has been shown to improve cultural acceptability of biofortified cassava by addressing concerns about colour, taste, texture, and its role in reducing vitamin A deficiency (Talsma et al. 2013). Engaging with farmers and farmer groups, processors, and marketers who are acquainted with the limitations associated with cassava production and utilization, to share a basic understanding of the benefits of transgenic technology, is essential in achieving product acceptance and adoption (Okwuonu et al. 2021). Incorporating sensory and processing traits into breeding objectives may help to ensure that nutritional improvements result in cultivars that are accepted and ultimately consumed (Fig. 1).

## Conclusion

Root, tuber, and bulb (RTB) crops (Figure 1) are vital for global food security and nutrition across Africa, Asia, and Latin America, yet a persistent gap remains between the vast accumulation of genomic data and the successful delivery of improved cultivars. While research demonstrates ample genetic diversity and foundational insights into trait inheritance—highlighted by successful provitamin A-enriched cassava, orange-fleshed sweet potato, and biofortified potatoes—genomic and nutritional discoveries have not yet translated consistently into applied breeding due to fundamental bottlenecks in validation, quantitative genetics, and crop-specific constraints. A few crops are better researched than others. Hence, more resources are needed for under-researched crops, with a focus on accurate ploidy determination, phenomics, diversity panels, high-density markers, predictive breeding, reference genomes/pangenomes, and training populations.

The field currently stands at a crossroads where massive repositories of putative genes, SNPs, and QTL largely lack functional validation across diverse genetic backgrounds and environments. Furthermore, below-ground phenotyping remains a severe constraint, creating a wide genome-to-phenome gap that limits the power of genomic selection. Overcoming these limitations requires a decisive shift toward answering core research questions regarding how putative genes and genetic variants identified in diversity panels can be functionally validated across variable genetic backgrounds and complex environments. Researchers must also determine the precise physiological and molecular mechanisms governing nutrient absorption, transport, and storage, alongside the source-sink dynamics that dictate storage organ development. Additionally, the field must address how prediction accuracy and genetic gain can be maximized through advanced multi-trait genomic selection while successfully navigating pleiotropic trade-offs between productivity, nutritional traits, and consumer acceptance, as well as the extent to which alleles maintain their stability across varying ploidy levels, environments, and genotype-by-environment interactions.

## Data Availability

No datasets were generated or analysed during the current study.

## References

[CR1] Abah SP, Mbe JO, Dzidzienyo DK, Njoku D, Onyeka J, Danquah EY, Offei SK, Kulakow P, Egesi CN (2024) Determination of genomic regions associated with early storage root formation and bulking in cassava. Front Plant Sci 15:1391452. 10.3389/fpls.2024.1391452 PMC11233741 38988637

[CR2] Abincha W, Dzidzienyo DK, Wesonga LN, Mwimali M, Ozimati A, Kayondo IS, Ofori K, Tongoona P, Kivuva BM (2024) Enhancing nutrition: a review of provitamin A carotenoid cassava breeding initiatives in East Africa. Adv Agric. 10.1155/aia/9937876

[CR3] Agaba R, Tukamuhabwa P, Rubaihayo P (2016) Genetic variability for yield and nutritional quality in yam bean (*Pachyrhizus sp.*). HortScience 51:1079–1086. 10.21273/HORTSCI10686-16

[CR4] Aghogho CI, Kayondo SI, Eleblu SJY, Ige A, Asante I, Offei SK, Parkes E, Egesi C, Mbanjo EGN, Shah T, Kulakow P, Rabbi IY (2024) Genome-wide association study for yield and quality of granulated cassava processed product. Plant Genome 17:e20469. 10.1002/tpg2.20469 PMC12806961 38880944

[CR5] Agrama HA, Yan W, Lee F, Fjellstrom R, Chen M-H, Jie M, McClung A (2009) Genetic assessment of a mini-core subset developed from the USDA rice genebank. Crop Sci 49(4):1336–1346. 10.2135/cropsci2008.06.0551

[CR6] Agre PA, Darkwa K, Kohtaro I, Matsumoto R, Asfaw A (2025) Genome wide association studies in yam reveal the challenge of high heterozygosity. Sci Rep 15:26929. 10.1038/s41598-025-10344-z PMC12289929 40707498

[CR7] Ahmad D, Cawood M, Iqbal Q, Arfio A, Batool A, Tariq RMS, Azam M, Akhtar S (2019) Phytochemicals in *Daucus carota* and their health benefits—review article. Foods 8:424. 10.3390/foods8090424 PMC6770766 31546950

[CR8] Ahmad D, Zhang L, Gao Y, Hu Y, Deng B, Tong C, Bao J (2025) Genotypic diversity and genome-wide association study of phosphorus content, amylose content, pasting, and textural properties of potato starch. Carbohydr Polym 367:123956. 10.1016/j.carbpol.2025.123956 40817514

[CR9] Ainsworth EA, Gillespie KM (2007) Estimation of total phenolic content and other oxidation substrates in plant tissues using Folin-Ciocalteu reagent. Nat Protoc 2:875–877. 10.1038/nprot.2007.102 17446889

[CR10] Ajomiwe N, Boland M, Phongthai S, Bagiyal M, Singh J, Kaur L (2024) Protein nutrition: understanding structure, digestibility, and bioavailability for optimal health. Foods 13:1771. 10.3390/foods13111771 PMC11171741 38890999

[CR11] Akdemir D, Beavis W, Fritsche-Neto R, Singh AK, Isidro-Sánchez J (2019) Multi-objective optimized genomic breeding strategies for sustainable food improvement. Heredity (Edinb) 122:672–683. 10.1038/s41437-018-0147-1 PMC6461918 30262841

[CR12] Alamu EO, Nuwamanya E, Cornet D, Meghar K, Adesokan M, Tran T, Belalcazar J, Desfontaines L, Davrieux F (2020) Near-infrared spectroscopy applications for high-throughput phenotyping for cassava and yam: a review. Int J Food Sci Technol 56:1491–1501. 10.1111/ijfs.14773 PMC7984172 33776247

[CR404] Vega Alfaro A, Scully L, Dixon Goldman IL (2025) Phenomics to improve genebank value and utility *In* P. M. Priyadarshan, R. Ortiz (eds.), Plant Breeding 2050, Springer Nature Singapore DOI.org/10.1007/978-981-95-0583-8_11

[CR13] Alfurayhi R, Huang L, Brandt K (2023) Pathways affected by falcarinol-type polyacetylenes and implications for their anti-inflammatory function and potential in cancer chemoprevention. Foods 12:1192. 10.3390/foods12061192 PMC10048309 36981118

[CR14] Alicandri E, De Santis D, Rea R, Ciaffi M (2025) Genetic and phenotypic diversity and evaluation of total phenolics and antioxidant properties of garlic landraces from Lazio Region (Central Italy): “Aglio Rosso di Proceno” and “Aglio Rosso di Castelliri.” Plants (Basel) 14:1189. 10.3390/plants14081189 PMC12030451 40284083

[CR15] Allem AC (2002) The origins of cassava. In: Hillocks RJ, Thresh JM, Bellotti AC (eds) Cassava: biology, production and utilization. CABI Publishing, United Kingdom, pp 1–16

[CR16] Amir A, Sharangi AB, Bal S, Upadhyay TK, Khan MS, Ahmad I, Alabdallah NM, Saeed M, Thapa U (2023) Genetic variability and diversity in red onion (Allium cepa L.) genotypes: Elucidating morpho-horticultural and quality perspectives. Horticulturae 9(1):1005

[CR17] Andersson M, Turesson H, Olsson N, Fält A-S, Ohlsson P, Gonzalez MN, Samuelsson M. Hofvander P (2018) Genome editing in potato via CRISPR-Cas9 ribonucleoprotein delivery. Physiol Plantarum, 164:378–384. 10.1111/ppl.12731 29572864

[CR18] Andrade MI, Ricardo J, Naico A, Alvaro A, Makunde GS, Low J, Ortiz R, Gruneberg WJ (2017) Release of orange-fleshed sweetpotato (*Ipomoea batatas* [l.] Lam.) cultivars in Mozambique through an accelerated breeding scheme. J Agric Sci 155:919–929. 10.1017/S002185961600099X

[CR482] Anglin NL, Robles RR, Rossel G, Alagon R, Panta A, Jarret RL, Manrique N, Ellis D (2021) Genetic identity, diversity, and population structure of CIP's sweet potato (I.batatas) germplasm collection. Front Plant Sci 12:660012. 10.3389/fpls.2021.660012 PMC8589021 34777403

[CR19] Anthon GE, Barrett DM (2003) Modified method for the determination of pyruvic acid with dinitrophenylhydrazine in the assessment of onion pungency. J Sci Food Agric 83:1210–1213

[CR20] AOAC (1980) Official method of analysis, 13th addition Ed. Association of official analytical Chemists, Arlington, VA, USA

[CR22] AOAC (2005). Official Methods of Analysis of AOAC International, 18th ed.; Horwitz,W., Latimer, G., Eds.; MD: AOAC International: Gaithersburg, MD, USA, 2005.

[CR21] AOAC (2012) Official methods of analysis of the Association of Official Analytical Chemists (AOAC), 19th edn. AOAC International, AOAC, Gaithersburg, MD, USA

[CR23] AOAC (2016). Official Methods of Analysis of AOAC International, 20th ed.; Horwitz, W., Latimer, G., Eds.; MD: AOAC International: Gaithersburg, MD, USA, 2016.

[CR24] Arcos LPA, Garcés DMS, Guaña JLM, García-Segovia P, Martínez-Monzó J, Igual M (2025) Current situation of Andean tubers and tuberous roots: ancestral, medicinal, and technological potential. Cogent Food Agric 11:2505008. 10.1080/23311932.2025.2505008

[CR25] Arifuzzaman M, Oladzadabbasabadi A, McClean P, Rahman M (2019) Shovelomics for phenotyping root architectural traits of rapeseed/canola (*Brassica napus* L.) and genome-wide association mapping. Mol Genet Genomics 294:985–1000. 10.1007/s00438-019-01563-x 30968249

[CR26] Arnau G, Desfontaines L, Ehounou AE, Marie-Magdeleine C, Kouakou AM, Leinster J, Nudol E, Maledon E, Chair H (2024) Quantitative trait loci and candidate genes for physico-chemical traits related to tuber quality in greater yam (*Dioscorea alata* L.). J Sci Food Agric 104:4872–4879. 10.1002/jsfa.12822 37400964

[CR27] Arvouet-Grand A, Vennat B, Pourrat A, Legret P (1994) Standardisation d’un extrait de propolis et identification des principaux constituants. Journal De Pharmacie De Belgique Catalogue En Ligne 49:462–468 7884635

[CR28] Atanbori J, Montoya-P EM, Selvaraj MG, French AP, Pridmore TP (2019) Convolutional neural net-based cassava storage root counting using real and synthetic images. Front Plant Sci 10:1516. 10.3389/fpls.2019.01516 PMC6888701 31850020

[CR29] Aubry S (2023) Genebanking plant genetic resources in the post genomic era. Agric Hum Values 40:961–971. 10.1007/s10460-023-10417-7

[CR30] Avnee SS, Chaudhary DR, Jhorar P, Rana RS (2023) Biofortification: an approach to eradicate micronutrient deficiency. Front Nutr 10:1233070. 10.3389/fnut.2023.1233070 PMC10543656 37789898

[CR31] Awika JM, Rooney LW, Waniska RD (2005) Anthocyanins from black sorghum and their antioxidant properties. Food Chem. 10.1016/j.foodchem.2004.03.058

[CR32] Bahjat NM, Yidiz M, Nadeem MA, Morales A, Wohlfeiler J, Baloch FS, Tunçtürk M, Koçak M, Chung YS, Grzebelus D, Sadik G, Kuzğun C, Cavagnaro PF (2025) Population structure, genetic diversity, and GWAS analyses with GBS-derived SNPs and silicodart markers unveil genetic potential for breeding and candidate genes for agronomic and root quality traits in an international sugar beet germplasm collection. BMC Plant Biol 25:523. 10.1186/s12870-025-06525-7 PMC12044756 40307730

[CR33] Baker H, Frank O, Angelis BD, Feingold SE (1980) Plasma tocopherol in man at various times after ingesting free or acetylated tocopherol. Nutr Rep Int 21:531–536

[CR34] Balakrishna A, Joshi M, Gupta S, Rani MP, Srivastava J, Nain P, Varshney A (2024) Dissecting the natural phytochemical diversity of carrot roots with its colour using high performance liquid chromatography and UV–visible spectrophotometry. Heliyon 10:e35918 10.1016/j.heliyon.2024.e35918 PMC11365394 39220899

[CR35] Bali S, Robinson BR, Sathuvalli V, Bamberg J, Goyer A (2018) Single nucleotide polymorphism (SNP) markers associated with high folate content in wild potato species. PLoS ONE 13:e0193415 10.1371/journal.pone.0193415 PMC5825101 29474475

[CR36] Banga O, De Bruyn JW, Smeets L (1955) Selection of carrots for carotene content part II: sub-normal content at low temperature. Euphytica 4:183–189. 10.1007/BF00039644

[CR37] Bannoud F, Carvajal S, Ellison S, Senalik D, Talquenca SG, Lorozzo M, Simon PW, Cavagnaro PF (2021) Genetic and transcription profile analysis of tissue-specific anthocyanin pigmentation in carrot root phloem. Genes (Basel) 12:1464. 10.3390/genes12101464 PMC8535259 34680859

[CR38] Bararyenya A, Olukolu BA, Tukamuhabwa P, Grüneberg WJ, Ekaya W, Low J, Ochwo-Ssemakula M, Odong TL, Talwana H, Badji A, Kyalo M, Nasser Y, Gemenet D, Kitavi M, Mwanga ROM (2020) Genome-wide association study identified candidate genes controlling continuous storage root formation and bulking in hexaploidy sweetpotato. BMC Plant Biol 20:3. 10.1186/s12870-019-2217-9 PMC6941292 31898489

[CR40] Barba AO, Hurtado MC, Mata MS, Ruiz VF, De Tejada MLS (2006) Application of a UV–vis detection-HPLC method for a rapid determination of lycopene and β-carotene in vegetables. Food Chem 95:328–336

[CR39] Barba F, Esteve MJ, Tedeschi P, Brandolini V, Frigola AA (2013) A comparative study of the analysis of antioxidant activities of liquid foods employing spectrophotometric, fluorometric, and chemiluminescent methods. Food Anal Methods 6:317–327. 10.1007/s12161-012-9441-3

[CR41] Barbano DM, Clark JL, Dunham CE, Flemin RJ (1990) Kjeldahl method for determination of total nitrogen content of milk: Collaborative study. J Assoc off Anal Chem 73:849–859. 10.1093/jaoac/73.6.849

[CR42] Barboza K, Salinas MC, Acuña CV, Bannoud F, Beretta V, García-Lampasona S, Burba JL, Galmarini CR, Cavagnaro PF (2020) Assessment of genetic diversity and population structure in a garlic (*Allium sativum* L.) germplasm collection varying in bulb content of pyruvate, phenolics, and solids. Sci Hortic 261:108900. 10.1016/j.scienta.2019.108900

[CR43] Batta S, Siddappa S, Sharma N, Singh R, Gupta R, Kumar D, Singh B, Thakur AK (2025) CRISPR-Cas9 mediated editing of *starch branching enzyme*, *SBE2* gene in potato for enhanced resistant starch for health benefits. Front Genome Ed 7:1686412. 10.3389/fgeed.2025 PMC12689564 41384050

[CR44] Bedir AS, Almasri RS, Azar YO, Elnady RE, Al Raish SM (2025) Exploring the therapeutic potential of *Allium cepa* and *Allium sativum* extracts: Current strategies, emerging applications, and sustainability utilization. Biology (Basel) 14:1088. 10.3390/biology14081088 PMC12383551 40906415

[CR45] Behera TK, Singh S (2019) Advances in vegetable breeding for nutraceuticals and quality traits. Indian J Genet Plant Breed 79 (Sup 1):216–226. 10.31742/ijgpb.79s.1.12

[CR46] Bennett RN, Mellon FA, Kroon PA (2004) Screening crucifer seeds as sources of specific intact glucosinolates using ion-pair high performance liquid chromatography negative ion electrospray mass spectrometry. J Agric Food Chem 52:428–438 10.1021/jf030530p 14759128

[CR47] Benton-Jones J (1989) Plant analysis techniques. Benton-Jones Laboratories, Georgia, p 24

[CR48] Benzie IFF, Strain JJ (1996) The ferric reducing ability of plasma (FRAP) as a measure of “antioxidant power”: the FRAP assay. Anal Biochem 239:70–76 10.1006/abio.1996.0292 8660627

[CR49] Berdugo-Cely JA, Céron-Lasso MDS, Yockteng R (2023) Phenotypic and molecular analyses in diploid and tetraploid genotypes of *Solanum tuberosum* L. reveal promising genotypes and candidate genes associated with phenolic compounds, ascorbic acid contents, and antioxidant activity. Front Plant Sci. 13:1007104. 10.3389/fpls.2022.1007104 PMC9889998 36743552

[CR50] Blando F, Marchello S, Maiorano G, Durante M, Signore A, Laus MN, Soccio M, Mita G (2021) Bioactive compounds and antioxidant capacity in anthocyanin-rich carrots: a comparison between the black carrot and the Apulian landrace “Polignano” carrot. Plants 10:564. 10.3390/plants10030564 PMC8002536 33802658

[CR51] Blois M (1958) Antioxidant determinations by the use of a stable free radical. Nature 181:1199–1200. 10.1038/1811199a0

[CR52] Bloom ZA, Larsen JC, Pena Martinez EE, Williams CM, Jones DS, Kudenov MW (2025) High-throughput classification and quantification of skinning phenotype in sweet potatoes. Plant Phenome J 8:e70023. 10.1002/ppj2.70023

[CR53] Bokanga M (1994) Distribution of cyanogenic potential in cassava germplasm. Acta Hortic 375:117–124. 10.17660/ActaHortic.1994.375.9

[CR54] Bonasia A, Conversa G, Lazzizera C, Elia A (2013) Pre-harvest nitrogen and azoxystrobin application enhances postharvest shelf-life in butterhead lettuce. Postharvest Biol Technol 85:67–76

[CR55] Bonasia A, Conversa G, Lazzizera C, Loizzo P, Gambacorta G, Elia A (2020) Evaluation of garlic landraces from Foggia province (Puglia Region; Italy). Foods 9:850. 10.3390/foods9070850 PMC7404550 32610546

[CR56] Borlinghaus J, Albrecht F, Gruhlke MCH, Nwachukwu ID, Slusarenko AJ (2014) Allicin: chemistry and biological properties. Molecules 19:12591–12618. 10.3390/molecules190812591 PMC6271412 25153873

[CR57] Bouis H, Foley J, Lividini K, Jumrani J, Reinke R, Van Der Straeten D, Zagado R, Boy E, Brown LR, Mudyahoto B, Alioma R, Hussain M, Pfeiffer WH (2024) Biofortification: future challenges for a newly emerging technology to improve nutrition security sustainably. Curr Dev Nutr 8:104478. 10.1016/j.cdnut.2024.104478 PMC11635736 39668944

[CR58] Bowers RR, Slonecki TJ, Olukolu BA, Yencho CG, Wadl PA (2024a) Genome-wide association study of sweet potato storage root traits using GWASpoly, a gene dosage-sensitive model. Int J Mol Sci 25:11727. 10.3390/ijms252111727 PMC11546673 39519288

[CR59] Boyles MJ, Wrolstad RE (1993) Anthocyanin composition of red raspberry juice: influences of cultivar, processing, and environmental factors. J Food Sci 58:1135–1141

[CR60] Bradford MM (1976) A rapid and sensitive method for the quantitation of microgram quantities of protein utilizing the principle of protein-dye binding. Anal Biochem 72:248–254 10.1016/0003-2697(76)90527-3 942051

[CR61] Bradstreet RS (1965) The Kjeldahl method for organic nitrogen. Academic Press

[CR62] Brainard SH, Bustamante JA, Dawson JC, Spalding EP, Goldman IL (2021a) A digital image-based phenotyping platform for analyzing root shape attributes in carrot. Front Plant Sci 12:690031. 10.3389/fpls.2021.690031 PMC8244657 34220912

[CR63] Brainard SH, Ellison SL, Simon PW, Dawson JC, Goldman IL (2021b) Genetic characterization of carrot root shape and size using genome-wide association analysis and genomic-estimated breeding values. Theor Appl Genet 135:605–622. 10.1007/s00122-021-03988-8 PMC8866378 34782932

[CR64] Brand-Williams W, Cuvelier M-E, Berset C (1995) Use of a free radical method to evaluate antioxidant activity. LWT-Food Sci Technol 28:25–30. 10.1016/S0023-6438(95)80008-5

[CR65] Braniša J, Jenisová Z, Porubská M, Jomová K, Valko M (2014) Spectrophotometric determination of chlorophylls and carotenoids: an effect of sonication and sample processing. J Microbiol Biotechnol Food Sci 3:61–64

[CR66] Bredeson JV, Lyons JB, Prochnik SE, Wu GA, Ha CM, Edsinger-Gonzales E, Grimwood J, Schmutz J, Rabbi I, Egesi C, Nauluvula P, Lebot V, Ndunguru J, Mkamilo G, Bart RS, Setter TL, Gleadow RM, Kulakow P, Ferguson ME, Rounsley S, Rokhsar DS (2016) Sequencing wild and cultivated cassava and related species reveals extensive interspecific hybridization and genetic diversity. Nat Biotechnol 34:5. 10.1038/nbt.3535 27088722

[CR67] Breiteneder H, Ebner C (2000) Molecular and biochemical classification of plant-derived food allergens. J Allergy Clin Immunol 106:27–36. 10.1067/mai.2000.106929 10887301

[CR68] Breiteneder H, Radauer C (2004) A classification of plant food allergens. J Allergy Clin Immunol 113:821–830. 10.1016/j.jaci.2004.01.779 15131562

[CR69] Brown PD, Tokuhisa JG, Reichelt M, Gershenzon J (2003) Variation of glucosinolate accumulation among different organs and developmental stages of *Arabidopsis thaliana*. Phytochemistry 62:471–481. 10.1016/S0031-9422(02)00549-6 12620360

[CR70] Bull SE, Seung D, Chanez C, Mehta D, Kuon J-E, Truernit E, Hochmuth, A., Zurkirchen, I., Zeeman, S.C., Gruissem, W., Vanderschuren, H. (2018). Accelerated ex situ breeding of GBSS- and PTST1-edited cassava for modified starch. Sci Adv 4:eaat6086. 10.1126/sciadv.aat6086 PMC6124905 30191180

[CR71] Burns RE (1971) Method for Estimation of tannin in grain sorghum 1. Agron J 63, 511–512. h t t p s : / / d o i . o r g / 1 0 . 2 1 3 4 / a g r o n j 1 9 7 1 . 0 0 0 2 1 9 6 2 0 0 6 3 0 0 0 3 0 0 5 0 x

[CR72] Cai Z, Cai Z, Huang J, Wang A, Ntambiyukuri A, Chen B, Zheng G, Li H, Huang Y, Zhan J, Xiao D, He L (2022) Transcriptomic analysis of tuberous root in two sweet potato varieties reveals the important genes and regulatory pathways in tuberous root development. BMC Genomics 2:473. 10.1186/s12864-022-08670-x PMC9235109 35761189

[CR73] Calvo-Gómez O, Morales-López J, López MG (2004) Solid-phase microextraction-gas chromatographic-mass spectrometric analysis of garlic oil obtained by hydrodistillation. J Chromatogr A 1036:91–93. 10.1016/j.chroma.2004.02.072 15139418

[CR74] Cantliffe DJ (1973) Nitrate accumulation in table beets and spinach as affected by nitrogen, phosphorus, and potassium nutrition and light intensity. Agron J 65:563–565. 10.2134/agronj1973.00021962006500040012x

[CR75] Cao T, Wang S, Ali A, Shan N, Sun J, Chen X, Wang P, Zhu Q, Xiao Y, Luo S, Zhou Q, Huan Y (2023) Transcriptome and metabolome analysis reveals the potential mechanism of tuber dynamic development in yam (D*ioscorea polystachya* Turcz.) LWT 181:114764. 10.1016/j.lwt.2023.114764

[CR76] Carluccio AV, David LC, Claußen J, Sulley M, Adeoti SR, Abdulsalam T, Gerth S, Zeeman SC, Gisel A, Stavolone L (2022) Set up from the beginning: the origin and early development of cassava storage roots. Plant Cell Environ 45:1779–1795. 10.1111/pce.14300 PMC9314696 35229892

[CR77] Carvajal-Yepes M, Ospina JA, Aranzales E, Velez-Tobon M, Correa Abondano M, Manrique-Carpintero NC, Wenzl P (2024) Identifying genetically redundant accessions in the world’s largest cassava collection. Front Plant Sci 14:1338377. 10.3389/fpls.2023.1338377 PMC10830726 38304449

[CR78] Carvalho LJCB, Agustini MAV, Anderson JV, Vieira EA, de Souza CRB, Chen S, Schaal BA, Silva JP (2016) Natural variation in expression of genes associated with carotenoid biosynthesis and accumulation in cassava (*Manihot esculenta* Crantz) storage root. BMC Plant Biol 16:133. 10.1186/s12870-016-0826-0 PMC4902922 27286876

[CR79] Castro-Cegrí A, Zhang L, García-Pérez P, Zengin G, Arikan-Abdulveli B, Balci M, Yildiztugay A, Elbasan F, Gulenturk EO, Yildiztugay E, Lucini L (2025) Tissue-specific metabolic and phenolic profiling of onion (*Allium cepa* L.) in response to nutrient deficiency. Food Chem 493:145738. 10.1016/j.foodchem.2025.145738 40774196

[CR80] Catherwood DJ, Savage GP, Mason SM, Scheffer JJC, Douglas JA (2007) Oxalate content of cormels of Japanese taro (*Colocasia esculenta* (L.) Schott) and the effect of cooking. J Food Compos Anal 20:147–151. 10.1016/j.jfca.2005.12.012

[CR81] Chaicharoen A, Amawan S, Kansup J, Sawwa A (2023) Novel SNP markers and their application in low-cyanide cassava (*Manihot esculenta* crantz) breeding program. Trends Sci 20:6456. 10.48048/tis.2023.6456

[CR82] Chaitra KC, Sarvamangala C, Manikanta DS, Chaitra PA, Fakrudin B (2021) Insights into genetic diversity and population structure of Indian carrot (*Daucus carota* L.) accessions. J Appl Genet 61:303–312. 10.1007/s13353-020-00556-6 32240517

[CR83] Chakraborty N, Ganguly R, Sarkar A, Dasgupta D, Sarkar J, Acharya K, Burachevskaya M, Minkina T, Keswani C (2025) Multifunctional role of brassinosteroids in plant growth, development and defense. J Plant Growth Regul 44:2627–2640. 10.1007/s00344-024-11593-4

[CR84] Chandel R, Singh S, Kumar A, Taak Y, Khar A (2024) Genetic diversity of morphological, biochemical and mineral traits in Indian onion (*Allium cepa*) genotypes. Indian J Agric Sci 94:632–637. 10.56093/ijas.v94i6.140320

[CR85] Chandra A, Rana J, Li Y (2001) Separation, identification, quantification and method validation of anthocyanins in botanica row materials by HPLC and HPLC/MS. J Agric Food Chem 49:3515–3521. 10.1021/jf010389p 11513621

[CR86] Chandrasekara A, Kumar JT (2016) Roots and tuber crops as functional foods: a review on phytochemical constituents and their potential health benefits. Intl J Food Sci 2016:3631647. 10.1155/2016/3631647 PMC4834168 27127779

[CR87] Chang CC, Yang MH, Wen HM, Chern JC (2002) Estimation of total flavonoid content in propolis by two complementary colorimetric methods. J Food Drug Anal 10, 3. 10.38212/2224-6614.2748

[CR88] Chen X, Sun J, Zhu Q, Xiao Y, Zhang H, Huang Y, Wang P, Cao T, Hu R, Xiang Z, Shan N, Zhou Q (2022) Characterizing diversity based on phenotypes and molecular marker analyses of purple yam (*Dioscorea alata*) germplasm in southern China. Genet Resour Crop Evol 69:2501–2513. 10.1007/s10722-022-01388-5

[CR89] Chen M, Lin X, Li X, Gao H, Ming J, Dai M, Zhou Z (2023) Comprehensive Evaluation of Durian Quality Based on Principal Component Analysis and Cluster Analysis. Sci Technol Food Ind 44:278–286 (**(In Chinese with English Abstract)**)

[CR90] Chen L, Chen R, Atwa EM, Mabrouk M, Jiang H, Mou X, Ma X (2024) Nutritional quality assessment of miscellaneous cassava tubers using principal component analysis and cluster analysis. Foods 13:1861. 10.3390/foods13121861 PMC11203269 38928804

[CR91] Cheng KL, Bray RH (1951) Determination of calcium and magnesium in soil and plant material. Soil Sci 72:449–458. 10.1097/00010694-195112000-00005

[CR92] Cheng J, Chen J, Liu X, Li X, Zhang W, Dai Z, Lu L, Zhou X, Cai J, Zhang X, Jiang H, Ma Y (2021) The origin and evolution of the diosgenin biosynthetic pathway in yam. Plant Commun 2:100079. 10.1016/j.xplc.2020.100079 PMC7816074 33511341

[CR93] Cheng H, Jeong E, Cho SW (2025a) Applications of multiplexed CRISPR–Cas for genome engineering. Exp Mol Med 57:1373–1380. 10.1038/s12276-025-01500-6 PMC12322214 40745004

[CR94] Cheng L, Wang N, Bao Z, Zhou Q, Guarracino A, Yang Y, Wang P, Zhang Z, Tang D, Zhang P, Wu Y, Zhou Y, Zheng Y, Hu Y, Lian Q, Ma Z, Lassois L, Zhang C, Lucas WJ, Garrison E, Stein N, Städler T, Zhou Y, Huang S (2025b) Leveraging a phased pangenome for haplotype design of hybrid potato. Nature 640:408–417. 10.1038/s41586-024-08476-9 PMC11981936 39843749

[CR95] Choi Y, Kim S, Lee J (2020) Construction of an onion (*Allium cepa* L.) genetic linkage map using genotyping-by-sequencing analysis with a reference gene set and identification of QTLs controlling anthocyanin synthesis and content. Plants 9:616. 10.3390/plants9050616 PMC7285762 32408580

[CR96] Christensen LP, Kreutzmann S (2007) Determination of polyacetylenes in carrot roots (*Daucus carota* L.) by high-performance liquid chromatography coupled with diode array detection. J Sep Sci 30:483–490. 10.1002/jssc.200600325 17444217

[CR97] Cobbinah FNY, Kyei SK, Azanu D, Kabange RS (2025) Bioaccumulation factor and health hazards of heavy metals in taro in the Atiwa East Municipality, Ghana. Soil Sediment Contam 1–21. 10.1080/15320383.2025.2595647

[CR98] Coe K, Bostan H, Rolling W, Turner-Hissong S, Macko-Podgórni A, Senalik D, Liu S, Seth R, Curaba J, Mengist MF, Grzebelus D, Deynze AV, Dawson J, Ellison S, Simon P, Lorizzo M (2023) Population genomics identifies genetic signatures of carrot domestication and improvement and uncovers the origin of high-carotenoid orange carrots. Nat Plants 9:1643–1658. 10.1038/s41477-023-01526-6 PMC10581907 37770615

[CR99] Colla G, Kim H-J, Kyriacou MC, Rouphael Y (2018) Nitrate in fruits and vegetables. Sci Hortic 237:221–238. 10.1016/j.scienta.2018.04.016

[CR100] Conversa G, Bonasia A, Lazzizera C, Elia A (2016) Bio-physical, physiological, and nutritional aspects of ready-to-use Cima di rapa (*Brassica rapa* L. subsp. *sylvestris* L. Janch. var. *esculenta* Hort.) as affected by conventional and organic growing systems and storage time. Sci Hortic 213:76–86

[CR101] Conversa G, Lazzizera C, Bonasia A, La Rotanda P, Elia A (2020) Nutritional characterization of two rare landraces of turnip (*Brassica rapa*. var. *rapa*) tops and their on-farm conservation in Foggia province. Sustainability 12:3842. 10.3390/su12093842

[CR102] Crupi P, Faienza MF, Naeem MY, Corbo F, Clodoveo ML, Muraglia M (2023) Overview of the potential beneficial effects of carotenoids on consumer health and well-being. Antioxidants (Basel) 12:1069. 10.3390/antiox12051069 PMC10215867 37237935

[CR103] Cui P, Li Y, Cui C, Huo Y, Lu G, Yang H (2020) Proteomic and metabolic profile analysis of low-temperature storage responses in *Ipomoea batata* Lam. tuberous roots. BMC Plant Biol 20:435. 10.1186/s12870-020-02642-7 PMC7507648 32957906

[CR104] Cunnif P (2000) Official methods of association of official analytical chemists. J Assoc off Anal Chem 2:1–37

[CR105] Curaba J, Bostan H, Cavagnaro PF, Senalik D, Mengist MF, Zhao Y, Simon PW, Lorizzo M (2020) Identification of an SCPL gene controlling anthocyanin acylation in carrot (*Daucus carota* L.) root. Front. Plant Sci. 10:1770. 10.3389/fpls.2019.01770 PMC7005140 32082341

[CR107] Darkwa K, Agre P, Olasanmi B, Iseki K, Matsumoto R, Powell A, Bauchet G, De Koeyer D, Murnaka S, Adebola P, Asiedu R, Terauchi R, Asfaw A (2020) Comparative assessment of genetic diversity matrices and clustering methods in white Guinea yam (Dioscorea rotundata) based on morphological and molecular markers. Sci Rep 10:13191. 10.1038/s41598-020-69925-9 PMC7413250 32764649

[CR108] de Brauw A, Eozenou P, Gilligan DO, Hotz C, Kumar N, Meenakshi JV (2018) Biofortification, crop adoption and health information: impact pathways in Mozambique and Uganda. Am J Agric Econ 100:906–930. 10.1093/ajae/aay005 PMC7053385 32139914

[CR109] de Carvalho RRB, Marmolejo Cortes DF, Bandeira e Sousa M, de Oliveira LA, de Oliveira EJ (2022) Image-based phenotyping of cassava roots for diversity studies and carotenoids prediction. PLoS ONE 17:e0263326. 10.1371/journal.pone.0263326 PMC8803208 35100324

[CR110] De Temmerman L, Waefeneers N, Thiry C, Laing GD, Tack F, Ruttens A (2014) Selenium content of Belgian cultivated soils and its uptake by field crops and vegetables. Sci Total Environ 468–469:77–82. 10.1016/j.scitotenv.2013.08.016 24013513

[CR111] De Vos RCH, Moco S, Lommen A, Keurentjes JJB, Bino RJ, Hall RD (2007) Untargeted large-scale plant metabolomics using liquid chromatography coupled to mass spectrometry. Nat Protoc 2:778–791. 10.1038/nprot.2007.95 17446877

[CR113] Deng YJ, Duan AQ, Liu H, Wang YH, Zhang RR, Xu ZS, Xiong AS (2023) Generating colorful carrot germplasm through metabolic engineering of betalains pigments. Hortic Res. 10.1093/hr/uhad024 PMC10541523 37786858

[CR112] Deng Y-J, Duan A-Q, Li T, Tan S-S, Liu S-S, Wang Y-H, Ma J, Li J-W, Liu H, Xu Z-S, Liang Y, Zhou J-H, Xiong A-S (2025) Altering carotene hydroxylase activity of DcCYP97C1 affects carotenoid flux and changes taproot colour in carrot. Plant Cell Environ 48:3118–3135. 10.1111/pce.15331 39692072

[CR114] Dhaliwal SS, Sharma V, Shukla AK, Verma V, Kaur M, Shivay YS, Nisar S, Gaber A, Brestic M, Barek V, Skalickly M, Ondrisik P, Hossain A (2022) Biofortification—a frontier novel approach to enrich micronutrients in field crops to encounter the nutritional security. Molecules. 10.3390/molecules27041340 PMC8877941 35209127

[CR115] Dhiman AS, Taliadoros D, Stukenbrock EH, McGrath JM, Emrani N, Jung C (2025) Signatures in domesticated beet genomes pointing at genes under selection in a sucrose-storing root crop. BMC Biol 23:299. 10.1186/s12915-025-02422-5 PMC12505646 41057828

[CR116] Ding Z, Fu L, Wang B, Ye J, Ou W, Yan Y, Li M, Zeng L, Dong X, Tie W, Ye X, Yang J, Xie Z, Wang Y, Guo J, Chen S, Xiao X, Wan Z, An F, Zhang J, Peng M, Luo J, Li K, Hu W (2023) Metabolic GWAS-based dissection of genetic basis underlying nutrient quality variation and domestication of cassava storage roots. Genome Biol 24:289. 10.1186/s13059-023-03137-y PMC10722858 38098107

[CR117] Divya K, Thangaraj M, Krishna Radhika N (2024) CRISPR/Cas9: an advanced platform for root and tuber crops improvement. Front Genome Ed 5:1242510. 10.3389/fgeed.2023.1242510 PMC10836405 38312197

[CR118] Dong W, He F, Jiang H, Liu L, Qiu Z (2021) Comparative transcriptome sequencing of taro corm development with a focus on the starch and sucrose metabolism pathway. Front Genet 12:771081. 10.3389/fgene.2021.771081 PMC8630585 34858484

[CR119] Dossa K, Morel A, Houngbo ME, Mota AZ, Malédon E, Irep J-L, Diman J-L, Mournet P, Causse S, Van Nguyen K, Cornet D, Chair H (2022) Genome-wide association studies reveal novel loci controlling tuber flesh color and oxidative browning in Dioscorea alata. J Sci Food Agric 104:4895–4906. 10.1002/jsfa.12721 37209230

[CR120] Du H, Zhai Z, Pu J, Liang J, Wang R, Zhang Z, Wang P, Zhu Y, Huang L, Li D, Chen K, Zhu G, Zhang C (2025) Two tandem R2R3 MYB transcription factor genes cooperatively regulate anthocyanin accumulation in potato tuber flesh. Plant Biotechnol J 2:1521–1534. 10.1111/pbi.14602 PMC12018810 39887502

[CR121] Duan AQ, Deng Y-J, Tan S-S, Liu S-S, Liu H, Xu Z-S, Shu S, Xiong A-S (2024) *DcGST1*, encoding a glutathione S-transferase activated by *DcMYB7*, is the main contributor to anthocyanin pigmentation in purple carrot. Plant J 117:1069–1083. 10.1111/tpj.16539 37947285

[CR122] Duarte-Delgado D, Juyó D, Gebhardt C, Sarmiento F, Mosquera-Vásquez T (2017) Novel SNP markers in *InvGE* and *SssI* genes are associated with natural variation of sugar contents and frying color in *Solanum tuberosum* Group Phureja. BMC Genet 18:23. 10.1186/s12863-017-0489-3 PMC5345157 28279167

[CR123] Ducreux LJM, Morris WL, Hedley PE, Shepherd T, Davies HV, Millam S, Taylor MA (2005) Metabolic engineering of high carotenoid potato tubers containing enhanced levels of β-carotene and lutein. J Exp Bot 56:81–89. 10.1093/jxb/eri016 15533882

[CR124] Dumas ME, Gauguier D (2012) Mapping metabolomic quantitative trait loci (mQTL): a link between metabolome-wide association studies and systems biology. In Suhre K (eds) Genetics Meets Metabolomics. Springer, New York. 10.1007/978-1-4614-1689-0_14

[CR125] Dunemann F, He W, Böttcher C, Reichardt S, Nothnagel T, Heuvelmans P, Hermans F (2022) The genetic control of polyacetylenes involved in bitterness of carrots (*Daucus carota* L.): Identification of QTLs and candidate genes from the plant fatty acid metabolism. BMC Plant Biol 22:92. 10.1186/s12870-022-03484-1 PMC8889737 35232393

[CR126] Durante M, Lenucci MS, Marrese PP, Rizzi V, De Caroli M, Piro G, Fini P, Russo GL, Mita G (2016) α-Cyclodextrin encapsulation of supercritical CO_2_ extracted oleoresins from different plant matrices: a stability study. Food Chem 199:684–693. 10.1016/j.foodchem.2015.12.073 26776025

[CR128] Dwivedi SL, Ortiz R (2021) Food crops low in seed-phytate improve human and other non-ruminant animals’ health. Crop Breed Genet Genom 3:e210009. 10.20900/cbgg20210009

[CR127] Dwivedi SL, Garcia-Oliveira AL, Govindaraj M, Ortiz R (2023) Biofortification to avoid malnutrition in humans in a changing climate: enhancing micronutrient bioavailability in seed, tuber, and storage roots. Front Plant Sci 14:1119148. 10.3389/fpls.2023.1119148 PMC9923027 36794214

[CR129] Ebell LF (1969) Variation in total soluble sugars of conifer tissues with method of analysis. Phytochemistry 8:227–233. 10.1016/S0031-9422(00)85818-5

[CR130] Ebert AW (2020) The role of vegetable genetic resources in nutrition security and vegetable breeding. Plants 9:736. 10.3390/plants9060736 PMC7357112 32545299

[CR131] Egan SV, Yeoh HH, Bradbury JH (1999) Simple picrate paper kit for determination of the cyanogenic potential of cassava flour. J Sci Food Agric 76:39–48. 10.1002/(SICI)1097-0010(199801)76:1<39::AID-JSFA947>3.0.CO;2-M

[CR132] Ehounou AE, Cormier F, Maledon E, Nudol E, Vignes H, Gravillon MC, N’guetta ASP, Mournet P, Chaïr H, Kouakou AM, Arnau G (2022) Identification and validation of QTLs for tuber quality related traits in greater yam (*Dioscorea alata* L.). Sci Rep 12:8423. 10.1038/s41598-022-12135-2 PMC9120205 35589821

[CR133] Ekaette E, Nwofia E, Okocha P, Nnnabue I, Eluwa K, Obidiegwu J, Agre PA (2024) Exploring the genetic diversity and population structure of aerial yams (*Dioscorea bulbifera* L.) DArT-seq and agronomic traits. PLoS ONE 19:e0306631. 10.1371/journal.pone.0306631 PMC11343425 39178185

[CR134] El Hanafi S, Jiang Y, Kehel Z, Schulthess AW, Zhao Y, Mascher M, Haupt M, Himmelbach A, Stein N, Amri A, Reif JC (2023) Genomic predictions to leverage phenotypic data across genebanks. Front Plant Sci 14:1227656. 10.3389/fpls.2023.1227656 PMC10493331 37701801

[CR136] Ellison S, Senalik D, Bostan H, Lorizzo M, Simon P (2017) Fine mapping, transcriptome analysis, and marker development for *Y*_*2*_, the gene that conditions β-carotene accumulation in carrot (*Daucus carota* L.). G3 (Bethesda) 7:2665–2675. 10.1534/g3.117.043067 PMC5555471 28663343

[CR135] Ellison SL, Luby CH, Corak KE, Coe KM, Senalik D, Iorizzo M, Goldman IL, Simon PW, Dawson JC (2018) Carotenoid presence is associated with the *Or* gene in domesticated carrot. Genetics 210:1497–1508. 10.1534/genetics.118.301299 PMC6283172 30352832

[CR137] Englyst HN, Cummings JH (1988) Improved method for measurement of dietary fiber as non-starch polysaccharides in plant foods. J Assoc off Anal Chem. 10.1093/jaoac/71.4.808 2458334

[CR138] Esuma W, Ozimati A, Kulakow P, Gore MA, Wolfe MD, Nuwamanya E, Egesi C, Kawuki RS (2021) Effectiveness of genomic selection for improving provitamin A carotenoid content and associated traits in cassava. G3 (Bethesda) 11:jkab160. 10.1093/g3journal/jkab160 PMC8496257 33963852

[CR139] Esuma W, Eyoo O, Gwandu F, Mukasa S, Alicai T, Ozimati A, Nuwamanya E, Rabbi I, Kawuk R (2022) Validation of KASP markers associated with cassava mosaic disease resistance, storage root dry matter and provitamin A carotenoid contents in Ugandan cassava germplasm. Front Plant Sci 13:1017275. 10.3389/fpls.2022.1017275 PMC9727383 36507387

[CR140] Etumnu CE (2016) Behaviral determinants of biofortified food acceptance: the case of the orange-fleshed sweet potato in Ghana. Master’s Thesis, Univ of San Francisco. https://repository.usfca.edu/thes/173

[CR141] Eyinla TE, Maziya-Dixon B, Alamu OE, Sanusi RA (2019) Retention of provitamin A content in products from new biofortified cassava varieties. Foods 8:177. 10.3390/foods8050177 PMC6560409 31137653

[CR142] Fan L, Wang Y, Xu L, Tang M, Zhang X, Ying J, Li C, Dong J, Liu L (2020) A genome-wide association study uncovers a critical role of the *RsPAP2* gene in red-skinned *Raphanus sativus* L. Hortic Res 7:164. 10.1038/s41438-020-00385-y PMC7518265 33042558

[CR143] Fan Y, Xue L, Shang M, Gao S, Zhao N, Zhai H, He S, Zhang H, Liu Q (2025) Natural allelic variation of *NAC transcription factor 22* regulates starch biosynthesis and properties in sweet potato. J Integr Plant Biol 67:1879–1894. 10.1111/jipb.13916 40317899

[CR144] FAO (1990) Roots, tubers, plantain and bananas in human nutrition. Food and Agriculture Organization of the United Nations, Rome, Italy

[CR146] FAO (2023) Third report on the state of the world’s plant genetic resources for food and agriculture. Commission on Genetic Resources for Food and Agriculture, Nineteenth Regular Session. Food and Agriculture Organization of the United Nations, Rome, Italy

[CR145] FAO. (1998). Carbohydrates in human nutrition. Report of a Joint FAO/WHO Expert Consultation. FAO Food and Nutrition Paper No. 66 [Access: 31^st^ August, 2026] 9743703

[CR350] Sayre RT, Beeching, JR, Cahoon, EB, Egesi, C, Fauquet, C, Fellman J, Fregene M, Gruissem W, Mallowa S, Manary M, Maziya-Dixon B, Mbanaso A, Schachtman DP, Siritunga D, Taylor N, Vanderschren H, Zhang P (2011) The BioCassava Plus program: biofortification of Cassava for Sub-Saharan Africa. Annu Rev Plant Biol 62:251–272. doi.org. 10.1146/annurev-arplant-042110-103751 21526968

[CR147] Fernando EAJ, Selvaraj MG, Delgado A, Rabbi I, Kulakow P (2022) Frontline remote sensing tool to locate hidden traits in root and tuber crops. Mol Plant 15:1500–1502. 10.1016/j.molp.2022.08.010 36045578

[CR148] Figueira JG, Barros SC, Valero-Cases E, Silva AS (2026) Glycoalkaloids in potatoes: exploring health effects, analytical techniques, occurrence, mitigation measures. Food Chem Toxicol 207:115807. 10.1016/j.fct.2025.115807 41106768

[CR150] Freidig AK, Goldman IL (2011) Variation in oxalic acid content among commercial table beet cultivars and related crops. J Am Soc Hortic Sci 136:54–60. 10.21273/JASHS.136.1.54

[CR151] Friedmann M, Asfaw A, Anglin NL, Becerra LA, Bhattacharjee R, Brown A, Carey E, Ferguson ME, Gemenet D, Lindqvist-Kreuze H, Rabbi I, Rouard M, Swennen R, Thiele G (2018) Genomics-assisted breeding in the CGIAR research program on roots, tubers and bananas (RTB). Agriculture 8:89. 10.3390/agriculture8070089

[CR152] Fu L, Ding Z, Tie W, Yang J, Yan Y, Hu W (2022) Integrated metabolomic and transcriptomic analyses reveal novel insights of anthocyanin biosynthesis on color formation in cassava tuberous roots. Front Nutr 9:842693. 10.3389/fnut.2022.842693 PMC9017287 35449540

[CR153] Fufa TW, Abtew WG, Amadi CO, Oselebe HO (2022) DArTSeq SNP-based genetic diversity and population structure studies among taro [(*Colocasia esculenta* (L.) Schott] accessions sourced from Nigeria and Vanuatu. PLoS One 17:e0269302. 10.1371/journal.pone.0269302 PMC9648780 36355796

[CR154] Fumia N, Nair R, Lin Y-P, Lee C-R, Chen H-W, von Wettberg EB, Kantar M, Schafleitner R (2023) Leveraging genomics and phenomics to accelerate improvement in mungbean: A case study in how to go from GWAS to selection. Plant Phenome J 6:e20088. 10.1002/ppj2.20088

[CR155] Gallik S (2012) Determination of the anthocyanin concentration in table wines and fruit juices using visible light spectrophotometry. Cell Biol 2:1–12

[CR156] Galvao AC, Nicoletto C, Zanin G, Vargas PF, Sambo P (2021) Nutraceutical content and daily value contribution of sweet potato accessions for the European market. Horticulturae 7:23. 10.3390/horticulturae7020023

[CR157] Gannon BM, Thakker V, Bonam VS, Haas JD, Bonam W, Finkelstein JL, Udipi SA, Mehta S (2019) A randomized crossover study to evaluate recipe acceptability in breastfeeding mothers and young children in India targeted for multiple biofortified food crop intervention. Food Nutr Bull 40:460–470. 10.1177/0379572119855588 PMC7473068 31359782

[CR158] Gatarira C, Agre P, Matsumoto R, Edemodu A, Adetimirin V, Bhattacharjee R, Asiedu R, Asfaw R (2020) Genome-wide association analysis for tuber dry matter and oxidative browning in water yam (*Dioscorea alata* L.). Plants (Basel) 9:969. 10.3390/plants9080969 PMC7464735 32751829

[CR159] GB 5009.14–2017 “Determination of Zn in Foods.”

[CR160] GB 5009.241–2017 “Determination of Mg in Foods.”

[CR161] GB 5009.242–2017 “Determination of Mn in Foods.”

[CR163] GB 5009.9–2016; National Standard for Food Safety, the Determination of Starch in Food. The National Health and Family Planning Commission of the People’s Republic of China: Beijing, China; The China Food and Drug Administration, and the Standardization Administration of the People’s Republic of China. China Standards Press: Beijing, China, 2016.

[CR162] GB 5009.90–2016 “Determination of Iron in Foods.”

[CR164] GB 5009.92–2016 “Determination of Ca in Foods.”

[CR303] GB5009.88–2014. National Standard for Food Safety, the Determination of Dietary Fiber in Food; The National Health and Family Planning Commission of the People’s Republic of China. Standardization Administration of the People’s Republic of China: Beijing, China; China Standards Press: Beijing, China, 2014.

[CR165] Gemenet DC, da Silva PG, Boeck BD, Wood JC, Mollinari M, Olukolu BA, Diaz F, Mosquera V, Ssali RT, David M, Kitavi MN, Burgos G, Felde TZ, Ghislain M, Carey E, Swanckaert J, Coin LJM, Fei Z, Hamilton JP, Yada B, Yencho GC, Zeng Z-B, Mwanga ROM, Khan A, Grunberg WJ, Buell CR (2020) Quantitative trait loci and differential gene expression analyses reveal the genetic basis for negatively associated β-carotene and starch content in hexaploid sweet potato [*Ipomoea batatas* (L.) Lam.]. Theor Appl Genet 133:23–36. 10.1007/s00122-019-03437-7 PMC6952332 31595335

[CR166] Gerardi C, Albano C, Calabriso N, Carluccio MA, Durante M, Mita G, Renna M, Serio F, Blando F (2018) Techno-functional properties of tomato puree fortified with anthocyanin pigments. Food Chem 240:1184–1192. 10.1016/j.foodchem.2017.08.057 28946241

[CR167] Girma G, Bhattacharjee R, Lopez-Montes A, Gueye B, Ofodile S, Franco J, Abberton M (2017) Re-defining the yam (*Dioscorea* spp.) core collection using morphological traits. Plant Genet Resour 16:193–200. 10.1017/S1479262117000144

[CR168] Giusti MM, Wrolstad RE (2001) Characterization and measurement of anthocyanins by UV-visible spectroscopy. Curr Protoc Food Anal Chem 2001, F1.2.1–F1.2.13. 10.1002/0471142913.faf0102s00

[CR483] Koch, T.C., Goldman, I.L. (2005). Relationship of carotenoids and tocopherols in a sample of carrot root-colour accessions and carrot germplasm carrying Rp and rp alleles. J. Agric. Food Chem. 53, 325–331 10.1021/jf048272z 15656668

[CR169] Gomez MA, Berkoff KC, Gill BK, Iavarone AT, Lieberman SE, Ma JM, Schultink A, Karavolias NG, Wyman SK, Chauhan RD, Taylor NJ, Staskawicz BJ, Cho MJ, Rokhsar DS, Lyons JB (2023) CRISPR-Cas9-mediated knockout of *CYP79D1* and *CYP79D2*in cassava attenuates toxic cyanogen production. Front Plant Sci 13:1079254. 10.3389/fpls.2022.1079254 PMC10064795 37007603

[CR170] Gong L, Zhang L, Zhang H, Nie F, Liu Z, Liu X, Fang M, Yang W, Zhang Y, Zhang G, Guo Z, Zhang H (2025) Haplotype-resolved genome assembly and genome-wide association study identifies the candidate gene closely related to sugar content and tuber yield in *Solanum tuberosum*. Hortic Res 12: uhaf075. 10.1093/hr/uhaf075 PMC12038253 40303439

[CR171] González MN, Massa GA, Andersson M, Turesson H, Olsson N, Fält A-S, Storani L, Décima Oneto CA, Hofvander P, Feingold SE (2020) Reduced enzymatic browning in potato tubers by specific editing of a polyphenol oxidase gene via ribonucleoprotein complexes delivery of the CRISPR/Cas9 system. Front Plant Sci 10:1649. 10.3389/fpls.2019.01649 PMC6962139 31998338

[CR172] Gowda KH, Chauhan VBS, Bhagwat A, Pati K, Nedunchezhiyan M, Hegde V (2025) Plant genetic resources in cassava: collection, characterization, evaluation, conservation, and utilization. In: Kalia P (ed) Vegetable crops. Springer, Singapore, pp 1431–1459. 10.1007/978-981-97-8949-8

[CR173] Gras CC, Bogner H, Carle R, Schweiggert RM (2016) Effect of genuine non-anthocyanin phenolics and chlorogenic acid on color and stability of black carrot (*Daucus carota* ssp. *sativus* var. *atrorubens* Alef.) anthocyanins. Food Res Int 85:291–300. 10.1016/j.foodres.2016.05.006 29544847

[CR174] Guimarães JB, Simões-Costa MC, Pinho M, Godinho CM, SáPereira P, Neves Martins J, Ribeiro-Barros A, Talhinhas P, Veloso MM (2025) Genetic diversity of sweet potato (*Ipomoea batatas* (L.) Lam.) from Portugal, Mozambique and Timor-Leste. Biology (Basel) 14:1602. 10.3390/biology14111602 PMC12650677 41300390

[CR175] Gupta M, Gupta S (2017) An overview of selenium uptake, metabolism, and toxicity in plants. Front Plant Sci 7(1):2074 10.3389/fpls.2016.02074 PMC5225104 28123395

[CR176] Gupta AJ, Gorrepati K, Bibwe B, Kaldate S, Volaguthala S, Mahajan V (2025) Studies on biochemical and nutraceutical profiling of 43 Indian onion (*Allium cepa* L.) genotypes. Food Measure 19:6614–6624. 10.1007/s11694-025-03425-0

[CR177] Hadagali S, Stelmach-Wityk K, Macko-Podgórni A, Grzebelus D (2024) Polymorphic insertions of DcSto miniature inverted-repeat transposable elements reveal genetic diversity structure within the cultivated carrot. J Appl Genet 66:293–303. 10.1007/s13353-024-00916-6 PMC12000206 39466592

[CR178] Hakim MS, Ikram A, Salem-Bekhit MM, Baloch Z (2025) Unravelling circRNAs and miRNAs: key regulators in immune-related diseases. Front Immunol 16:1739926. 10.3389/fimmu.2025.1739926 PMC12698395 41394812

[CR484] Haque MR, Bradbury JH (2002) Total cyanide determination of plants and foods using the picrate and acid hydrolysis methods. Food Chem 77, 107–114. 10.1016/S0308-8146(01)00313-2

[CR179] Hara-Skrzypiec A, Śliwka J, Jakuczun H, Zimnoch-Guzowska (2018) QTL for tuber morphology traits in diploid potato. J Appl Genet 59:123–132. 10.1007/s13353-018-0433-x PMC5895667 29492845

[CR180] Harborne JB, Williams CA (1973) A chemotaxonomic survey of flavonoids and simple phenols in leaves of the Ericaceae. Bot J Linn Soc 66:37–54. 10.1111/j.1095-8339.1973.tb02159.x

[CR181] Hardigan MA, Laimbeer FPE, Newton L, Crisovan E, Hamilton JP, Vaillancourt B, Wiegert-Rininger K, Wood JC, Douches DS, Farré EM, Veilleux RE, Buell CR (2017) Genome diversity of tuber-bearing *Solanum* uncovers complex evolutionary history and targets of domestication in the cultivated potato. Proc Natl Acad Sci U S A 114:E9999–E10008. 10.1073/pnas.1714380114 PMC5699086 29087343

[CR182] Harris KP, Goddard JR, Cullen A, Reynolds T, Anderson CL (2015) Root, tuber, and banana textural traits: a review of the available food science and consumer preferences literature. Evans School Policy Analysis and Research (EPAR) Brief No. 295, Univ of Wash. https://epar.evans.uw.edu/root-tuber-and-banana-textural-traits-a-review-of-the-available-food-science-and-consumer-preferences-literature/

[CR183] HarvestPlus (2024). Retrieved at https://www.harvestplus.org/home/global-reach/

[CR184] Havey MJ (2024) Genetic analysis of the shape and volume of onion bulbs and daylength effect on bulbing. J Amer Soc Hortic Sci 149:86–91. 10.21273/JASHS05362-23

[CR185] Havlin JL, Soltanpour PN (1980) A nitric acid plant tissue digest method for use with inductively coupled plasma spectrometry. Commun Soil Sci Plant Anal 11:969–980. 10.1080/00103628009367096

[CR186] Hawley RS, Gilliland WD (2006) Sometimes the result is not the answer: the truths and the lies that come from using the complementation test. Genetics 174:5–15. 10.1534/genetics.106.064550 PMC1569807 16988106

[CR187] He W, Dunemann F (2025). Endogenous Falcarinol-Type Polyacetylenes in Carrots and Their Putative Influence on Post-Harvest Fungal Pathogens Mycocentrospora Acerina and Botrytis Cinerea. 10.1101/2025.01.08.631919

[CR188] He L, Liu X, Liu S, Zhang J, Zhang Y, Sun Y, Tang R, Wang W, Cui H, Li R, Zhu Z, Jia X (2020) Transcriptomic and targeted metabolomic analysis identifies genes and metabolites involved in anthocyanin accumulation in tuberous roots of sweetpotato (*Ipomoea batatas* L.). Plant Physiol Biochem 156:323–332. 10.1016/j.plaphy.2020.09.021 32998099

[CR189] He S, Wang H, Hao X, Wu Y, Bian X, Yin M, Zhang Y, Fan W, Dai H, Yuan L, Zhang P, Chen L (2021) Dynamic network biomarker analysis discovers IbNAC083 in the initiation and regulation of sweet potato root tuberization. Plant J 108:793–813. 10.1111/tpj.15478 34460981

[CR190] He H-J, Wang Y, Zhang M, Wang Y, Ou X, Guo J (2022) Rapid determination of reducing sugar content in sweet potatoes using NIR spectra. J Food Compos Anal 111:104641. 10.1016/j.jfca.2022.104641

[CR191] He M, Ma X, Zhou Y, Wang F, Fang G, Wang J (2024) Combined metabolome and transcriptome analyses reveals anthocyanin biosynthesis profiles between purple and white potatoes. Int J Mol Sci 25:12884. 10.3390/ijms252312884 PMC11641821 39684596

[CR192] He C, Mkunga JJ, Zhang D, Choudhary MI, Wang Y, Liu Y, Fei L, Shan C, Yang X, Cai W (2025) Metabolite-based color codes and antioxidant mechanisms in carrots: insights from untargeted metabolomics and bioinformatics. Food Chem 496:146637 10.1016/j.foodchem.2025.146637 41075649

[CR193] Heping H, Shanlin G, Lanlan C, Xiaoke J (2008) In vitro induction and identification of autotetraploids of *Dioscorea zingiberensis*. In Vitro Cell Dev Biol - Plant 44, 448–55. https:// doi. org/ 10. 1007/ s11627- 008- 9177–3

[CR194] Hernandez CO, Vogel G (2026) Toward an art of genomic selection in vegetable breeding. Crop Sci 66:e70225. 10.1002/csc2.70225

[CR195] Hesse PR (1971) A textbook of soil chemical analysis, 2nd ed. John Muray Company Ltd., London

[CR196] Hochmuth A, Carswell M, Rowland A, Scarbrough D, Esch L, Kamble NU, Habig JW, Seung D (2025) Distinct effects of *PTST2b* and *MRC* on starch granule morphogenesis in potato tubers. Plant Biotechnol J 23:412–429. 10.1111/pbi.14505 PMC11772324 39659019

[CR197] Hosotani K, Kitagawa M (2003) Improved simultaneous determination method of β-carotene and retinol with saponification in human serum and rat liver. J Chromatogr B 791:305–313. 10.1016/S1570-0232(03)00233-2 12798190

[CR198] Hou D, Jia X, Wang L, McGrath SP, Zhu Y-G, Hu Q, Zhao F-J, Bank MS, O’Connor D, Nriagu J (2025) Global soil pollution by toxic metals threatens agriculture and human health. Sci 338:6744. 10.1126/science.adr5214 40245139

[CR199] Hu J, Hu J, Duan S, Xiang C, Duan Y, Zhang S, Li G (2025) Transcriptome analysis reveals co-expression regulation of sugar transport and signaling networks in initiating stolon-to-tuber transition in potato. Int J Mol Sci 26:5278. 10.3390/ijms26115278 PMC12154728 40508086

[CR200] Huang C, Hang Y, Zhou Y, Guo K (2003) Analysis on quality of some main populations of Dioscorea zingiberensis in China. Chem Indus for Prod 23:68–72

[CR201] Huang B, Xin J, Dai H, Zhou W, Peng L (2015) Identification of low-Cd cultivars of sweet potato (*Ipomoea batatas* (L.) Lam.) after growing on Cd-contaminated soil: uptake and partitioning to the edible roots. Environ Sci Pollut Res 22:11813–11821. 10.1007/s11356-015-4449-z 25860549

[CR202] Huey SL, Bhargava A, Friesen VM, Konieczynski EM, Krisher JT, Mbuya MNN, Mehta NH, Monterrosa E, Nyangaresi AM, Mehta S (2023) Sensory acceptability of biofortified foods and food products: a systematic review. Nutr Rev 82:892–912. 10.1093/nutrit/nuad100 PMC11163453 37634146

[CR203] Huh SM, Cho A, Yim B, Kim J, Jeong JG, Jang H, Mun J-H, Yu H-J (2023) Characterization of agronomic traits and genomic diversity in a newly assembled radish core collection. Crop Sci 64:88–109. 10.1002/csc2.21135

[CR205] International Potato Center (CIP) (2019) Minicore collection of potato landraces. https://cipotato.org/genebankcip/genebank_media/minicore-collection-of-potato-landraces/#/

[CR204] Ikeogu UN, Akdemir D, Wolfe MD, Okeke UG, Chinedozi A, Jannink J-L, Egesi CN (2019) Genetic correlation, genome-wide association and genomic prediction of portable NIRS-predicted carotenoids in cassava roots. Front Plant Sci 10:1570. 10.3389/fpls.2019.01570 PMC6904298 31867030

[CR206] Ioniță-Mîndrican C-B, Ziani K, Mititelu M, Oprea E, Neacșu SM, Moroșan E, Dumitrescu DE, Roșca AC, Drăgănescu D, Negrei C (2022) Therapeutic benefits and dietary restrictions of fiber intake: a state of the art review. Nutrients 14:2641. 10.3390/nu14132641 PMC9268622 35807822

[CR207] Jagadish LK, Krishnan VV, Shenbhagaraman R, Kaviyarasan V (2009) Comparative study on the antioxidant, anticancer and antimicrobial property of *Agaricus bisporus* (JE Lange) Imbach before and after boiling. Afr J Biotechnol 8:654–661

[CR208] Jammer A, Albacete A, Schulz B, Koch W, Weltmeier F, van der Graaff E, Pfeifhofer HW, Roitsch TG (2020) Early-stage sugar beet taproot development is characterized by three distinct physiological phases. Plant Direct 4:e00221. 10.1002/pld3.221 PMC7395582 32766510

[CR209] Jarret R, Anglin N, Ellis D, Villordon A, Wadl P, Jackson M (2019) Sweetpotato genetic resources: today and tomorrow. Burleigh Dodds, Cambridge, UK, pp 1–52

[CR210] Jelodarian S, Ebrahimabadi AH, Khalighi A, Batooli H (2012) Evaluation of antioxidant activity of *Malus domestica* fruit extract from Kashan area. Avicenna J Phytomed 2:139 PMC4075675 25050243

[CR211] Jia R, Zhang R, Gangurde SS, Tang C, Jiang B, Li G, Wang Z (2022) Integrated analysis of carotenoid metabolites and transcriptome identifies key genes controlling carotenoid compositions and content in sweetpotato tuberous roots (*Ipomoea batatas* L.). Front Plant Sci 13:993682. 10.3389/fpls.2022.993682 PMC9632283 36340393

[CR212] Jia H, Zhao Q, Song J, Zhang X, Yang W, Du Z, Zhu Y, Wang H (2023) Large-scale population structure and genetic architecture of agronomic traits of garlic. Hortic Res 10: uhad034. 10.1093/hr/uhad034 PMC10548411 37799626

[CR213] Jim´enez-Amezcua I, Sanz ML, Díez-Municio M, Parker K, Oruna-Concha MJ, Soria AC (2025) Changes in the bioactive content and aroma profile of aged garlic with processing conditions. LWT - Food Sci Technol 118003. 10.1016/j.lwt.2025.118003

[CR214] Jo KR, Cho S, Cho J-H, Park H-J, Choi J-G, Park Y-E, Cho K-S (2022) Analysis of genetic diversity and population structure among cultivated potato clones from Korea and global breeding programs. Sci Rep 12:10462. 10.1038/s41598-022-12874-2 PMC9213424 35729234

[CR215] Julius BT, Leach KA, Tran TM, Mertz RA, Braun DM (2017) Sugar transporters in plants: new insights and discoveries. Plant Cell Physiol 58:1442–1460. 10.1093/pcp/pcx090 28922744

[CR216] Juma BS, Mukami A, Mweu C, Ngugi MP, Mbinda W (2022) Targeted mutagenesis of the *CYP79D1* gene via CRISPR/Cas9-mediated genome editing results in lower levels of cyanide in cassava. Front Plant Sci 13:1009860. 10.3389/fpls.2022.1009860 PMC9644188 36388608

[CR217] Kalia P (ed) (2025) Vegetable crops. Springer, Singapore. 10.1007/978-981-97-8949-8

[CR219] Kanaabi M, Namakula FB, Nuwamanya E, Kayondo IS, Muhumuza N, Wembabazi E, Iragaba P, Nandudu L, Nanyonjo AR, Baguma J, Esuma W, Ozimati A, Settumba M, Alicai T, Ibanda A, Kawuki RS (2024b) Rapid analysis of hydrogen cyanide in fresh cassava roots using NIRS and machine learning algorithms: meeting end user demand for low cyanogenic cassava. Plant Genome 17:e20403. 10.1002/tpg2.20403 PMC12806942 37938872

[CR220] Kanaabi M, Settumba MB, Nuwamanya E, Muhumuza N, Iragaba P, Ozimati A, Namakula FB, Kayondo IS, Baguma JK, Nanyonjo AR, Esuma W, Kawuki RS (2024c) Genetic variation and heritability for hydrogen cyanide in fresh cassava roots: implications for low-cyanide cassava breeding. Plants 13:1186. 10.3390/plants13091186 PMC11085877 38732401

[CR218] Kanaabi M, Mukasa SB, Nuwamanya E, Iragaba P, Baguma JK, Nanyonjo AR, Wagaba H, Muhumuza N, Namakula FB, Wembabazi E, Ozimati A, Kayondo IS, Esuma W, Kawuki RS (2024a) Validation of KASP markers associated with hydrogen cyanide in fresh cassava roots in Uganda cassava germplasm. Agronomy 14:2765. 10.3390/agronomy14122765

[CR221] Kant S (2018) Understanding nitrate uptake, signaling and remobilization for improving plant nitrogen use efficiency. Semin Cell Dev Biol 74:89–96. 10.1016/j.semcdb.2017.08.034 28838687

[CR222] Karan YB (2023) Mineral nutrient variability of potato (*Solanum tuberosum* L.) tubers with different colors grown in Niksar, Kazova and Artova locations of Tokat Province, Turkey. PeerJ 11:e15262. 10.7717/peerj.15262 PMC10259439 37312874

[CR223] Karwowska M, Kononiuk A (2020) Nitrates/nitrites in food – risk for nitrosative stress and benefits. Antioxidants (Basel) 9:241. 10.3390/antiox9030241 PMC7139399 32188080

[CR224] Kasha KJ, Maluszynski M (2003) Production of doubled haploids in crop plants. An introduction. In: Maluszynski M, Kasha KJ, Forster BP, Szarejko I (eds) Doubled haploid production in crop plants. Springer, Dordrecht, the Netherlands, pp 1–4. 10.1007/978-94-017-1293-4

[CR225] Kaur C, Kapoor HC (2002) Antioxidant activity and total phenolic content of some Asian vegetables. Int J Food Sci Technol 37:153–161

[CR226] Kelebek H, Selli S, Canbas A, Cabaroglu T (2009) HPLC determination of organic acids, sugars, phenolic compositions and antioxidant capacity of orange juice and orange wine made from a Turkish cv. Kozan. Microchem J 91:187–192. 10.1016/j.microc.2008.10.008

[CR227] Keser S, Celik S, Turkoglu S, Yilmaz O, Turkoglu I (2012) Hydrogen peroxide radical scavenging and total antioxidant activity of hawthorn. Chem J 2:9–12

[CR228] Khadivi A, Mirheidari F, Moradi Y (2022) Selection of superior accessions of turnip (*Brassica rapa* var. *rapa* L.) based on tuber quality-related characters. Food Sci Nutr 10:2667–2680. 10.1002/fsn3.2871 PMC9361458 35959251

[CR229] Kim SH, Ahn YO, Ahn MJ, Jeong JC, Lee HS, Kwak SS (2013) Cloning and characterization of an orange gene that increases carotenoid accumulation and salt stress tolerance in transgenic sweetpotato cultures. Plant Physiol Biochem 70:445–454. 10.1016/j.plaphy.2013.06.011 23835362

[CR230] Kim TH, Lee KH, Chung MN, Lee HU, Nam S-S, Park W (2025) Identification of single nucleotide polymorphisms and candidate genes associated with fiber content in sweetpotato (*Ipomoea batatas* (L.) Lam.) through a genome-wide association study. BMC Plant Biol 25:569. 10.1186/s12870-025-06614-7 PMC12044718 40307682

[CR231] Kimura M, Kobori CN, Rodriguez-Amaya DB, Nestel P (2005) Screening and HPLC methods for carotenoids in sweet potato, cassava and maize for plant breeding trials. Food Chem 100:1734–1746. 10.1016/j.foodchem.2005.10.020

[CR232] Kliebenstein DJ, Kroymann J, Brown P, Figuth A, Pedersen D, Gershenzon J, Mitchell-Olds T (2001) Genetic control of natural variation in *Arabidopsis* glucosinolate accumulation. Plant Physiol 126:811–825. 10.1104/pp.126.2.811 PMC111171 11402209

[CR233] Klopsch R, Witzel K, Börner A, Schreiner M, Hanschen FS (2017) Metabolic profiling of glucosinolates and their hydrolysis products in a germplasm collection of *Brassica rapa* turnips. Food Res Int 100:392–403. 10.1016/j.foodres.2017.04.016 28964362

[CR234] Kobayashi H, Shirasawa K, Fukino N, Hirakawa H, Akanuma T, Kitashiba H (2020) Identification of genome-wide single-nucleotide polymorphisms among geographically diverse radish accessions. DNA Res 27:dsaa001. 10.1093/dnares/dsaa001 PMC7315352 32065621

[CR235] Koh E, Charoenprasert S, Mitchell AE (2012) Effect of organic and conventional cropping systems on ascorbic acid, vitamin C, flavonoids, nitrate, and oxalate in 27 varieties of spinach (*Spinacia oleracea* L.). J Agric Food Chem 60:3144–3150 10.1021/jf300051f 22393895

[CR236] Kreutzmann S, Christensen LP, Edelenbos M (2008) Investigation of bitterness in carrots (*Daucus carota* L.) based on quantitative chemical and sensory analyses. LWT-Food Sci Technol 41:193–205. 10.1016/j.lwt.2007.02.024

[CR237] Kumar R, Bohra A, Pandey AK, Pandey MK, Kumar A (2017) Metabolomics for plant improvement: status and prospects. Front Plant Sci 8:1302. 10.3389/fpls.2017.01302 PMC5545584 28824660

[CR238] Kundu R, Majumder A, Pal S (2012) Evaluation of potato cultivars against arsenic accumulation under an arsenic-contaminated zone of eastern India. Potato J 39:62–68

[CR239] Kurina AB, Kornyukhin DL, Solovyeva AE, Artemyeva AM (2021) Genetic diversity of phenotypic and biochemical traits in VIR radish (Raphanus sativus L) germplasm collection. Plants 10(1):1799 10.3390/plants10091799 PMC8468841 34579332

[CR240] Laurie SM, Naidoo SIM, Magwaza L, Shimelis H, Laing M (2020) Assessment of the genetic diversity of sweetpotato germplasm collections for protein content. S Afr J Bot 132:132–139. 10.1016/j.sajb.2020.03.041

[CR241] Le Clerc V, Aubert C, Cottet V, Yovanopoulos C, Piquet M, Suel A, Huet S, Koutouan C, Hamama L, Chalot G, Jost M, Pumo B, Briard M (2019) Breeding for carrot resistance to *Alternaria dauci* without compromising taste. Mol Breed 39:59. 10.1007/s11032-019-0966-7

[CR242] Lebot V, Champagne A, Malapa R, Shiley D (2009) NIR determination of major constituents in tropical root and tuber crop flours. J Agric Food Chem 57:10539–10547. 10.1021/jf902675n 19919112

[CR243] Lee JG, Bonnema G, Zhang N, Kwak JH, de Vos RCH, Beekwilder J (2013a) Evaluation of glucosinolate variation in a collection of turnip (*Brassica rapa*) germplasm by the analysis of intact and desulfo glucosinolates. J Agric Food Chem 61:3984–3993. 10.1021/jf400890p 23528157

[CR244] Lee R, Baldwin S, Kenel F, McCallum J, Macknight R (2013b) FLOWERING LOCUS T genes control onion bulb formation and flowering. Nat Commun 4:2884. 10.1038/ncomms3884 24300952

[CR245] Lee Y-R, Kim CW, Han J-W, Choi HJ, Han K, Lee ES, Kim D-S, Lee J, Siddique MI, Lee H-E (2021) Genotyping-by-sequencing derived genetic linkage map and quantitative trait loci for sugar content in onion (*Allium cepa* L.). Plants (Basel) 10:2267. 10.3390/plants10112267 PMC8625195 34834630

[CR149] Folch, J., Lees, M., & Sloane, G. H. (1957). A simple method for the isolation and purification of total lipids from animal tissues. J Biol Chem 226, 497–509. https:// doi.org/10.1016/S0021- 9258(18)64849–5 13428781

[CR246] Leidi EO, Altamirano AM, Mercado G, Rodriguez JP, Ramos A, Alandia G, Sørensen M, Jacobsen S-E (2018) Andean roots and tubers crops as sources of functional foods. J Funct Foods 51:86–93. 10.1016/j.jff.2018.10.007

[CR247] Li HS (2000) Experimental principles and techniques of plant physiology and biochemistry. Higher Education Press, Beijing, China

[CR248] Li J, Yu X, Zhang S, Yu Z, Li J, Jin X, Zhang X, Yang D (2021) Identification of starch candidate genes using SLAF-seq and BSA strategies and development of related SNP-CAPS markers in tetraploid potato. PLoS ONE 16:e0261403. 10.1371/journal.pone.0261403 PMC8691606 34932571

[CR256] Li Y, Tan C, Li Z, Guo J, Li S, Chen X, Wang C, Dai X, Yang H, Song W, Hou L, Xu J, Tong Z, Xu A, Yuan X, Wang W, Yang Q, Chen L, Sun Z, Wang K, Pan B, Chen J, Bao Y, Liu F, Qi X, Gang DR, Wen J (2022) The genome of *Dioscorea zingiberensis* sheds light on the biosynthesis, origin and evolution of the medicinally important diosgenin saponins. Hortic Res 9:uhac165. 10.1093/hr/uhac165 PMC9531337 36204203

[CR249] Li X, Cui L, Zhang L, Huang Y, Zhang S, Chen W, Deng X, Jiao Z, Yang W, Qiu Z, Yan C (2023) Genetic diversity analysis and core germplasm collection construction of radish cultivars based on structure variation markers. Int J Mol Sci 24:2554. 10.3390/ijms24032554 PMC9916615 36768875

[CR250] Li H, Brouwer M, Pup ED, van Lieshout N, Finkers R, Bachem CWB, Visser RGF (2024a) Allelic variation in the autotetraploid potato: genes involved in starch and steroidal glycoalkaloid metabolism as a case study. BMC Genomics 25:274. 10.1186/s12864-024-10186-5 PMC10936075 38475714

[CR251] Li M, Xiong Y, Yang X, Gao Y, Li K (2024b) Transcriptomic and metabolic analysis reveals genes and pathways associated with flesh pigmentation in potato (*Solanum tuberosum*) tubers. Curr Issues Mol Biol 46:10335–10350. 10.3390/cimb46090615 PMC11430057 39329967

[CR257] Li N, Wang WW, Guo X, Sun YY, Li G, Li GG, Zhang S (2025a) *BvBZR1* improves parenchyma cell development and sucrose accumulation in sugar beet (*Beta vulgaris* L.) taproot. Front Plant Sci 16:1495161. 10.3389/fpls.2025.1495161 PMC11830749 39963534

[CR253] Li N, Zhang Y, Xing L, Ge M, Suo N (2025c) Genome-wide identification and expression profiling of plasma membrane-localized SWEET gene family associated with sugar transport during yam tuber development. Int J Mol Sci 26:5847. 10.3390/ijms26125847 PMC12193017 40565307

[CR252] Li N, Zhang Y, Huo X, Xing L, Ge M, Suo N (2025b) Genome-wide identification, plasma membrane localization, and functional validation of the SUT gene family in yam (*Dioscorea cayennensis* subsp. *rotundata*). Int J Mol Sci 26:5756. 10.3390/ijms26125756 PMC12193089 40565219

[CR255] Li Q, Wang Y, Gao Y, Wang Q, Niu Z, Nan X, Fan G, Sheng W (2025e) Comparative transcriptome analysis of genes related to tuberization in late and early maturing potato (*Solanum tuberosum*) cultivars. Sci Rep 15:26000. 10.1038/s41598-025-11875-1 PMC12271483 40676087

[CR254] Li X-J, Hao Y-C, Zheng J-W, Wang Y-H, Tian J-X, Zhang CH, Yan C-S, Zhou L, Song X-M, Xiong A-S, Liang Y (2025d) Analysis of telomere-to-telomere genome of red carrot TXH4 elucidates the role of *DcLCYE* and DcLCYB1 in lycopene accumulation in carrot. Hortic Res 12:uhaf192. 10.1093/hr/uhaf192 PMC12552772 41140879

[CR258] Lim S-H, Kim D-H, Lee J-Y (2022) RsTTG1, a WD40 protein, interacts with the bHLH transcription factor RsTT8 to regulate anthocyanin and proanthocyanidin biosynthesis in *Raphanus sativus*. Int J Mol Sci 23:11973. 10.3390/ijms231911973 PMC9570178 36233274

[CR259] Lim S-H, Kim D-H, Lee J-Y (2023) Molecular mechanism controlling anthocyanin composition and content in radish plants with different root colors. Plant Physiol Biochem 204:108091. 10.1016/j.plaphy.2023.108091 37864927

[CR260] Lin YT, Liang C, Chen JH (2011) Feasibility study of ultraviolet activated persulfate oxidation of phenol. Chemosphere 82:1168–1172. 10.1016/j.chemosphere.2010.12.027 21220146

[CR261] Lin Y, Qiu L, Zhang M, Zhang C, Qin Y, Yu H, Lin Q, Ge L (2025) Comprehensive evaluation on nutritional characteristics and anti-hyperglycemic active ingredients of different varieties of Yam. Sci Rep 15:12609. 10.1038/s41598-025-95401-3 PMC11993657 40221498

[CR262] Liu YM, Lin KW (2009) Antioxidative ability, dioscorinstability, and the quality of yam chips from various yam species as affected by processing method. J Food Sci 74:C118–C125 10.1111/j.1750-3841.2008.01040.x 19323725

[CR263] Liu WJ, Zhang Y, Zhang YX, Du GQ (2022) Analysis and evaluation of fruit quality and aroma components of ‘Yali’ Pear (*Pyrus bretschneideri* Rehd.) pollinated with eighteen pollinizers. Food Sci 43:294–302. 10.7506/spkx1002-6630-20210130-348

[CR264] Liu Y, Wang C, Chen H, Dai G, Cuimu Q, Shen W, Gao L, Zhu B, Gao C, Chen L, Chen D, Zhang X, Tan C (2024) Comparative transcriptome analysis reveals transcriptional regulation of anthocyanin biosynthesis in purple radish (*Raphanus sativus* L.). BMC Genomics 25:624. 10.1186/s12864-024-10519-4 PMC11188213 38902601

[CR265] Liu Z, Li Z, Li G, Qiao L (2025) Fine-mapping of a red-skinned taproot gene in radish (*Raphanus sativus* L.). Plants (Basel) 14:3065. 10.3390/plants14193065 PMC12525809 41095206

[CR266] Low J, Carvey E (2020) Maria Isabel Andrade: Sweet potato breeder, technology transfer specialist, and advocate. Plant Breed Rev 43:1–30. Wiley

[CR267] Lowry OH, Rosebrough NJ, Farr AL, Randall RJ (1951) Protein measurement with the Folin phenol reagent. J Biol Chem 193:265–275. 10.1016/S0021-9258(19)52451-6 14907713

[CR268] Ma Q, Tong W, Cheng L, Zhang Y, Wang Y, Wang X, Feng Y, Li R, Lu X, Yan W, Zhang P (2025) Advancing cassava molecular breeding through genome editing: a promising pathway. Trop Plants 4:e004. 10.48130/tp-0024-0046

[CR269] Magwaza LS, Naidoo SIM, Laurie SM, Laing MD, Shimelis H (2016) Development of NIRS models for rapid quantification of protein content in sweetpotato [*Ipomoea batatas* (L.) LAM.]. LWT-Food Sci Technol 72:63–70. 10.1016/j.lwt.2016.04.032

[CR270] Manikar P, Manape TK, Sathees V, Anandhan S (2023) CRISPR/Cas9-mediated editing of *PHYTOENE DESATURASE* gene in onion (*Allium cepa* L.). Front Plant Sci 14:1226911. 10.3389/fpls.2023.1226911 PMC10494252 37701798

[CR271] Maresca V, Capasso L, Rigano D, Stornaiulo M, Sirignano C, Piacente S, Cerulli A, Marallo N, Basile A, Nebbioso A, Giordano D, Facchiano A, De Masi L, Bontempo P (2024) Health-promoting effects, phytochemical constituents and molecular genetic profile of the purple carrot ‘Purple Sun’ (*Daucus carota* L.). Nutrients 16: 2505. 10.3390/nu16152505 PMC11314219 39125387

[CR272] Mascher M, Schreiber M, Scholz U, Graner A, Reif JC, Stein N (2019) Genebank genomics bridges the gap between the conservation of crop diversity and plant breeding. Nat Genet 51:1076–1081. 10.1038/s41588-019-0443-6 31253974

[CR273] Matsumura Y, Kitabatake M, Kayano S-I, Ito T (2023) Dietary phenolic compounds: their health benefits and association with the gut microbiota. Antioxidants (Basel) 12:880. 10.3390/antiox12040880 PMC10135282 37107256

[CR274] Matthewman MC, Costa-Pinto R (2023) Macronutrients, minerals, vitamins and energy. Anaesth Intensive Care 24:134–138. 10.1016/j.mpaic.2022.12.009

[CR275] Mazza G, Fukumoto L, Delaquis P, Girard B, Ewert B (1999) Anthocyanins, phenolics, and color of cabernet franc, merlot, and pinot noir wines from British Columbia. J Agric Food Chem 47:4009–4017. 10.1021/jf990449f 10552758

[CR276] Mbanjo EGN, Ogungbesan A, Agbona A, Akpotuzor P, Toyinbo S, Iluebbey P, Rabbi IY, Peteti P, Wages SA, Norton J, Zhang X, Bohórquez-Chaux A, Mushoriwa H, Egesi C, Kulakow P, Parkes E (2024) Validation of SNP markers for diversity analysis, quality control, and trait selection in a biofortified cassava population. Plants 13:2328. 10.3390/plants13162328 PMC11359368 39204764

[CR277] McCallum J, Pither-Joyce M, Shaw M, Kenel F, Davis F, Butler R, Scheffer J, Jakse J, Havey MJ (2007) Genetic mapping of sulfur assimilation genes reveals a QTL for onion bulb pungency. Theor Appl Genet 114:815–822. 10.1007/s00122-006-0479-8 17180376

[CR278] McCleary BV, Gibson TS, Solah V, Mugford DC (1994) Total starch measurement in cereal products: interlaboratory evaluation of a rapid enzymic test procedure. Cereal Chem 71:501–505

[CR279] McCleary BV, Gibson TS, Mugford DC (1997) Measurement of total starch in cereal products by amyloglucosidase-α-amylasemethod: Collaborative study. J AOAC Int 80:571–579

[CR280] Medina-Lozano I, Díaz A (2022) Applications of genomic tools in plant breeding: crop biofortification. Int J Mol Sci 23:3086. 10.3390/ijms23063086 PMC8950180 35328507

[CR281] Meng X, Liu S, Dong T, Xu T, Ma D, Pan S, Li Z, Zhu M (2020) Comparative transcriptome and proteome analysis of salt-tolerant and salt-sensitive sweet potato and overexpression of *IbNAC7* confers salt tolerance in *Arabidopsis*. Front Plant Sci 11:572540. 10.3389/fpls.2020.572540 PMC7481572 32973858

[CR282] Merga MA, Mourtala IZM, Abtew WG, Oselebe HO (2025) Genetic diversity and nutritional analysis of sweet potato [*Ipomoea batatas* (l.) Lam.] genotypes in Abakaliki, Nigeria. BMC Plant Biol 25:548. 10.1186/s12870-025-06558-y PMC12036219 40295923

[CR283] Modak M, Akter MM, Hasi MA, Trisha SA, Muhimine AAI, Rahman EHMS, Robin AHK (2025) Genetic diversity and trait associations in biofortified short-duration sweet potato (*Ipomoea batatas* L.) genotypes. BMC Plant Biol 26:119. 10.1186/s12870-025-07754-6 PMC12825249 41413456

[CR284] Modi AT (2025) Nutritional value of *Colocasia esculenta* is related to corm size. Life (Basel) 15:1712. 10.3390/life15111712 PMC12653675 41302137

[CR285] Moeinizade S, Kusmec A, Hu G, Wang L, Schnable PS (2020) Multi-trait genomic selection methods for crop improvement. Genetics 215:931–945. 10.1534/genetics.120.303305 PMC7404246 32482640

[CR286] Mohamedshah Z, Chadwick-Corbin S, Wightman JD, Ferruzzi MG (2020) Comparative assessment of phenolic bioaccessibility from 100% grape juice and whole grapes. Food Funct 11:6433–6445. 10.1093/cdn/nzaa045_073 32613989

[CR287] Molyneux P (2004) The use of the stable free radical diphenylpicrylhydrazyl (DPPH) for estimating antioxidant activity. Songklanakarin J Sci Technol 26:211–219

[CR288] Mondo JM, Balezi AZ, Ishara J, Mwanga Mwanga J-CI, Matendo ER, Bagaya D-GN, Chuma GB, Agre PA, Adebola P, Asfaw A (2025) Nutritional and health benefits of wild and cultivated yam (*Dioscorea* spp.) species consumed in eastern Democratic Republic of Congo. Sci Rep 15:42131. 10.1038/s41598-025-26046-5 PMC12658234 41298666

[CR289] Montagnac JA, Davis CR, Tanumihardjo SA (2009) Nutritional value of cassava for use as a staple food and recent advances for improvement. Compr Rev Food Sci Food Saf 8:181–194 10.1111/j.1541-4337.2009.00077.x 33467798

[CR290] Moreira FF, Oliveira HR, Volenec JJ, Rainey KM, Brito LF (2020) Integrating high-throughput phenotyping and statistical genomic methods to genetically improve longitudinal traits in crops. Front Plant Sci 11:681. 10.3389/fpls.2020.00681 PMC7264266 32528513

[CR291] Morris WL, Ducreux L, Griffiths DW, Stewart D, Davies HV, Taylor MA (2004) Carotenogenesis during tuber development and storage in potato. J Exp Bot 55:975–982. 10.1093/jxb/erh121 15047766

[CR292] Moses RJ, Edo GI, Jikah AN, Emakpor OL, Agbo JJ (2025) Cassava consumption and the risk from cyanide poisoning. Vegetos 38:1713–1724. 10.1007/s42535-024-01121-w

[CR293] Mota APZ, Dossa K, Lechaudel M, Cornet D, Mournet P, Santoni S, Lopez D, Chaïr H (2024) Whole genome sequencing and comparative genomics reveal candidate genes associated with quality traits in *Dioscorea alata*. BMC Genomics 25:248. 10.1186/s12864-024-10135-2 PMC10916269 38443859

[CR294] Mudihal T, Cholin S, Kulkarni CC, Shivaprasad KM, Kumar P, Rolling W, Simon P (2025) Exploring functional InDels and genetic diversity: agro-morphometric and molecular insights into the Western and Eastern gene pools of carrot (Daucus carota L). Front Plant Sci 16:1658653 10.3389/fpls.2025.1658653 PMC12587770 41200494

[CR295] Mushtaq MW, Tariq S, Ahmad A, Bashir S (2022) Spectrophotometric determination of vitamin C in underground vegetables and kinetic modelling to probe the effect of temperature and pH on degradation of vitamin C. Pak J Bot 54:1771–1775. 10.30848/PJB2022-5(12)

[CR296] Mwanga ROM, Swanckaert J, da Silva Pereira G, Andrade MI, Makunde G, Grüneberg WJ, Kreuze J, David M, De Boeck B, Carey E, Ssali RT, Utoblo O, Gemenet D, Anyanga MO, Yada B, Chelangat DM, Oloka B, Mtunda K, Chiona M, Koussao S, Laurie S, Campos H, Yencho GC, Low JW (2021) Breeding progress for vitamin A, iron and zinc biofortification, drought tolerance, and sweetpotato virus disease resistance in sweetpotato. Front Sustain Food Syst 5:616674. 10.3389/fsufs.2021.616674

[CR297] Nabot M, Garcia C, Seguin M, Ricci J, Brabet C, Remize F (2024) Bioactive compound diversity in a wide panel of sweet potato (*Ipomoea batatas* L.) cultivars: a resource for nutritional food development. Metabolites 14:523. 10.3390/metabo14100523 PMC11509433 39452904

[CR298] Naconsie M, Zhang P (2016) Transgenic technologies in cassava for nutritional improvement and viral disease resistance: a key strategy for food security in Africa. Front Agric Sci Eng 3:285. 10.15302/j-fase-2016119

[CR299] Nakayasu M, Akiyama R, Lee HJ, Osakabe K, Osakabe Y, Watanabe B, Sugimoto Y, Umemoto N, Saito K, Muranaka T, Mizutani M (2018) Generation of α-solanine-free hairy roots of potato by CRISPR/Cas9 mediated genome editing of the *St16DOX* gene. Plant Physiol Biochem 131:70–77. 10.1016/j.plaphy.2018.04.026 29735370

[CR300] Nambisan B (2011) Strategies for elimination of cyanogens from cassava for reducing toxicity and improving food safety. Food Chem Toxicol 49:690–693. 10.1016/j.fct.2010.10.035 21074593

[CR301] Narayanan N, Beyene G, Chauhan RD, Gaitán-Solís E, Gehan J, Butts P, Siritunga D, Okwuonu I, Woll A, Jiménez-Aguilar DM, Boy E, Grusak MA, Anderson P, Taylor NJ (2019) Biofortification of field-grown cassava by engineering expression of an iron transporter and ferritin. Nat Biotechnol 37:144–151. 10.1038/s41587-018-0002-1 PMC6784895 30692693

[CR302] Narayanan N, Beyene G, Chauhan RD, Grusak MA, Taylor NJ (2021) Stacking disease resistance and mineral biofortification in cassava varieties to enhance yields and consumer health. Plant Biotechnol J 19:844–854. 10.1111/pbi.13511 PMC8051606 33190345

[CR304] Nduche MU, Brehm JM, Abberton M, Faloye B, Maxted N (2024) Global genepool conservation and use strategy for *Dioscorea* (yam). Biodivers Conserv 33:4155–4176. 10.1007/s10531-024-02944-4

[CR305] Nie H, Park H, Kim S, Kim D, Kim S, Kwon S-Y, Kim S-H (2023) Genetic diversity assessment and genome-wide association study reveal candidate genes associated with component traits in sweet potato (*Ipomoea batatas* (L.) Lam). Mol Genet Genom 298:653–667. 10.1007/s00438-023-02007-3 36943475

[CR306] Nigussie M (2025) Nutritional and anti-nutritional quality of taro (*Colocasia esculenta*): a review. Innovation (Abingdon) 6:8–14. 10.11648/j.innov.20250601.12

[CR307] Ochar K, Ki D-W, Moon S, Bissah MN, Kim S-H (2026) Quantitative analysis of root system architecture and fresh weight biomass traits highlight phenotypic variation in radish (*Raphanus sativus* L.) germplasm. Sci Rep 16:8001. 10.1038/s41598-026-39212-0 PMC12957521 41663731

[CR308] Okoma PM, Kayondo SS, Rabbi IY, Amaefula C, de Andrade LRB, Jiwuba LC, Onyeka J, Egesi C, Jannick JL (2026) Genetic basis of cassava (*Manihot esculenta* Crantz) plant architecture and its relevance for selection of farmer-preferred varieties. 10.64898/2026.02.11.705251

[CR309] Okwuonu IC, Narayanan NN, Egesi CN, Taylor NJ (2021) Opportunities and challenges for biofortification of cassava to address iron and zinc deficiency in Nigeria. Glob Food Sec 28:100478. 10.1016/j.gfs.2020.100478

[CR310] Oladimeji JJ, Abe A, Kumar PL, Agre PA, Ilesanmi OJ, Vetukuri RR, Bhattacharjee R (2024) Extent and patterns of morphological and molecular genetic diversity and population structure of Nigerian taro cultivars. BMC Plant Biol 24:1077. 10.1186/s12870-024-05791-1 PMC11562719 39538118

[CR311] Olayide P, Alexandersson E, Tzfadia O, Lenman M, Gisel A, Stavolone L (2023) Transcriptome and metabolome profiling identify factors potentially involved in pro-vitamin A accumulation in cassava landraces. Plant Physiol Biochem 199:107713. 10.1016/j.plaphy.2023.107713 37126903

[CR313] Onoda K, Kato M, Tsunematsu Y, Eto F, Sato M, Yoshioka Y, Yoshida T, Tamura K, Yao I, Dohra H, Watanabe K (2023) Biosynthetic gene expression and tissue distribution of diosgenin in *Dioscorea japonica*. J Agric Food Chem 71:4292–4297. 10.1021/acs.jafc.2c08478 36753603

[CR314] Ouattara F, Agre PA, Adejumboi II, Olasanmi B, Stanley A, Sorho F, Dibi KEB, Akoroda MO, Bhattacharjee R (2026) Genome-wide association study revealed genomic regions associated with tuber quality traits in water yam (Dioscorea alata L). PLoS ONE. 10.1371/journal.pone.0339978 PMC12871974 41637406

[CR315] Ouédraogo N, Sombié PAED, Traoré RE, Sama H, Kando PB, Sawadogo M, Lebot V (2022) Nutritional and phytochemical characterization of taro [*Colocasia esculenta* (L.) Schott] germplasm from Burkina Faso. J Plant Breed Crop Sci 15:32–41. 10.5897/JPBCS2022.0999

[CR316] Padhan B, Nayak JK, Panda D (2018) Natural antioxidant potential of selected underutilized wild yams (*Dioscorea* spp.) for health benefit. J Food Sci Technol 57:2370–2376. 10.1007/s13197-020-04470-x PMC7230097 32431363

[CR317] Pandey J, Scheuring DC, Koym JW, Coombs J, Novy RG, Thompson AL, Holm DG, Douches DS, Miller C Jr, Vales IV (2021) Genetic diversity and population structure of advanced clones selected over forty years by a potato breeding program in the USA. Sci Rep 11:8344. 10.1038/s41598-021-87284-x PMC8052460 33863959

[CR318] Pandey J, Gautam S, Scheuring DC, Koym JW, Vales MI (2023) Variation and genetic basis of mineral content in potato tubers and prospects for genomic selection. Front Plant Sci 14:1301297. 10.3389/fpls.2023.1301297 PMC10766833 38186596

[CR319] Park S-C, Kim SH, Park S, Lee H-U, Lee JS, Park WS, Ahn M-J, Kim Y-H, Jeong JC, Lee H-S, Kwak S-S (2015) Enhanced accumulation of carotenoids in sweet potato plants overexpressing *IbOr-**Ins* gene in purple-fleshed sweet potato cultivar. Plant Physiol Biochem 86:82–90. 10.1016/j.plaphy.2014.11.017 25438140

[CR320] Pasqualone A, Punzi R, Trani A, Summo C, Paradiso VM, Caponio F, Gambacorta G (2017) Enrichment of fresh pasta with antioxidant extracts obtained from artichoke canning by-products by ultrasound-assisted technology and quality characterisation of the end product. Int J Food Sci Technol 52:2078–2087. 10.1111/ijfs.13486

[CR321] Pasupula K, Verma P, Zimik M, Kaur C, Vasudev S, Khar A (2024) Morphological, biochemical and molecular characterization of short-day tropical Indian garlic (*Allium sativum* L.). Heliyon 10:e37553 10.1016/j.heliyon.2024.e37553 PMC11414556 39309863

[CR322] Pati K, Giri AK, Chauhan VBS, Gowda KH, Arutselvan R, Nedunchezhiyan M, Laxminarayana K (2025) Sweet potato (*Ipomoea batatas* (L.) Lam) genetic resources. In: Kalia P (ed) Vegetable crops. Springer, Singapore, pp 1461–1473. 10.1007/978-981-97-8949-8

[CR323] Paul S, Geng C-A, Yang T-H, Yang Y-P, Chen J-J (2019) Comparative study of the glucosinolate profiles in turnip from four agroclimatic zones of China and neighbouring countries. Food Measure 13:2798–2811. 10.1007/s11694-019-00200-w

[CR324] Pelikan A, Goldman IL (2024) Variation for QTL alleles associated with total dissolved solids among crop types in a GWAS of a *Beta vulgaris* diversity panel. Plant Genome 18:e70014. 10.1002/tpg2.70014 PMC11897936 40071467

[CR325] Pereira GS, Amankwaah VA, Ketavi M, Oloka BM, Nair AGH, Mata APd, Silva CCd, Santos IGd, Ricardo J, Rosa BF, Campos H (2023) Sweetpotato: nutritional constituents and genetic composition. In: Kole C (ed) Compendium of crop genome designing for nutraceuticals. Springer, Singapore, pp 1545–1587. 10.1007/978-981-19-4169-6_58

[CR327] Petropoulos SA, Fernandes A, Ntatsi G, Petrotos K, Barros L, Ferreira ICFR (2018b) Nutritional value, chemical characterization and bulb morphology of Greek garlic landraces. Molecules 23:319. 10.3390/molecules23020319 PMC6017064 29393882

[CR326] Petropoulos S, Fernandes Â, Barros L, Ciric A, Sokovic M, Ferreira ICFR (2018a) Antimicrobial and antioxidant properties of various Greek garlic genotypes. Food Chem 245:7–12. 10.1016/j.foodchem.2017.10.078 29287429

[CR328] Plantenga FDM, Bergonzi S, Abelenda JA, Bachem CWB, Visser RGF, Heuvelink E, Marcelis LFM (2019) The tuberization signal StSP6A represses flower bud development in potato. J Exp Bot 70:937–948. 10.1093/jxb/ery420 30481308

[CR329] Pradhan B, Biswas M, Panda D (2020) Nutritional, anti-nutritional and physico-functional properties of wild edible yam (*Dioscorea* spp.) tubers from Koraput, India. Food Biosci 34:100527. 10.1016/j.fbio.2020.100527

[CR330] Price ML, Van Scoyoc S, Butler LG (1978) A critical evaluation of the vanillin reaction as an assay for tannin in sorghum grain. J Agric Food Chem 26:1214–1218. 10.1021/jf60219a031

[CR331] Qiao Q, Sheng F, Qiao W, Li S, Wang L, Xiao D, He L (2025) Genome-wide characterization of *DrRALF* genes in yam (*Dioscorea rotundata*) reveals their potential roles in tuber expansion and the gibberellin response. Int J Mol Sci 26:6151. 10.3390/ijms26136151 PMC12249873 40649928

[CR332] Rabbi IY, Udoh LI, Wolfe M, Parkes EY, Gedil MA, Dixon A, Ramu P, Jannink J-L, Kulakow P (2017) Genome-wide association mapping of correlated traits in cassava: Dry matter and total carotenoid content. Plant Genome 10:10.3835/plantgenome2016.09.0094. 10.3835/plantgenome2016.09.009PMC7822061 29293815

[CR333] Rasheed H, Deng B, Ahmad D, Bao J (2024) Genetic diversity and genome-wide association study of total phenolics, flavonoids, and antioxidant properties in potatoes (*Solanum tuberosum* L.). Int J Mol Sci 25:12795. 10.3390/ijms252312795 PMC11640895 39684503

[CR334] Rasheed H, Ying Y, Ahmad D, Deng B, Bao J (2025) Genotypic diversity and genome-wide association study of protein content and amino acid profile in diverse potato accessions. Foods 14:2039. 10.3390/foods14122039 PMC12192551 40565648

[CR335] Ratsimbazafy MK, Sharp PA, Razanamparany L, Rajaonah MT, Rakotoarison F, Khoja KK, Wilkin P, Howes M-JR (2023) Wild edible yams from Madagascar: new insights into nutritional composition support their use for food security and conservation. Food Sci Nutr 12:280–291. 10.1002/fsn3.3757 PMC10804101 38268876

[CR336] Raza A, Salehi H, Bashir S, Tabassum J, Jamla M, Charagh S, Barmukh R, Mir RA, Bhat BA, Javed MA, Guan DX, Mir RR, Siddique KHM, Varshney RK (2024) Transcriptomics, proteomics, and metabolomics interventions prompt crop improvement against metal(loid) toxicity. Plant Cell Rep 43:80. 10.1007/s00299-024-03153-7 PMC10899315 38411713

[CR337] Razzaq A, Sadia B, Raza A, Khalid Hameed M, Saleem F (2019) Metabolomics: a way forward for crop improvement. Metabolites 9:303. 10.3390/metabo9120303 PMC6969922 31847393

[CR338] Razzaq A, Tang Y, Qing P (2021) Towards sustainable diets: understanding the cognitive mechanism of consumer acceptance of biofortified foods and the role of nutrition information. Int J Environ Res Public Health 18:1175 10.3390/ijerph18031175 PMC7908173 33525742

[CR339] Ricciardi L, Mazzeo R, Marcotrigiano AR, Iovieno P, Zonno V, Pavan S, Rainaldi G, Lotti C (2020) Assessment of genetic diversity of the “Acquaviva Red Onion” (*Allium cepa* L.) Apulian landrace. Plants 9:260. 10.3390/plants9020260 PMC7076509 32085407

[CR340] Roca WM, Ynoupe C, Manrique I, Arbizu C, Gomez R (2007) Indigenous Andean root and tuber crops: new foods for the new millennium. Chron Hortic 47:13–19

[CR341] Rodriguez E, Rosero A, Perez JI, Garavito L, González JC, Alarcón K, Morante N, Salazar S, Belalcazar J, Ceballos H (2025) Improvement of cassava varieties for high nutritional quality adapted to the Pacific and Andean regions in Colombia. Plants 14:3762. 10.3390/plants14243762 PMC12736947 41470643

[CR342] Rolling WR, Ellison S, Coe K, Lorizzo M, Dawson J, Senalik D, Simon PW (2025) Combining genome-wide association and genomic prediction to unravel the genetic architecture of carotenoid accumulation in carrot. Plant Genome 18:e20560. 10.1002/tpg2.20560 PMC11782711 39887573

[CR343] Rosero A, Pastrana I, Martínez R, Perez J-L, Espitia L, Araujo H, Belalcazar L, Granda L, Jaramillo A (2022) Nutritional value and consumer perception of biofortified sweet potato varieties. Ann Agric Sci 67:79–89. 10.1016/j.aoas.2022.05.004

[CR344] Roullier C, Benoit L, McKey DB, Lebot V (2013) Historical collections reveal patterns of diffusion of sweet potato in Oceania obscured by modern plant movements and recombination. Proc Natl Acad Sci USA 110:2205–2210. 10.1073/pnas.1211049110 PMC3568323 23341603

[CR312] Omaye, S. T., Turnbull, T. D., & Sauberlich, H. E. (1979). Selected method for the determination of ascorbic acid in animal cells, tissues and fluid. In D. B. Mc Cormic, & D. L. Wright (Vol. Eds.), Methods in enzymology: Vol. 62, (pp. 3–11). New York, NY. 10.1016/0076-6879(79)62181-x 440112

[CR345] Samal A, Martin OC (2017) Haldane, Wadington and recombinant inbred lines: extension of their work to any number of genes. J Genet 96:795–800. 10.1007/s12041-017-0837-0 29237888

[CR346] Sanchez-Echaniz J, Benito-Fernández J, Mintegui-Raso S (2001) Methemoglobinemia and consumption of vegetables in infants. Pediatrics 107:1024–1028. 10.1542/peds.107.5.1024 11331681

[CR347] Sanders LM, Dicklin MR, Palacios OM, Maki CE, Wilcox ML, Maki KC (2020) Effects of potato resistant starch intake on insulin sensitivity, related metabolic markers and appetite ratings in men and women at risk for type 2 diabetes: a pilot cross‐over randomised controlled trial. J Hum Nutr Diet 34:94–105. 10.1111/jhn.12822 PMC7894332 33119948

[CR348] Sanginga N (2015) Root and tuber crops (cassava, yam, potato and sweet potato). Background Paper. In: Mbabu A (co-convener) Feeding Africa: An Action Plan for African Agricultural Transformation. Dakar, Senegal; 21–23 October 2015. African Development Bank – African Union – United Nations Commission for Africa, Abidjan, Côte d’Ivoire. https://www.afdb.org/fileadmin/uploads/afdb/Documents/Events/DakAgri2015/Root_and_Tuber_Crops__Cassava__Yam__Potato_and_Sweet_Potato_.pdf [Access: 22^nd^ February 2026]

[CR349] Santos CCD, Andrade LRBd, Carmo CDd, Oliveira EJ (2023) Development of cassava core collections based on morphological and agronomic traits and SNPS markers. Front Plant Sci 14:1250205. 10.3389/fpls.2023.1250205 PMC10511765 37745996

[CR351] Schierle J, Pietsch B, Ceresa A, Fizet C, Waysek EH (2004) Method for the determination of β-carotene in supplements and raw materials by reversed-phase liquid chromatography: single laboratory validation. J AOAC Int 87:1070–1082 PMC2586117 15493663

[CR352] Schönhals EM, Ding J, Ritter E, Paulo MJ, Cara N, Tacke E, Hofferbert H-R, Lübeck J, Strahwald J, Gebhardt C (2017) Physical mapping of QTL for tuber yield, starch content and starch yield in tetraploid potato (*Solanum tuberosum* L.) by means of genome-wide genotyping by sequencing and the 8.3 K SolCAP SNP array. BMC Genomics 18:642. 10.1186/s12864-017-3979-9 PMC5567664 28830357

[CR353] Schwimmer S, Weston WJ (1961) Onion flavor and odor, enzymatic development of pyruvic acid in onion as a measure of pungency. J Agric Food Chem 9:301–304

[CR354] Sekin D, Oku S, Yamanouchi U, Mizubayashi T (2025) Identification of a common major quantitative trait locus controlling onion bulb weight and development of molecular markers for bulb weight selection. Mol Breed 45:86. 10.1007/s11032-025-01614-9 PMC12553687 41146899

[CR355] Selvaraj MG, Montoya-P ME, Atanbori J, French AP, Pridmore T (2019) A low-cost aeroponic phenotyping system for storage root development: unravelling the below-ground secrets of cassava (*Manihot esculenta*). Plant Methods 15:131. 10.1186/s13007-019-0517-6 PMC6842211 31728153

[CR356] Selvaraj MG, Valderrama M, Guzman D, Valencia M, Ruiz H, Acharjee A (2020) Machine learning for high-throughput field phenotyping and image processing provides insight into the association of above and below-ground traits in cassava (*Manihot esculenta* Crantz). Plant Methods 16:87. 10.1186/s13007-020-00625-1 PMC7296968 32549903

[CR357] Semwal P, Painuli S, Abu-Izneid T, Rauf A, Sharma A, Daştan SD, Kumar M, Alshehri MM, Taheri Y, Das R, Mitra S, Emran TB, Sharifi-Rad J, Calina D, Cho WC (2022) Diosgenin: an updated pharmacological review and therapeutic perspectives. Oxid Med Cell Longev. 10.1155/2022/1035441 PMC9168095 35677108

[CR358] Seth R, Fiecke C, Ferruzzi MG, Ma G, Lorizzo M (2025) Characterization of anthocyanins, phenolics, and flavonoids in a global carrot collection through application of chemometrics and FT-NIR spectroscopy. Food Chem: X 29:102807. 10.1016/j.fochx.2025.102807 PMC12305724 40735134

[CR359] Sharma A, Nagpal AK (2019) Contamination of vegetables with heavy metals across the globe: hampering food security goal. J Food Sci Technol 57:391–403. 10.1007/s13197-019-04053-5 PMC7016143 32116349

[CR360] Sharma S, Holme IB, Dionisio G, Kodama M, Dzhanfezova T, Joernsgaard B, Brinch-Pedersen H (2020) Cyanidin based anthocyanin biosynthesis in orange carrot is restored by expression of Am Rosea1 and Am Delila, MYB and bHLH transcription factors. Plant Mol Biol 103:443–456. 10.1007/s11103-020-01002-1 32270430

[CR485] Sharma S, Dionisio G, Holme IB, Dzhanfezova T, Joernsgaard B, Brinch-Pedersen H (2021) Anthocyanin synthesis in orange carrot cv. Danvers is activated by transgene expression of the transcription factors DcMYB113_NB and DcEGL1_NB from black carrot cv. Nightbird. Plant Mol Biol 10:259-270. 10.1007/s11103-021-01141-z 33837502

[CR361] Shi L, de Biolley L, Shaikh MA, de Vries ME, Mittmann SU, Visser RGF, Prat S, Bachem CWB (2024) Aging later but faster: how *StCDF1* regulates senescence in *Solanum tuberosum*. New Phytol 242:2541–2554. 10.1111/nph.19525 38197194

[CR362] Siadjeu C, Mayland-Quellhorst E, Albach DC (2018) Genetic diversity and population structure of trifoliate yam (*Dioscorea dumetorum* Kunth) in Cameroon revealed by genotyping-by-sequencing (GBS). BMC Plant Biol 18:359. 10.1186/s12870-018-1593-x PMC6299658 30563456

[CR363] Singh BK, Koley TK, Karmakar P, Tripathi A (2017) Pigmented radish (*Raphanus sativus*): genetic variability, heritability and inter-relationships of total phenolics, anthocyanins and antioxidant activity. Indian J Agric Sci 87:1600–1606. 10.56093/ijas.v87i12.76484

[CR364] Singh PR, Mahajan V, Verma A, Sagar R, Jayaswall K, Shukla N, Gedam P, Singh M (2021) Identification and characterization of white onion (*Allium cepa* L.) genotypes for high total soluble solid content through molecular markers. Genet Resour Crop Evol 68:1–11. 10.1007/s10722-020-01037-9

[CR367] Singh B, Sharma J, Bhardwaj V, Sood S, Sidappa S, Gautam U, Dalamu KHB, Kumar D, Kumar V (2022c) Genotypic variations for tuber nutrient content, dry matter and agronomic traits in tetraploid potato germplasm. Physiol Mol Biol Plants 28:1233–1248. 10.1007/s12298-022-01197-1 PMC9334495 35910435

[CR365] Singh D, Bisen M, Shukla R, Prabha R, Maurya S, Reddy YS, Singh PM, Rai N, Chaubey T, Chaturvedi KK, Srivastava S, Farooqi MS, Gupta VK, Sarma BK, Rai A, Behera TK (2022a) Metabolomics-driven mining of metabolite resources: applications and prospects for improving vegetable crops. Int J Mol Sci 23:12062. 10.3390/ijms232012062 PMC9603451 36292920

[CR366] Singh D, Dhillon TS, Javed T, Singh R, Dobaria J, Dhankhar SK, Kianersi F, Ali B, Poczai P, Kumar U (2022b) Exploring the genetic diversity of carrot genotypes through phenotypically and genetically detailed germplasm collection. Agronomy 12:1921. 10.3390/agronomy12081921

[CR368] Singleton VL, Rossi JA (1965) Colorimetry of total phenolics with phosphomolybdic-phosphotungstic acid reagents. Am J Enol Vitic 16:144–158

[CR369] Singleton VL, Orthofer R, Lamuela-Raventós RM (1999) Analysis of total phenols and other oxidation substrates and antioxidants by means of folin-ciocalteu reagent. Methods Enzymol 299:152–178

[CR370] Siviya LE, Figlan SM, Laurie ME, Rauwane ME (2025) Proteomic insights on root and tuber crops’ response to abiotic stress—a review. Crop Sci 65:6. 10.1002/csc2.70193

[CR371] Slonecki TJ, Rutter WWB, Olukolu BA, Yencho GC, Jackson DM, Wadl PA (2023) Genetic diversity, population structure, and selection of breeder germplasm subsets from the USDA sweet potato (*Ipomoea batatas*) collection. Front Plant Sci 13:1022555. 10.3389/fpls.2022.1022555 PMC9932972 36816486

[CR375] Sönmez I, Erol ÜH, Sarpkaya K (2025) Comprehensive morphological and biochemical characterization of garlic (*Allium sativum* L.) genotypes using multivariate analysis. BMC Plant Biol 25:856. 10.1186/s12870-025-06859-2 PMC12224609 40610852

[CR372] Sombié PAED, Hilou A, Mounier C, Coulibaly A, Kiendrebeogo M, Millogo JF, Nacoulma OG (2011) Antioxidant and anti-inflammatory activities from galls of *Guiera** senegalensis* J.F. GMEL (Combretaceae). Res J Med Plant 5:448–461

[CR373] Son J-H, Lee J-H, Choi W-Y, Park H-G, Kang S-H, Ha Y (2026) Automated garlic grading using deep learning segmentation: a comparative study of 2D and 3D imaging techniques. J Agric Food Res 28:102967. 10.1016/j.jafr.2026.102967

[CR374] Song W, Yan H, Ma M, Kou M, Li C, Tang W, Yu Y, Hao Q, Nguyen T, Wang X, Zhang Z, You C, Gao R, Zhang Y, Li Q (2022) Comparative transcriptome profiling reveals the genes involved in storage root expansion in sweet potato (*Ipomoea batatas* (L.) Lam.). Genes 13:1156. 10.3390/genes13071156 PMC9321896 35885939

[CR106] Dalamu, Sood S, Mangal V, Kumar V, Sharma A, Bhardwaj V (2025) Potato: collection, conservation, and evaluation of genetic resources. In: Kalia P (ed) Vegetable crops. Springer, Singapore, pp 143–178. 10.1007/978-981-97-8949-8

[CR376] Srivichai S, Hongsprabhas P (2020) Profiling anthocyanins in Thai purple yams (*Dioscorea alata* L.). Int J Food Sci 2020: Article ID 1594291. 10.1155/2020/1594291 PMC7368940 32695807

[CR377] Sun B, Ricardo-da-Silva JM, Spranger I (1998) Critical factors of vanillin assay for catechins and proanthocyanidins. J Agric Food Chem 46:4267–4274. 10.1021/jf980366j

[CR378] Sun S, Guo B, Wei Y, Fan M (2011) Multi-element analysis for determining the geographical origin of mutton from different regions of China. Food Chem 124:1151–1156. 10.1016/j.foodchem.2010.07.027

[CR379] Sun S-X, Li Y, Jia L, Ye S, Luan Y (2024) Identification of genetic variants controlling diosgenin content in *Dioscorea zingiberensis* tuber by genome-wide association study. BMC Plant Biol 24:540. 10.1186/s12870-024-05133-1 PMC11177481 38872080

[CR381] Sun J, Zhuo X, Chen X, Wei J, Lin J, Shang H, Shan N, Huang Y, Zhou Q, Li Z (2025b) Metabolome and transcriptome reveal the regulatory mechanism of anthocyanin synthesis in tuber skin and flesh of (Dioscorea alata L.). Plants (Basel) 14:3454. 10.3390/plants14223454 PMC12655603 41304606

[CR380] Sun M, Cui Y, Li J, Khan KS, Lyu J, Xiao X, Yu J (2025a) HS-SPME/GC–MS and *E*-nose-based characterization of volatile compounds in purple garlic across eight Chinese regions. Food Chem: X 30:102896. 10.1016/j.fochx.2025.102896 PMC12363586 40837091

[CR382] Sun Y, Wang Z, Wang H, Wang Y, Qian Z, Liu Y, Yang Y, Qin X, Huang G, Ying X, Song G, Zhu H (2026) Chromosome level genome assembly of taro (*Colocasia esculenta*). Sci Data 13:802. 10.1038/s41597-026-06737-y PMC13223283 41957018

[CR383] Swaroop S, Cargill EJ, Crosby K, Flagel L, Kniskern J, Glenn KC (2021) Genetic diversity is indispensable for plant breeding to improve crops. Crop Sci 61:839–852. 10.1002/csc2.20377

[CR384] Syombua ED, Zhang Z, Tripathi JN, Ntui VO, Kang M, George OO, Edward N, Wang, K, Yang B, Tripathi L (2021) A CRISPR/Cas9-based genome-editing system for yam (Dioscorea spp.). Plant Biotechnol J. 10.1111/pbi.13515 PMC8051594 33222361

[CR385] Tabussam N, Rana RM, Sah MKN, Ahmad MS, Sajjad M, Lu Y (2022) Nutraceutical profiling of elite onion germplasm and breeding hybrids with improved nutraceutical quality. PLoS ONE 17:e0262705. 10.1371/journal.pone.0262705 PMC8769310 35045129

[CR386] Tai HH (2024) Insights into the molecular regulation of source–sink relations in potato. New Phytol 242:2385–2387. 10.1111/nph.19705 38523243

[CR387] Talang H, Mawlong GT, Kjam L, Devi MB, Gurung B, Biswakarma N, Singh NU, Verma VK, Rymbai H, Raviteja P, Das B, Angami T, Yanthan AW, Patra S, Makdoh B, Ch Sangma RK, Assumi SR, Sangma CBK, Chanu LJ, Hazarika S (2025) Assessment of nutritional quality of *taro* (*Colocasia esculenta* L. Schott.) genotypes of the Eastern Himalaya, India. Front Nutr 12:1567829. 10.3389/fnut.2025.1567829 PMC12014443 40271429

[CR388] Talsma EF, Melse-Boonstra A, de Kok BPH, Mbera GN, Mwangi AM, Brouwer ID (2013) Biofortified cassava with pro-vitamin A is sensory and culturally acceptable for consumption by primary school children in Kenya. PLoS ONE 8:e73477. 10.1371/journal.pone.0073433 PMC3758265 24023681

[CR389] Tamboli FA, More HN, Bhandugare SS, Patil AS, Jadhav NR, Killedar SG (2020) Estimation of total carbohydrate content by phenol sulphuric acid method from *Eichhornia crassipes* (Mart.) Solms. Asian J Res Chem 13:357–359

[CR390] Tan H, Luo X, Lu J, Wu L, Li Y, Jin Y, Peng X, Xu X, Li J, Zhang W (2023) The long noncoding RNA *LINC15957* regulates anthocyanin accumulation in radish. Front Plant Sci 14:1139143. 10.3389/fpls.2023.1139143 PMC10009236 36923129

[CR391] Tare K, Kumar R, Kaushik K, Sharma L, Lamichaney S (2026) Multivariate and stability analysis for yield and biochemical traits in radish (Raphanus sativus L) genotypes from Sikkim Himalaya for functional food applications. Sci Rep. 10.1038/s41598-026-38280-6 PMC13018483 41714342

[CR392] Teixeira GG, Santos PM (2022) Simple and cost-effective approaches for quantification of reducing sugar exploiting digital image analysis. J Food Compos Anal 113:104719. 10.1016/j.jfca.2022.104719

[CR393] Temesgen M (2015) Nutritional potential, health and food security benefits of taro *Colocasia esculenta* (L.): a review. Food Sci Qual Manag 36:23–31

[CR394] Thakur P, Dhinman A, Kumar S, Suhag R (2024) Garlic (*Allium sativum* L.): A review on bio-functionality, allicin’s potency and drying methodologies. S Afr J Bot 171:129–146. 10.1016/j.sajb.2024.05.039

[CR395] Tolkki L, Alanko K, Petman L, Skydtsgaard MB, Milvang PG, Seppälä U, Ranki A (2013) Clinical characterization and IgE profiling of birch (*Betula verrucosa*) – allergic individuals suffering from allergic reactions to raw fruits and vegetables. J Allergy Clin Immunol Pract 1:623–631. 10.1016/j.jaip.2013.07.010 24565710

[CR396] Tomlins K, Ndunguru G, Stambul K, Joshua N, Ngendello T, Rwiza E, Amour R, Ramadhani B, Kapande A, Westby A (2007) Sensory evaluation and consumer acceptability of pale-fleshed and orange-fleshed sweetpotato by school children and mothers with preschool children. J Sci Food Agric 87:2436–2446. 10.1002/jsfa.2931

[CR397] Tońska E, Klepacka J, Michalak J, Toński M (2020) Lead and cadmium in conventional and organic carrots. Proc Nutr Soc. 10.1017/S0029665120002724

[CR398] Torabian S, Qin R, Sathuvalli S, Jayanty S (2023) Glycoalkaloids in potato tubers. Or State Univ Ext Serv EM 9407. https://extension.oregonstate.edu/catalog/pub/em9407

[CR399] Trani A, Verrastro V, Punzi R, Faccia M, Gambacorta G (2016) Phenols, volatiles and sensory properties of primitivo wines from the “Gioia Del Colle” PDO area. S Afr J Enol Vitic 37:139–148. 10.21548/37-2-1002

[CR400] Truong VD, Deighton N, Thompson RT, McFeeters RF, Dean LO, Pecota KV, Yencho GC (2010) Characterization of anthocyanins and anthocyanidins in purple-fleshed sweet potatoes by HPLC-DAD/ESI-MS/MS. J Agric Food Chem 58:404–410. 10.1021/jf902799a 20017481

[CR401] Uchendu K, Njoku DN, Paterne A, Rabbi IY, Dzidzienyo D, Tongoona P, Offei S, Egesi C (2021) Genome-wide association study of root mealiness and other texture-associated traits in cassava. Front Plant Sci 12:770434. 10.3389/fpls.2021.770434 PMC8719520 34975953

[CR402] Urfan M, Sharma S, Hakla HR, Rajput P, Andotra S, Lehana PK, Bhardwaj R, Khan MS, Das R, Kumar S, Pal S (2022) Recent trends in root phenomics of plant systems with available methods – discrepancies and consonances. Physiol Mol Biol Plants 28:1311–1321. 10.1007/s12298-022-01209-0 PMC9334470 35910442

[CR403] USDA (2025) Food Data Central. USDA’s comprehensive source of food composition data with multiple distinct data types. https://fdc.nal.usda.gov [Access: 22^nd^ February 2026]

[CR405] Vega A, Brainard SH, Goldman IL (2024) Linkage mapping of root shape traits in two carrot populations. G3 (Bethesda) 14:jkae041. 10.1093/g3journal/jkae041 PMC10989876 38412554

[CR406] Villwock SS, Parkes EY, Mbanjo EGN, Rabbi IR, Jannink J-L (2025) Mediated pleiotropy drives the negative correlation of total carotenoid and dry matter contents in cassava (*Manihot esculenta*). Genetics 231:iyaf162. 10.1093/genetics/iyaf162 PMC12505300 40796123

[CR407] Volk GM, Byrne PF, Coyne CJ, Flint-Garcia S, Reeves PA, Richards C (2021) Integrating genomic and phenomic approaches to support plant genetic resources conservation and use. Plants 10:2260. 10.3390/plants10112260 PMC8619436 34834625

[CR408] Vollmer R, Villagaray R, Castro M, Cáedenas J, Pineda S, Espirilla J, Anglin N, Ellis D, Azevedo VCR (2022) The world’s largest potato cryobank at the International Potato Center (CIP) – status quo, protocol improvement through large-scale experiments and long-term viability monitoring. Front Plant Sci 13:1059817. 10.3389/fpls.2022.1059817 PMC9746984 36523628

[CR409] Vos PG, Paulo MJ, Bourke PM, Maliepaard CA, van Eeuwijk FA, Visser RGF, van Eck HJ (2022) GWAS in tetraploid potato: identification and validation of SNP markers associated with glycoalkaloid content. Mol Breed 42:76. 10.1007/s11032-022-01344-2 PMC10248624 37313326

[CR422] Wang, L., Zhang, Z., Han, P., Liang, Y., Zhang, H., Fu, Z., Zhao, S., Yuanyuan, E., Zhang, H., Wu, X., Zhang, B., Chang, Y., Tang, K., Zheng, W., Chen, L., Wang, R., Gao, W., Hasi, A., Li, X., Bai, C. (2023). Association analysis of agronomic traits and construction of genetic networks by resequencing of 306 sugar beet (*Beta vulgaris* L*.*) lines. Sci Rep 13, 15422. 10.1038/s41598-023-42182-2 PMC10507079 37723186

[CR410] Wan X, Wu J, Wang X, Cui L, Xiao Q (2024) Accumulation patterns of flavonoids and phenolic acids in different colored sweet potato flesh revealed based on untargeted metabolomics. Food Chem X 23:101551. 10.1016/j.fochx.2024.101551 PMC11225656 38974199

[CR411] Wan X, Wang X, Zhu T, Shi G, Xiao Q (2025) Comparative metabolomics analysis of purple sweet potato flesh across different storage periods. Postharvest Biol Technol 229:113730. 10.1016/j.postharvbio.2025.113730

[CR412] Wang X, Jin Q, Wang T, Huang J, Xia Y, Yao L, Wang X (2012) Screening of glucosinolate-degrading strains and its application in improving the quality of rapeseed meal. Ann Microbiol 62:1013–1020. 10.1007/s13213-011-0341-3

[CR413] Wang H, Zhang Q, Weng B, Li F, An M, Luo T (2016) Effect of different fertilizer on accumulation, distribution and assimilation of nitrate in radish. Trans Chin Soc Agric Eng 32:148–154. 10.11975/j.issn.1002-6819.2016.z2.020

[CR414] Wang H, Wu Y, Zhang Y, Yang J, Fan W, Zhang H, Zhao S, Yuan L, Zhang P (2019) CRISPR/Cas9-based mutagenesis of starch biosynthetic genes in sweetpotato (*Ipomoea batatas*) for the improvement of starch quality. Int J Mol Sci 20:4702. 10.3390/ijms20194702 PMC6801948 31547486

[CR415] Wang Y, Wang Q, Hao W, Sun H, Zhang L (2020a) Characterization of the *OFP* gene family and its putative involvement of tuberous root shape in radish. Int J Mol Sci 21:1293. 10.3390/ijms21041293 PMC7072887 32075122

[CR416] Wang Z, Sun Y, Huang X, Li F, Liu Y, Zhu H, Liu Z, Ke W (2020b) Genetic diversity and population structure of eddoe taro in China using genome-wide SNP markers. PeerJ 8:e10485. 10.7717/peerj.10485 PMC7731653 33354429

[CR420] Wang H, Pang Z, Wang L, Tian G, Li F, Pan Y, Ding K (2024e) Localization of potato browning resistance genes based on BSA-seq technology. PeerJ 12:e17831. 10.7717/peerj.17831 PMC11313402 39131626

[CR417] Wang Q, Wang Y, Wu X, Shi W, Chen N, Pang Y, Zhang L (2024a) Sequence and epigenetic variations of R2R3-MYB transcription factors determine the diversity of taproot skin and flesh colors in different cultivated types of radish (*Raphanus sativus* L.). Theor Appl Genet 137:133. 10.1007/s00122-024-04631-y 38753199

[CR423] Wang S, Yue Z, Yu C, Wang R, Sui Y, Hou Y, Zhao Y, Zhao L, Chen C, Yang Z, Shao K (2024b) Genome-wide association study identifies the genetic basis of key agronomic traits in 207 sugar beet accessions. Hortic Res 11: uhae230. 10.1093/hr/uhae230 PMC11481341 39415969

[CR419] Wang Y-G, Hang J-X, Zhang Y-M, Su Y-T, Wang Y-H, Tan S-S, Zhang K, Liu H, Ma J, Xiong A-S, Xu Z-S (2024d) The Y locus encodes a repressor of photosynthetic genes protein that represses carotenoid biosynthesis via interaction with APRR2 in carrot. Plant Cell 36:2798–2817. 10.1093/plcell/koae111 PMC11289637 38593056

[CR418] Wang Y, Lu R-S, Li M-H, Lu X-Y, Sun X-Q, Zhang Y-M (2024c) Unraveling the molecular basis of color variation in *Dioscorea alata* tubers: integrated transcriptome and metabolomics analysis. Int J Mol Sci 25:2057. 10.3390/ijms25042057 PMC10889544 38396734

[CR421] Wang L, Shan J, Liu J, An K, Yang K, Li C, Li X, Xiong X (2025) Comparative transcriptome and metabolome profiling revealed molecular cascade events during the enzymatic browning of potato tubers after cutting. Plants (Basel) 14:1817. 10.3390/plants14121817 PMC12196699 40573805

[CR424] Waterman PG, Mole S (1994) Analysis of phenolic plant metabolites. Blackwell Scientific Publications

[CR425] Wei S, Lyu J, Wei L, Xie B, Wei J, Zhang G, Li J, Gao C, Xiao X, Yu J (2022) Chemometric approaches for the optimization of headspace-solid phase microextraction to analyze volatile compounds in coriander (*Coriandrum sativum* L.). LWT 167:113842. 10.1016/j.lwt.2022.113842

[CR426] Wei Z, Shang M, Jiang Z, Zhai H, Xing S, Wang Z, He S, Gao S, Zhao N, Zhang H, Liu Q (2025) Natural allelic variation of basic helix-loop-helix transcription factor 25 regulates carotenoid biosynthesis in sweet potato. Plant Biotechnol J 23:2627–2644. 10.1111/pbi.70086 PMC12205876 40209028

[CR427] Welsch R, Arango J, Bär C, Salazar B, Al-Babili S, Beltrán J, Chavarriaga P, Ceballos H, Tohme J, Beyer P (2010) Provitamin A accumulation in cassava (*Manihot esculenta*) roots driven by a single nucleotide polymorphism in a phytoene synthase gene. Plant Cell 22:3348–3356. 10.1105/tpc.110.077560 PMC2990137 20889914

[CR428] Wheeler EL, Ferrel RE (1971) A method for phytic acid determination in wheat and wheat fractions. Cereal Chem 48:312–320

[CR429] Wiesner M, Zrenner R, Krumbein A, Glatt H, Schreiner M (2013) Genotypic variation of the glucosinolate profile in pak choi (*Brassica rapa* ssp. *chinensis*). J Agric Food Chem 61:1943–1953. 10.1021/jf303970k 23350944

[CR430] Wilhelm J, Wojciechowski T, Postma JA, Jollet D, Heinz K, Böckem V, Müller-Linow M (2022) Assessing the storage root development of cassava with a new analysis tool. Plant Phenomics. 10.34133/2022/9767820 PMC10204708 37228350

[CR431] Witzel K, Hanschen FS, Klopsch R, Ruppel S, Schreiner M, Grosch R (2015) *Verticillium longisporum* infection induces organ-specific glucosinolate degradation in *Arabidopsis thaliana*. Front Plant Sci 6:508. 10.3389/fpls.2015.00508 PMC4498036 26217360

[CR432] Wrolstad RE, Durst RW, Lee J (2005) Tracking color and pigment changes in anthocyanin products. Trends Food Sci Technol 16:423–428

[CR433] Wu P, Jia CS, Fan S, Sun Y (2018) Principal component analysis and fuzzy comprehensive evaluation of fruit quality in cultivars of cherry. Trans Chin Soc Agric Eng 34:291–300. 10.11975/j.issn.1002-6819.2018.17.038

[CR434] Wu Y, Zhang S, Zhang H, Li F, Li G, Fan C, Sun R, Zhang S (2021) QTL mapping and candidate gene identification of swollen root formation in turnip. Int J Mol Sci 22:653. 10.3390/ijms22020653 PMC7826719 33440867

[CR435] Wu J, Ma Y, Xie R, Zhang Z, Zhang S, Wu X, Wang P, Wang D, Lu C (2024a) Integrated metabolomics and transcriptomics reveals difference involved in flavonoid and indole alkaloids biosynthesis in potato tuber flesh. Sci Hortic 324:112630. 10.1016/j.scienta.2023.112630

[CR436] Wu Y, Jin X, Wang L, Lei J, Chai S, Wang C, Zhang W, Yang X (2024b) Integrated transcriptional and metabolomic analysis of factors influencing root tuber enlargement during early sweet potato development. Genes 15:1319. 10.3390/genes15101319 PMC11507034 39457443

[CR437] Wu J, Li M, Wang W, Su Y, Li J, Gong J, Meng X, Lin C, Zhang Q, Yang Y, Xu C, Zeng L, Jiang J, Yang X (2025a) Identification and functional characterization of AsWRKY9, a WRKY transcription factor modulating alliin biosynthesis in garlic (*Allium sativum* L.). BMC Biol 23:14. 10.1186/s12915-025-02116-y PMC11731438 39806468

[CR438] Wu S, Chen T, Li Q, Wang X, Yang J, Wang D (2025b) Construction of SNP-PARMS fingerprints and analysis of genetic diversity in taro (*Colocasia esculenta*). Horticulturae 11:1224. 10.3390/horticulturae11101224

[CR439] Xiao Y, Zhu M, Gao S (2022) Genetic and genomic research on sweet potato for sustainable food and nutritional security. Genes 13:1833. 10.3390/genes13101833 PMC9602178 36292718

[CR440] Xie Y, Ying J, Xu L, Wang Y, Dong J, Chen Y, Tang M, Li C, Muleke EM, Liu L (2020) Genome-wide sRNA and mRNA transcriptomic profiling insights into dynamic regulation of taproot thickening in radish (*Raphanus sativus* L.). BMC Plant Biol 20:373. 10.1186/s12870-020-02585-z PMC7414755 32770962

[CR441] Xie Y, Ying J, Tang M, Wang Y, Xu L, Liu M, Liu L (2021) Genome-wide identification of AUX/IAA in radish and functional characterization of RsIAA33 gene during taproot thickening. Gene 795:145782. 10.1016/j.gene.2021.145782 34146634

[CR442] Xiong J, Tang X, Wei M, Yu W (2022) Comparative full-length transcriptome analysis by Oxford Nanopore Technologies reveals genes involved in anthocyanin accumulation in storage roots of sweet potatoes (*Ipomoea batatas* L.). PeerJ 10:e13688. 10.7717/peerj.13688 PMC9285475 35846886

[CR443] Xu Z-S, Huang Y, Wang F, Song X, Wang G-L, Xiong A-S (2014) Transcript profiling of structural genes involved in cyanidin-based anthocyanin biosynthesis between purple and non-purple carrot (*Daucus carota* L.) cultivars reveals distinct patterns. BMC Plant Biol 14:262. 10.1186/s12870-014-0262-y PMC4190390 25269413

[CR444] Xu F, Bao J, Kim T-S, Park Y-J (2016) Genome-wide association mapping of polyphenol contents and antioxidant capacity in whole-grain rice. J Agric Food Chem 64:4695–4703. 10.1021/acs.jafc.6b01289 27163766

[CR445] Xu Z-S, Feng K, Que F, Wang F, Xiong A-S (2017) A MYB transcription factor, DcMYB6, is involved in regulating anthocyanin biosynthesis in purple carrot taproots. Sci Rep 7:45324. 10.1038/srep45324 PMC5366895 28345675

[CR446] Xu Z-S, Yang Q-Q, Feng C, Xiong A-S (2019) Changing carrot color: insertions in *DcMYB7* alter the regulation of anthocyanin biosynthesis and modification. Plant Physiol 181:195–207. 10.1104/pp.19.00523 PMC6716245 31213511

[CR447] Xu Z-S, Yang Q-Q, Feng K, Yu X, Xiong A-S (2020) DcMYB113, a root-specific R2R3-MYB, conditions anthocyanin biosynthesis and modification in carrot. Plant Biotechnol J 18:1585–1597. 10.1111/pbi.13325 PMC7292547 31910327

[CR448] Yang J, Meyers KJ, van der Heide J, Liu RH (2004) Varietal differences in phenolic content and antioxidant and antiproliferative activities of onions. J Agric Food Chem 52:6787–6793 10.1021/jf0307144 15506817

[CR449] Yang W, Feng H, Zhang X, Zhang J, Doonan JH, Batchelor WD, Xiong L, Yan J (2020) Crop phenomics and high-throughput phenotyping: past decades, current challenges, and future perspectives. Mol Plant 13:187–214. 10.1016/j.molp.2020.01.008 31981735

[CR450] Yang Y, Zhu J, Sun L, Kong Y, Chen J, Zhu M, Xu T, Li Z, Dong T (2023) Progress on physiological and molecular mechanisms of storage root formation and development in sweetpotato. Sci Hortic 308:111588. 10.1016/j.scienta.2022.111588

[CR451] Yang H, Li Y, Hu Z, Li J (2025b) An HD-ZIP I transcription factor DZHDZ32 upregulates diosgenin biosynthesis in *Dioscorea zingiberensis*. Int J Mol Sci 26:4185. 10.3390/ijms26094185 PMC12071675 40362422

[CR452] Yang R, Huang S, Huang B Li D, Sun Y, Zhou G, Zhou D (2025a) Transcriptomic and targeted metabolomic analysis identifies genes involved in differential anthocyanin accumulation in potato tubers. Front. Plant Sci. 16:1615972. 10.3389/fpls.2025.1615972 PMC12221910 40612611

[CR453] Yao GF, Zhang SL, Wu J, Cao YF, Liu J, Han K, Yang ZJ (2011) Analysis of components and contents of soluble sugars and organic acids in ten cultivars of pear by high performance liquid chromatography. J Nanjing Agric Univ 34:25–31

[CR454] Yi G, Lim S, Chae WB, Park JE, Park HR, Lee EJ, Huh JH (2016) Root glucosinolate profiles for screening of radish (*Raphanus sativus* L.) genetic resources. J Agric Food Chem 64:61–70. 10.1021/acs.jafc.5b04575 26672790

[CR455] Yu WH, Cheng QT (2024) Accelerating yam breeding cycles with genomic selection. Genomics Appl Biol 15:319–331. 10.5376/gab.2024.15.0033

[CR456] Yu X, Li X, Guo T, Zhu C, Wu Y, Mitchell SE, Roozeboom KL, Wang D, Wang ML, Pederson GA, Tesso TT, Schnable PS, Bernardo R, Yu J (2016) Genomic prediction contributing to a promising global strategy to turbocharge gene banks. Nat Plants 2:16150. 10.1038/nplants.2016.150 27694945

[CR457] Zasoski RJ, Burau RG (1977) A rapid nitric-perchloric acid digestion method for multi element tissue analysis. Commun Soil Sci Plant Anal 8:425–436. 10.1080/00103627709366735

[CR458] Zavafer A, Bates H, Mancilla C, Ralph PJ (2023) Phenomics: conceptualization and importance for plant physiology. Trends Plant Sci 28:1004–1013. 10.1016/j.tplants.2023.03.023 37137749

[CR459] Zeng X, Liu D, Huang L (2021) Metabolome profiling of eight Chinese yam (*Dioscorea polystachya* Turcz.) varieties reveals metabolite diversity and variety specific uses. Life (Basel) 11:687. 10.3390/life11070687 PMC8308037 34357058

[CR460] Zhang N, Zhao J, Lens F, de Visser J, Menamo T, Fang W, Xiao D, Bucher J, Basnet RK, Lin K, Cheng F, Wang X, Bonnema G (2014) Morphology, carbohydrate composition and vernalization response in a genetically diverse collection of Asian and European turnips (*Brassica rapa* subsp. *rapa*). PLoS ONE 9:e114241. 10.1371/journal.pone.0114241 PMC4256417 25474111

[CR461] Zhang S-L, Deng P, Xu Y-C, Lu Y-W, Wang J-J (2016a) Quantification and analysis of anthocyanin and flavonoids compositions, and antioxidant activities in onions with three different colors. J Integr Agric 15:2175–2181

[CR471] Zhang K, Wu Z, Tang D, Lv C, Luo K, Zhao Y, Liu X, Huang Y, Wang J (2016) Development and identification of SSR markers associated with starch properties and β-carotene content in the storage root of sweet potato (*Ipomoea batatas* L.). Front Plant Sci 7:223. 10.3389/fpls.2016.00223 PMC4773602 26973669

[CR462] Zhang C, Li X, Zhan Z, Li X, Zhan Z, Cao L, Zeng A, Chang G, Liang Y (2018) Transcriptome sequencing and metabolism analysis reveals the role of cyanidin metabolism in dark-red onion (*Allium cepa* L.) bulbs. Sci Rep 8:14109. 10.1038/s41598-018-32472-5 PMC6148220 30237461

[CR464] Zhang C, Zhang YL, Bai LL, Zhao D (2020b) Optimization of ultrasonic-assisted extraction of anthocyanins from *Lonicera edulis* by response surface methodology and anti-inflammatory activity studies. Biotechnology 30:473–480. 10.16519/j.cnki.1004-311x.2020.05.0075

[CR463] Zhang X, Campbell R, Ducreux LJM, Morris J, Hedley PE, Mellado-Ortega E, Roberts AG, Stephens J, Bryan GJ, Torrance L, Chapman SN, Prat S, Taylor MA (2020a) *TERMINAL FLOWER-1/CENTRORADIALIS* inhibits tuberisation via protein interaction with the tuberigen activation complex. Plant J 103:2263–2278. 10.1111/tpj.14898 PMC7540344 32593210

[CR465] Zhang J, Zhao J, Tan Q, Qiu X, Mei S (2021) Comparative transcriptome analysis reveals key genes associated with pigmentation in radish (*Raphanus sativus* L.) skin and flesh. Sci Rep 11:11434. 10.1038/s41598-021-90633-5 PMC8169917 34075070

[CR466] Zhang J, Zhang Z-X, Wen B-Y, Jiang Y-J, He X, Bai R, Zhang X-L, Chai W-C, Xu XY, Xu J, Hou L-P, Li M-L (2023) Molecular regulatory network of anthocyanin accumulation in black radish skin as revealed by transcriptome and metabolome analysis. Int J Mol Sci 24:13663. 10.3390/ijms241713663 PMC10563070 37686469

[CR467] Zhang Y, Pu Y, Zhang Y, Li K, Bai S, Wang J, Xu M, Liu S, Zhou Z, Wu Y, Hu R, Wu Q, Kear P, Du M, Qi J (2024) Tuber transcriptome analysis reveals a novel WRKY transcription factor *StWRKY70* potentially involved in potato pigmentation. Plant Physiol Biochem 213:108792. 10.1016/j.plaphy.2024.108792 38851149

[CR468] Zhang A, Yan H, Tang W, Li C, Gao T, Song W, Gao R, Tang W, Kou M, Wang X, Zhang Y, Li Q (2025a) Transcriptomic analyses reveal the potential regulators of the storage root skin color in sweet potato. PeerJ 13:e20231. 10.7717/peerj.20231 PMC12677044 41356442

[CR469] Zhang H, Liu J, Zhao Y, Wang E, Zhou J, Chang K, Zhao X, Song B (2025c) Comprehensive metabolomics and transcriptomics analysis reveal the regulatory mechanism of StHY5 on anthocyanin accumulation in potato tubers. Plant Sci 356:112500. 10.1016/j.plantsci.2025.112500 40204193

[CR470] Zhang R, Du W, He Q, Li D, Luo B, Cao L, Wang X, Zeng Z, Wu J, Chen C (2025d) Overexpression of *StHsfA2* enhances thermotolerance and promotes tuberisation in potato under high temperature through *StSP6A*. Plant Biotechnol J 23:5045–5062. 10.1111/pbi.70283 PMC12576469 40729499

[CR472] Zhang P, Xu S, Zhang L, Li X, Qi J, Weng L, Cai S, Wang J (2025b) Metabolome and transcriptome profiling reveals light-induced anthocyanin biosynthesis and anthocyanin-related key transcription factors in Yam (*Dioscorea Alata* L.). BMC Plant Biol 25:729. 10.1186/s12870-025-06738-w PMC12123783 40442608

[CR473] Zhao S, Zhong L, Li X, Qin L, Zhou Y, Lei X, Zheng X, Jin K, Pu Z, Hou X, Song J, Lang T, Zhang C, Feng J (2024) Comparative analysis of nutrients, phytochemicals, and minerals in colored sweet potato (*Ipomoea batatas* L.) roots. Foods 13:3636. 10.3390/foods13223636 PMC11593716 39594052

[CR474] Zheng X, Zhang X, Zeng F (2025) Biological functions and health benefits of flavonoids in fruits and vegetables: a contemporary review. Foods 14:155. 10.3390/foods14020155 PMC11765039 39856822

[CR476] Zhi-Xia L, Ji-yun N, Zhen Y, Jing L, Li-Xue K, You-Ming S (2016) Optimization of 3,5-dinitrosalicylic acid colorimetry determination conditions of reducing sugar in fruits by response surface method. J Instrum Anal 35:1283–1288. 10.3969/j.issn.1004-4957.2016.10.011

[CR475] Zhishen J, Mengcheng T, Jianming W (1999) The determination of flavonoid contents in mulberry and their scavenging effects on superoxide radicals. Food Chem 64:555–559

[CR477] Zhou Y, Luo S, Hameed S, Xiao D, Zhan J, Wang A, He L (2020) Integrated mRNA and miRNA transcriptome analysis reveals a regulatory network for tuber expansion in Chinese yam (*Dioscorea opposita*). BMC Genomics 21:117. 10.1186/s12864-020-6492-5 PMC6998100 32013881

[CR478] Zhou L, Zeng X, Yang Y, Li R, Zhao Z (2024) Applications and prospects of CRISPR/Cas9 technology in the breeding of major tropical crops. Plants 13:3388. 10.3390/plants13233388 PMC11644675 39683180

[CR480] Zhu Y, Jia H, Song J, Zhang T, Zhang X, Yang W, Tan Y, Wang M, Zang J, Wang H (2024) Integrated genome-wide association and transcriptomic studies reveal genetic architecture of bulb storability of plentiful garlic germplasm resources. Hortic Res 11:uhae260. 10.1093/hr/uhae260 PMC11630311 39664692

[CR479] Zhu M, Xu H, Deng Z, Hou M, Liu L, Wang Y (2025) Identification of S-1-propenyl-*L*-cysteine as the catalytic product of AfGGT1 and a key intermediate in isoalliin biosynthesis in *Allium **fistulosum*. L Food Chem (Oxf) 10:100255. 10.1016/j.fochms.2025.100255 PMC11986493 40224724

[CR481] Zhuang H, Lou Q, Liu H, Han H, Wang Q, Tang Z, Ma Y, Wang H (2019) Differential regulation of anthocyanins in green and purple turnips revealed by combined de novo transcriptome and metabolome analysis. Int J Mol Sci 20:4387. 10.3390/ijms20184387 PMC6769466 31500111

